# Discovery of highly potent SARS-CoV-2 nsp14 methyltransferase inhibitors based on adenosine 5′-carboxamides†‡

**DOI:** 10.1039/d4md00422a

**Published:** 2024-08-01

**Authors:** Hugo Kocek, Dominika Chalupská, Milan Dejmek, Alexandra Dvořáková, Michala Zgarbová, Michal Šála, Karel Chalupský, Petra Krafčíková, Tomáš Otava, Matúš Drexler, Eliška Procházková, Blanka Klepetářová, Milan Štefek, Ján Kozic, Helena Mertlíková-Kaiserová, Evzen Boura, Jan Weber, Radim Nencka

**Affiliations:** a Institute of Organic Chemistry, and Biochemistry of the Czech Academy of Sciences Prague Czech Republic nencka@uochb.cas.cz; b Faculty of Chemical Technology, University of Chemistry and Technology Prague Czech Republic; c Department of Genetics and Microbiology, Faculty of Science, Charles University Prague Czech Republic; d Faculty of Food and Biochemical Technology, University of Chemistry and Technology Prague Czech Republic; e Department of Organic Chemistry, Faculty of Science, Charles University Prague Czech Republic

## Abstract

The emergence of SARS-CoV-2, the causative agent of COVID-19, has highlighted the need for advanced antiviral strategies. Targeting the coronaviral methyltransferase nsp14, which is essential for RNA capping, offers a promising approach for the development of small-molecule inhibitors. We designed and synthesized a series of adenosine 5′-carboxamide derivatives as potential nsp14 inhibitors and identified coumarin analogs to be particularly effective. Structural modifications revealed the importance of the 5′-carboxyl moiety for the inhibitory activity, showing superior efficacy compared to other modifications. Notably, compound 18l (**HK370**) demonstrated high selectivity and favorable *in vitro* pharmacokinetic properties and exhibited moderate antiviral activity in cell-based assays. These findings provide a robust foundation for developing targeted nsp14 inhibitors as a potential treatment for COVID-19 and related diseases.

## Abstract 2

SARS-CoV-2 nsp14 methyltransferase inhibitors based on adenosine 5′-carboxamides.

## Graphical abstract

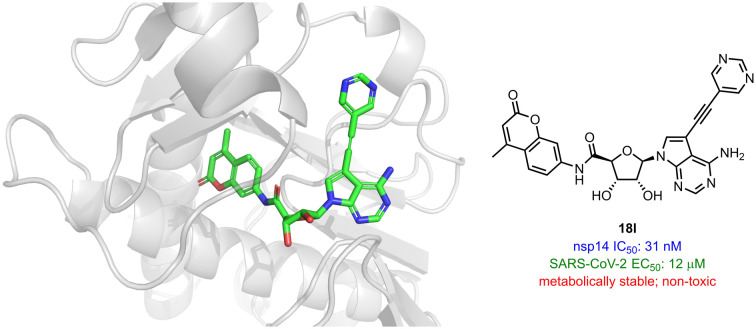

## Introduction

Viruses belonging to the family *Coronaviridae* (order *Nidovirales*) pose a significant pandemic threat, as was demonstrated by SARS-CoV, MERS-CoV and, most notably, by SARS-CoV-2 in 2002, 2012 and 2019, respectively.^1^ The genetic information of SARS-CoV-2 consists of a large (∼30 kb) positive-sense, single-stranded RNA (+ssRNA), which encodes 4 structural proteins, 16 non-structural proteins and several accessory factors.^2^ The virus replicates in double-membrane vesicles (DMVs) derived from the host endoplasmic reticulum and, therefore, does not have access to the host's mRNA capping machinery.^3^ The 5′ end of the eukaryotic mRNA is equipped with a cap-1 or cap-2 structure, which is important for several cellular processes including translation and self-recognition. The absence of this cap structure triggers the activation of cytosolic sensors (*e.g.*, IFIT1 or MDA5) and subsequently initiates an immune response.^4,5^ To mimic the host's mRNA cap, SARS-CoV-2 utilizes its own capping enzymes, including two MTases – nsp14 (*N*7 methylation; cap-0) and nsp16 (2′*O* methylation; cap-1). Both MTases are SAM-dependent, which makes them a suitable target for small-molecule inhibitors.^6^

Coronaviral MTases are a focal point of medicinal chemistry research since the beginning of the SARS-CoV-2 pandemic.^7–21^ Otava *et al.* described SAH analogs with a modified nucleobase (1) targeting the lateral cavity above the SAM-binding site^7^ and various replacements of the amino acid moiety on the 5′ end were explored by several groups.^11,22–26^ A significant portion of this research has focused on arylsulfonamides derived from 5′-aminoadenosine (2, 3), with the sulfonamide moiety being crucial for the inhibitory activity due to its specific geometry.^24–28^ Derivatives of adenosine-5′-carboxylic acid represent an under-explored area in the search for coronaviral nsp14 inhibitors. To date, only one such compound (4) has been described in the literature^11^ and is considered inferior due to its poor inhibitory activity against SARS-CoV-2 nsp14 (IC_50_ = 12 μM).

**Fig. 1:**
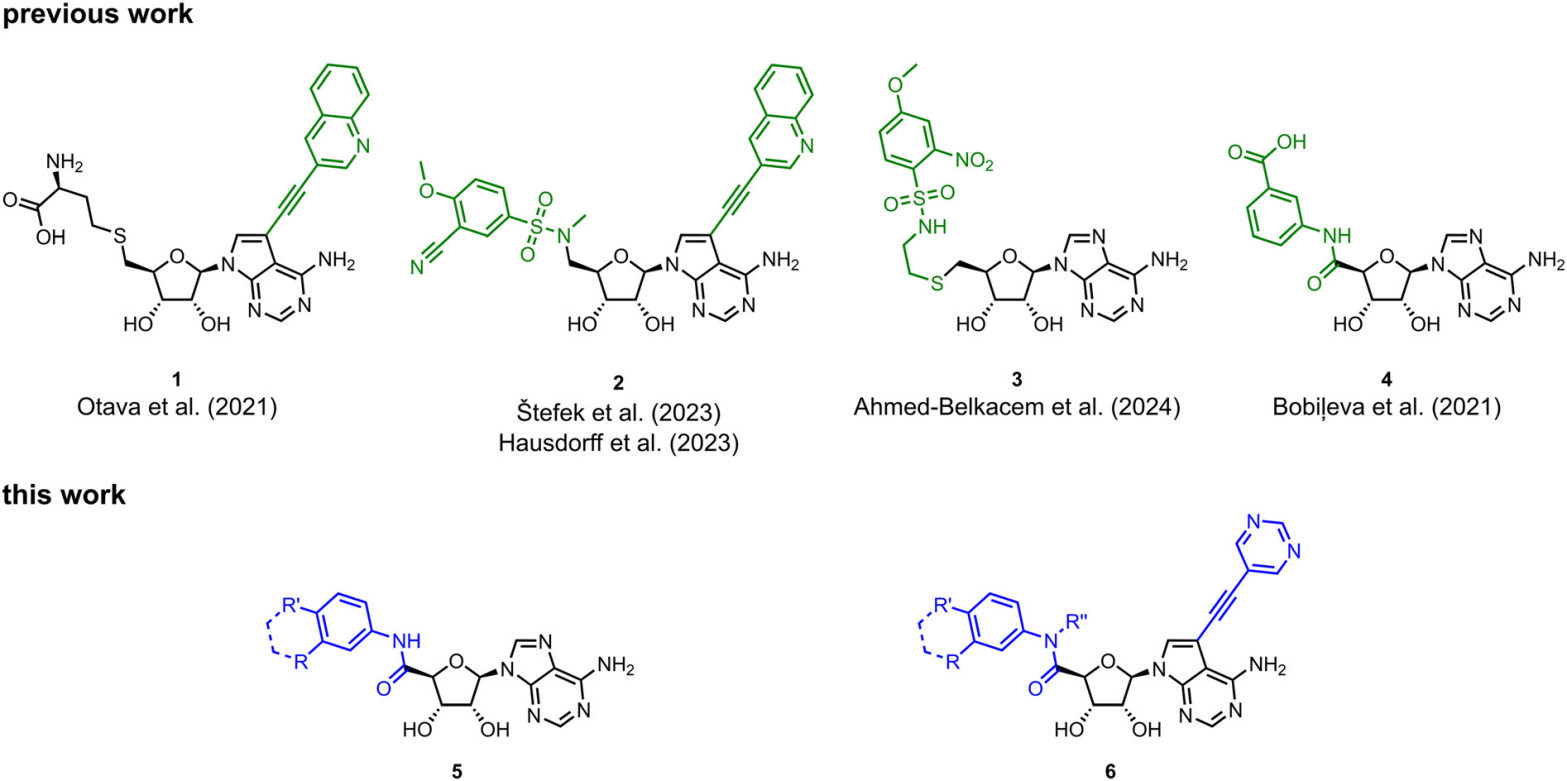
Previously described nsp14 inhibitors and structural motifs explored in this work.

In this work, we synthesized a series of amides derived from adenosine-5′-carboxylic acid leading to the development of nanomolar inhibitors with general structures 5 and 6. We further examined the importance of the amidic moiety for inhibitory activity and tested our compounds in a cell-based assay (Fig. 1).

## Results and discussion

### Synthesis

We started the synthesis from adenosine (7), which was isopropylidene-protected to afford 8.^29^ Subsequent oxidation of the 5′ carbon using TEMPO/PhI(OAc)_2_ yielded adenosine 5′-carboxylic acid 9.^30^ Treatment of 9 with SOCl_2_ afforded highly reactive intermediate 10, which was used without any purification in amidic coupling with a selected amine leading to amides 11. Final removal of the isopropylidene protecting group was achieved using 80% formic acid yielding 12.

**Scheme 1:**
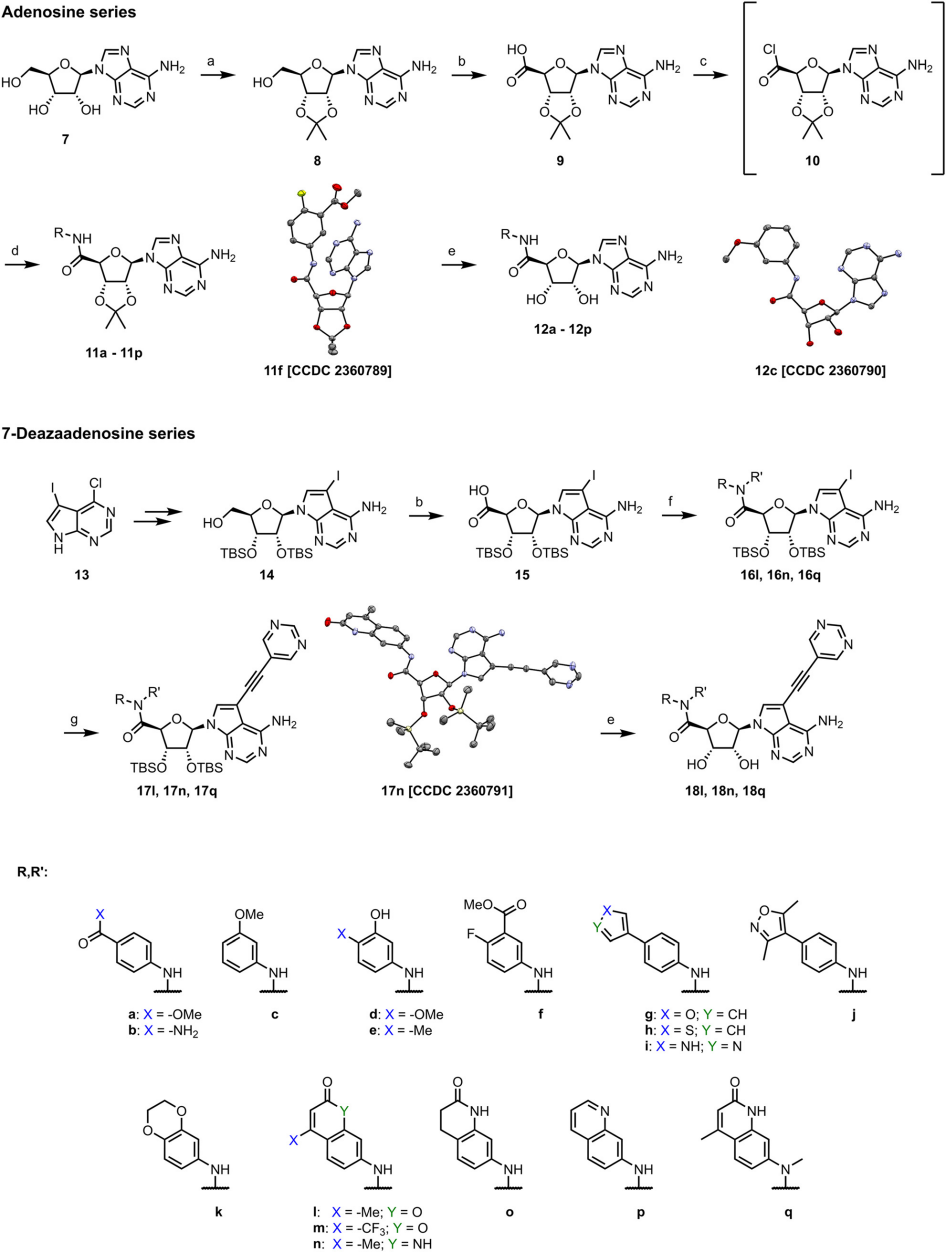
Synthesis of target compounds. (a) HClO_4_, acetone; RT, 4 h, 79%; (b) TEMPO, PhI(OAc)_2_, H_2_O, MeCN, RT, 6 h, 82%; (c) SOCl_2_, MeCN, 30 min, 40 °C; (d) i) R-NH_2_, Et_3_N, DCM, RT, 0.5–2 h, 18–95%, or i) and ii) RB(OH)_2_, Na_2_CO_3_, Pd(dppf)Cl_2_·CH_2_Cl_2_, 1,4-dioxane, water, 100 °C, 2 h, 61–79%; (e) 80% formic acid, RT, 18 h, 29–77%; (f) RR′NH; T_3_P; Et_3_N; THF, RT, 2–24 h, 42–76%; (g) 5-ethynylpyrimidine, CuI, Pd(PPh_3_)_2_Cl_2_, THF, 60 °C, 1 h, 88–95%. ORTEP diagrams of 11f, 12c, and 17n drawn at the 50% probability level; hydrogens and solvent molecules are omitted for clarity.

Synthesis of analogs with a modified nucleobase started from 2′,3′-protected 7-iodotubercidine 14, which was prepared according to a published procedure.^31^ Oxidation of the 5′ carbon was again achieved with TEMPO/PhI(OAc)_2_ yielding 15.^30^ A different approach was used for synthesis of 16, as some amines reacted poorly with acyl chloride 10. Propanephosphonic acid anhydride (T_3_P) mediated peptide coupling between the amine of choice and the acid 15 smoothly afforded products in 2 to 24 hours in moderate to good yields. Installation of the 5-ethynylpyrimidine moiety in position 7 of the nucleobase was achieved *via* the Sonogashira cross-coupling,^7^ leading to 17 which was subsequently treated with 80% formic acid to afford final compounds 18 (Scheme 1).

**Scheme 2:**
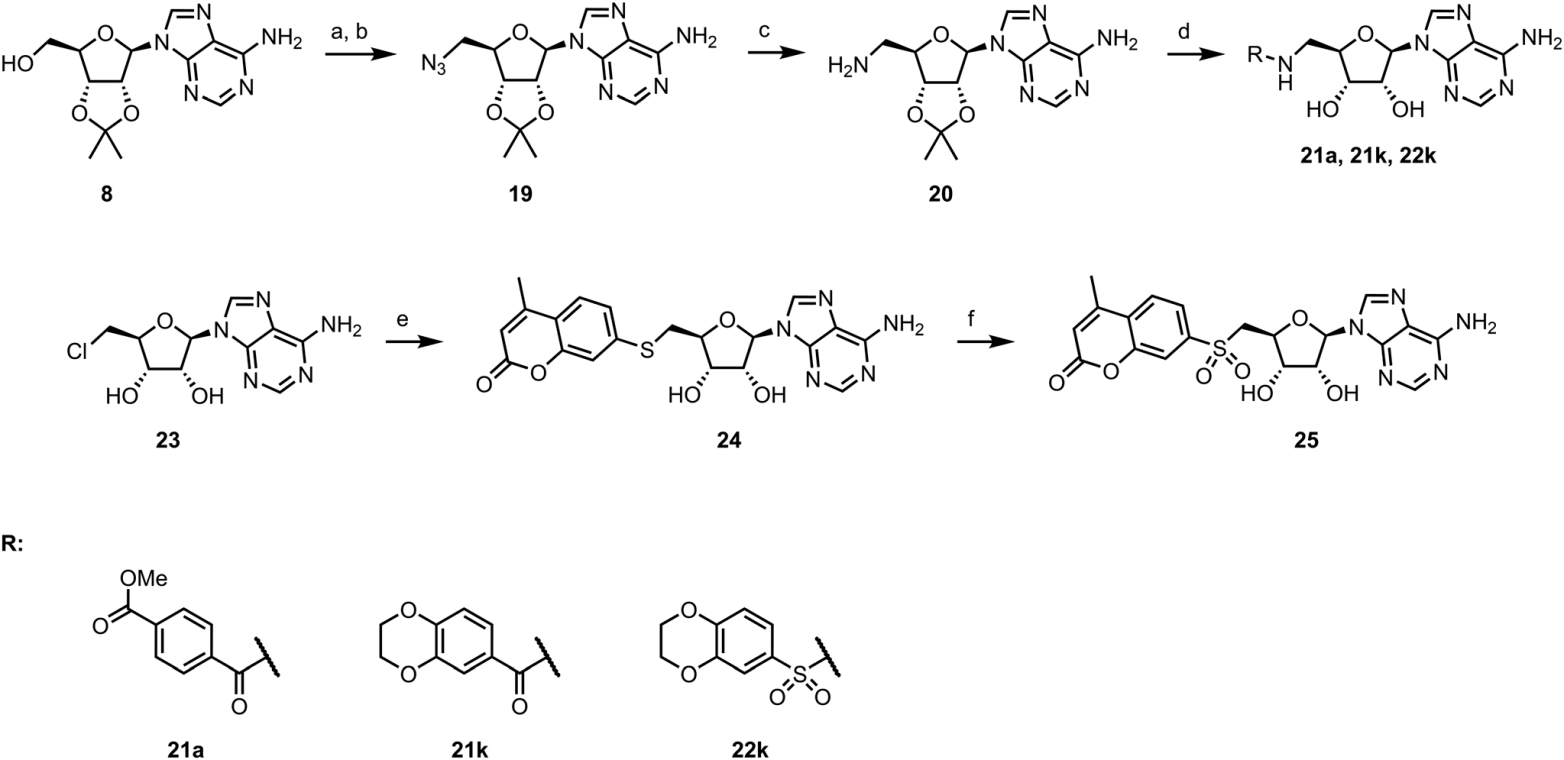
Synthesis of compounds with a modified linker. (a) MsCl, pyridine, 0 °C to RT, 1.5 h, 99%; (b) NaN_3_, DMF, 120 °C, 2 h, 70%; (c) H_2_, Pd/C, EtOH, RT, 16 h, 79%; (d) RSO_2_Cl or RCOCl, Et_3_N, DMC, 1 h, then 80% formic acid, 18 h, 14–36% over 2 steps; (e) RSH, K_2_CO_3_, EtOH, RT, 16 h, 41%; (f) Oxone®, H_2_O, RT, 4 h, 48%.

To explore other linkers, we mesylated compound 8 and, *via* nucleophilic substitution with NaN_3_, prepared compound 19. Subsequent hydrogenation yielded compound 20, which was then used for the preparation of amides (21a, 21k) and sulfonamide (22k).^32^ Finally, commercially available compound 23 was treated with 7-mercapto-4-methylcoumarin to afford compound 24. Oxidation of the sulfur linker using Oxone® yielded sulfone 25 (Scheme 2).^33^

### Inhibition of SARS-CoV-2 MTase nsp14

Initially, we focused on the synthesis of adenosine 5′-carboxamides using simple mono- and disubstituted anilines leading to 12a–f; however, these compounds exhibited only negligible activity. We then attempted to replace the ester moiety of 12a by decorating the *para* position of the aniline with various 5-membered heterocycles (compounds 12g–12j), but this resulted in inactive molecules. Introduction of a bicyclic benzo-1,4-dioxane core (compound 12k) led to an intriguing 1.8 μM inhibitory activity. Based on this result, we decided to explore further bicyclic cores *via* a scaffold hopping approach. This led to a series of coumarin-based inhibitors with exciting submicromolar potency (12l–12n).

**Fig. 2:**
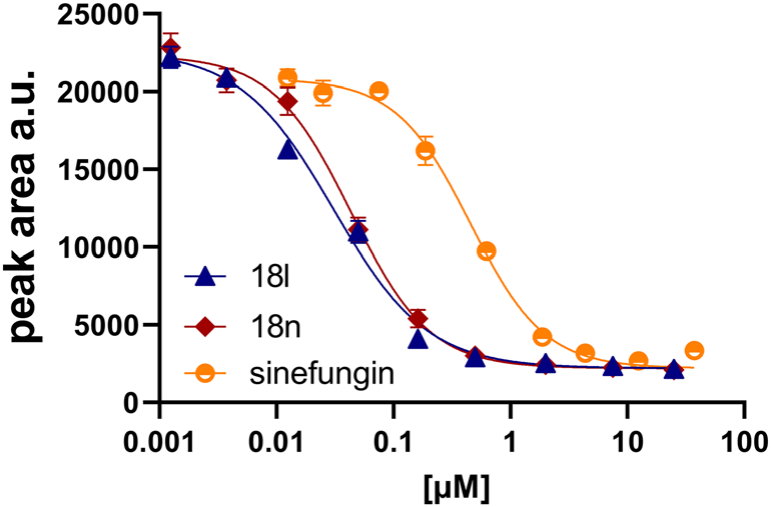
Concentration-dependent inhibition of nsp14 MTase by 18l, 18n, and sinefungin.

In recent studies,^7,8^ we identified several aromatic C-7 substituents that enhanced the biological activity of our ligands. Derivatives with the 5-ethynylpyrimidine substituent demonstrated very good inhibitory properties in all cases and our recent studies further revealed their superior solubility and metabolic stability properties (unpublished data). Consequently, we selected this substituent as our model and prepared compounds 18l and 18n, which demonstrated a 10-fold enhancement in activity in an enzymatic assay, with IC_50_ values of 30 nM and 43 nM, respectively (Fig. 2).

We also introduced a methyl group on the amidic nitrogen (18q), which has previously been shown to be highly beneficial in the case of sulfonamide 2.^8,25,28^ However, in this particular case, this modification proved to be unfavorable, resulting in a 20-fold decrease in inhibitory activity.

To better understand the role of the amidic linker, analogs bearing a reversed amidic linker, as well as sulfonamide, sulfide, and sulfone linkers, were prepared (21a, 21k, 22k, 24, 25). In all cases, this led to a significant decrease or complete loss of the inhibitory activity and clearly demonstrated the importance of the 5′-carboxyl moiety (Table 1).

**Table 1:** Inhibitory activity of synthesized compounds against SARS-CoV-2 nsp14 MTase. Sinefungin (SIN) was used as a reference inhibitor; IC_50_ (SIN) = 0.46 ± 0.05 μM; N.I., no inhibition

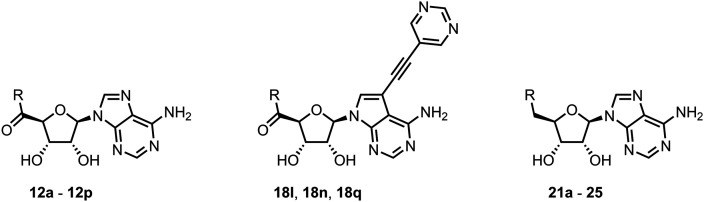								
	R	IC_50_ [μM]		R	IC_50_ [μM]		R	IC_50_ [μM]
12a	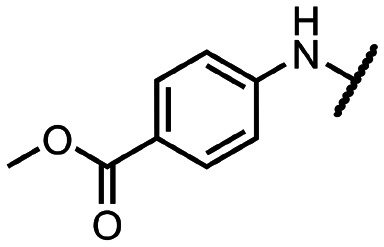	>25	12i	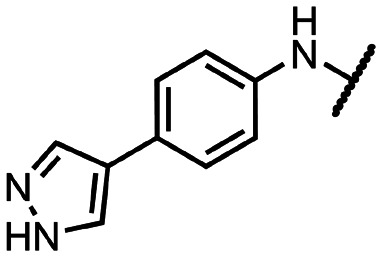	N.I.	18l	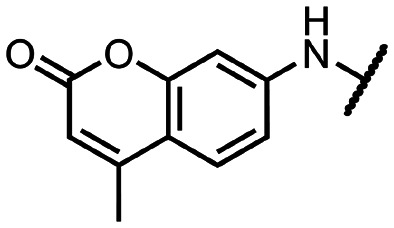	0.031 ± 0.005
12b	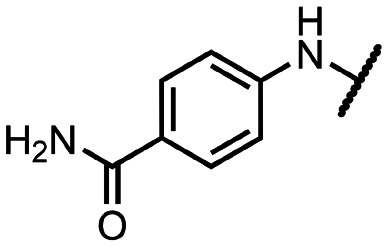	N.I.	12j	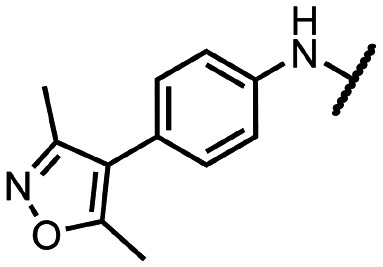	N.I.	18n	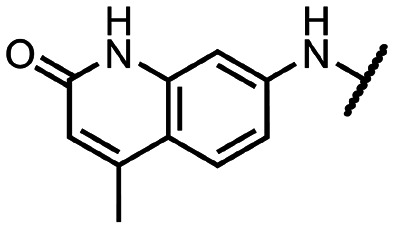	0.043 ± 0.005
12c	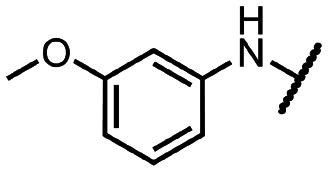	N.I.	12k	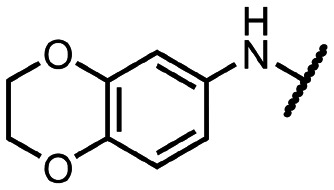	1.81 ± 0.25	18q	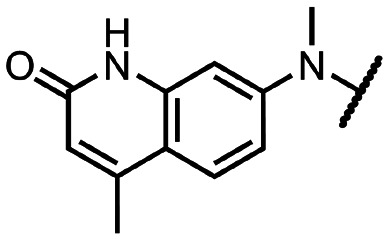	0.89 ± 0.12
12d	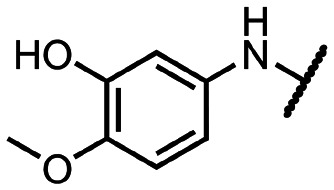	>25	12l	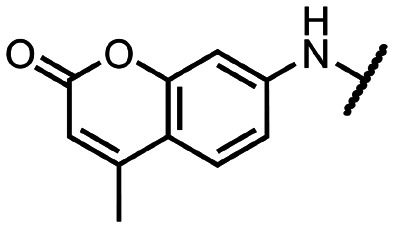	0.36 ± 0.04	21a	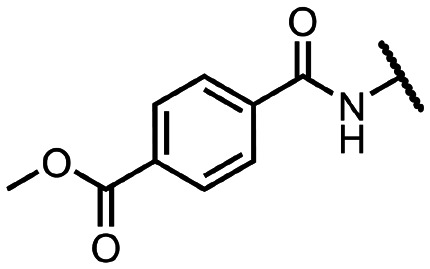	N.I.
12e	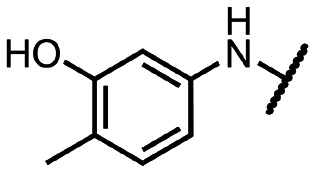	N.I.	12m	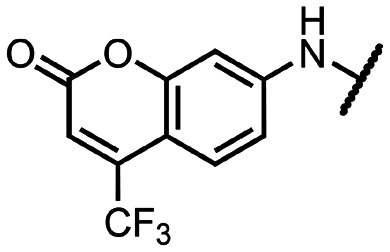	1.47 ± 0.14	21k	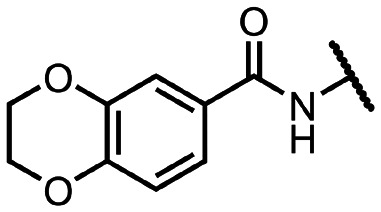	N.I.
12f	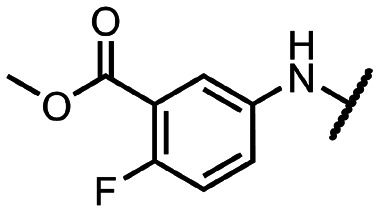	N.I.	12n	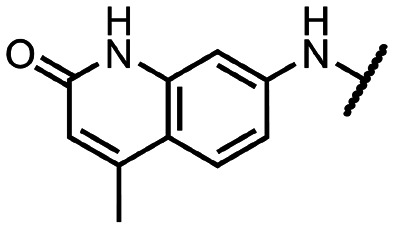	0.35 ± 0.04	22k	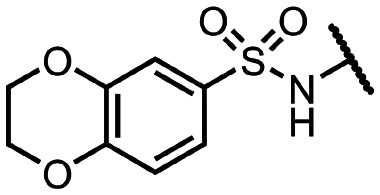	15.35 ± 2.55
12g	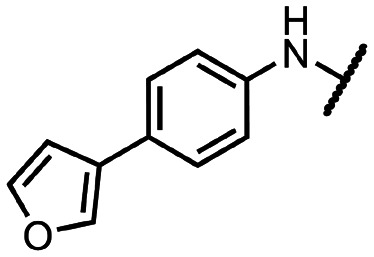	N.I.	12o	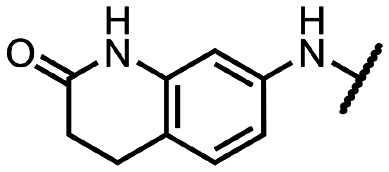	6.55 ± 1.44	24	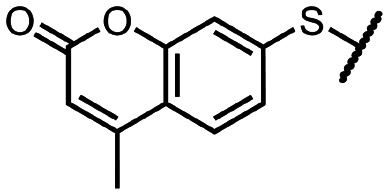	3.67 ± 0.40
12h	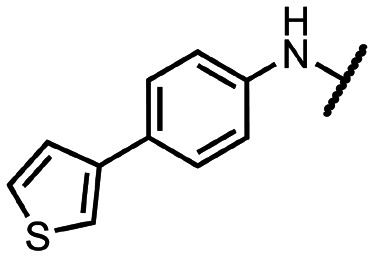	N.I.	12p	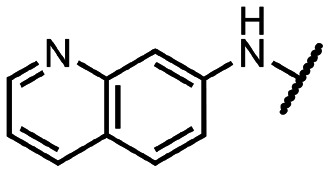	4.81 ± 2.54	25	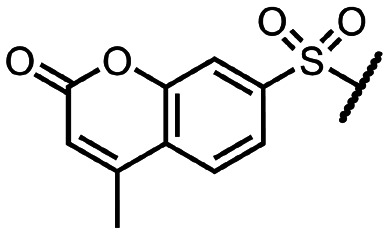	>25

Human cells rely on several MTases; therefore, it is necessary to assess the possible off-target effect. Compounds 12l, 12n, 18l, 18n, and 18q were tested against human RNMT which is an analog to coronaviral nsp14 as they both methylate the cap at position *N*7.^34^ None of the tested compounds exhibited any inhibitory activity against RNMT at 25 μM concentration of an inhibitor.

### Docking study

**Fig. 3:**
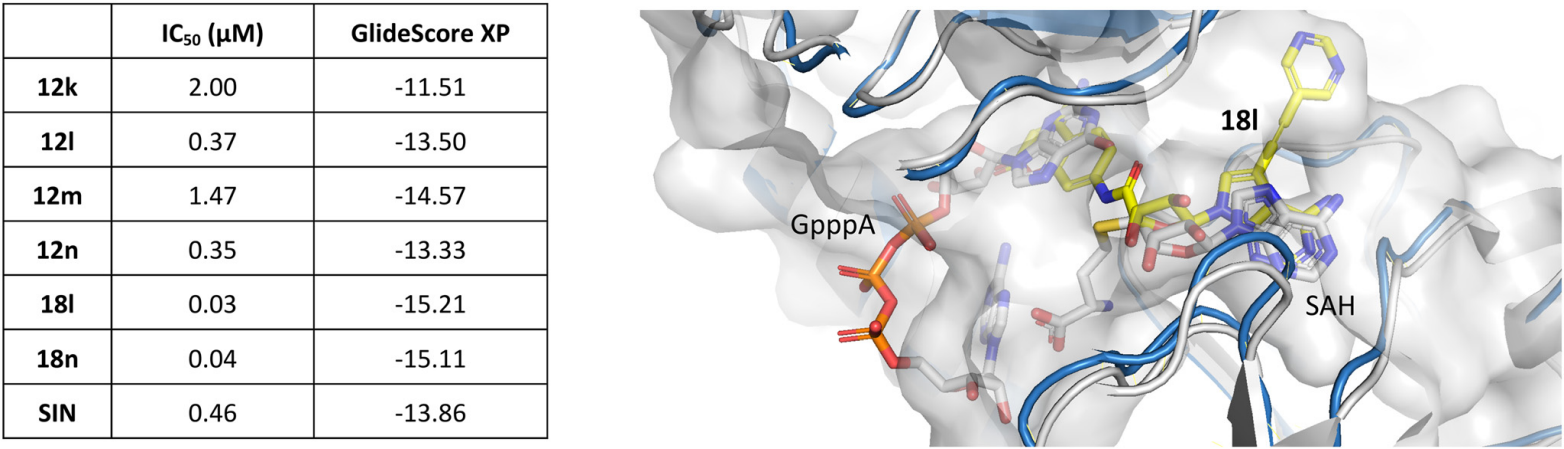
Correlation between IC_50_ values and GlideScore XP (left). Docking pose of 18l in nsp14 (PDB: 7R2V;^35^ protein in blue, inhibitor in yellow) overlaid with nsp14 with a cap analog (GpppA, gray) and SAH (PDB: 5C8S;^40^ gray) (right).

To understand the binding mode of our inhibitors, we performed extensive docking experiments using the crystal structure of SARS-CoV-2 nsp14 in complex with SAH (PDB: 7R2V).^35^ Results from Glide^36^ docking show that our inhibitors target both the SAM-binding site and the RNA-binding site as the aromatic moiety on the 5′ end extends to the cap-site and interacts with phenylalanine 426 *via* π–π stacking interaction. This amino acid residue, F426, provides one of the key interactions with the RNA cap and F426A mutants lack MTase activity.^37^ In the case of the C7-modified analogs (series 18), the alkynyl moiety extends into a lateral cavity above the SAM-binding site as described by Otava *et al.*^7^ The results of docking experiments (GlideScore XP; Fig. 3) for 12k–n, 18l, and 18n generally correlate with the measured IC_50_ values, except for compound 12m. This discrepancy might be due to the presence of fluorine atoms, as we have previously experienced that fluorinated compounds can have their GlideScore values falsely overestimated. This phenomenon has been reported by other groups as well.^38,39^

### Pharmacology

Enzymatic screening allowed us to advance our inhibitors in terms of their activity; however, pharmacological evaluation is a crucial step towards compounds capable of inhibiting viral replication in cells. Compounds 12l, 12m, 12n, 18l, 18n and 18q were evaluated for their stability in both human and mouse plasma and liver microsomes (Table 2). All tested compounds were stable in liver microsomes and only 12m and 18n were metabolized in plasma. Inhibitor 12m was shown to be unstable in both human and mouse plasma, potentially due to the strong electron-withdrawing effect of –CF_3_ in conjugation with the lactone moiety. In the case of 18n, metabolic instability was observed only in mouse plasma with a half-life of 94 minutes (see the ESI‡ for graphs, Fig. S2).

**Table 2:** Metabolic stability of selected compounds in plasma and liver microsomes and their Caco-2 permeability. Propantheline, verapamil, and digoxin were used as control compounds for the respective assays. N.D., not determined

	Plasma stability % of the parent compound after 120 min		Microsomal stability % of the parent compound after 45 min		Caco-2 permeability		
	Human	Mouse	Human	Mouse	Papp (cm s^−1^) *A*–*B* × 10^−6^	Papp (cm s^−1^) *B*–*A* × 10^−6^	Efflux ratio
12l	103 ± 3.3	93 ± 2.7	104 ± 4.1	95 ± 5.0	3.1 ± 1.3	10.7 ± 2.5	3.4
12m	20 ± 1.4	67 ± 3.5	90 ± 9.1	93 ± 9.4	8.0 ± 2.9	32.9 ± 5.3	4.1
12n	97 ± 2.0	78 ± 0.3	95 ± 0.0	90 ± 2.9	4.4 ± 1.8	2.2 ± 0.4	0.5
18l	74 ± 0.2	89 ± 1.1	90 ± 4.2	84 ± 2.5	10.1 ± 4.0	8.3 ± 0.5	0.8
18n	93 ± 0.4	41 ± 0.7	90 ± 2.0	85 ± 0.6	11.3 ± 3.7	7.3 ± 1.3	0.6
18q	80 ± 1.1	86 ± 0.9	107 ± 6.6	117 ± 10.9	2.0 ± 0.3	2.1 ± 0.4	1.0
Propantheline	0.1 ± 0.0	9 ± 0.2	N.D.	N.D.	N.D.	N.D.	N.D.
Verapamil	N.D.	N.D.	32 ± 1.1	5 ± 0.2	N.D.	N.D.	N.D.
Digoxin	N.D.	N.D.	N.D.	N.D.	1.7 ± 0.2	13.8 ± 0.9	8.5

Monolayers of differentiated Caco-2 epithelial cells were used to simulate the intestinal absorption^41,42^ of selected inhibitors and all tested compounds exhibited reasonable transepithelial permeability. Two compounds exhibited an efflux ratio (ER) above 2, 12l (ER: 3.4) and 12m (ER: 4.1), suggesting their interaction with cellular efflux pumps (Table 2).^43^

### Inhibition of SARS-CoV-2 *in vitro*

**Fig. 4:**
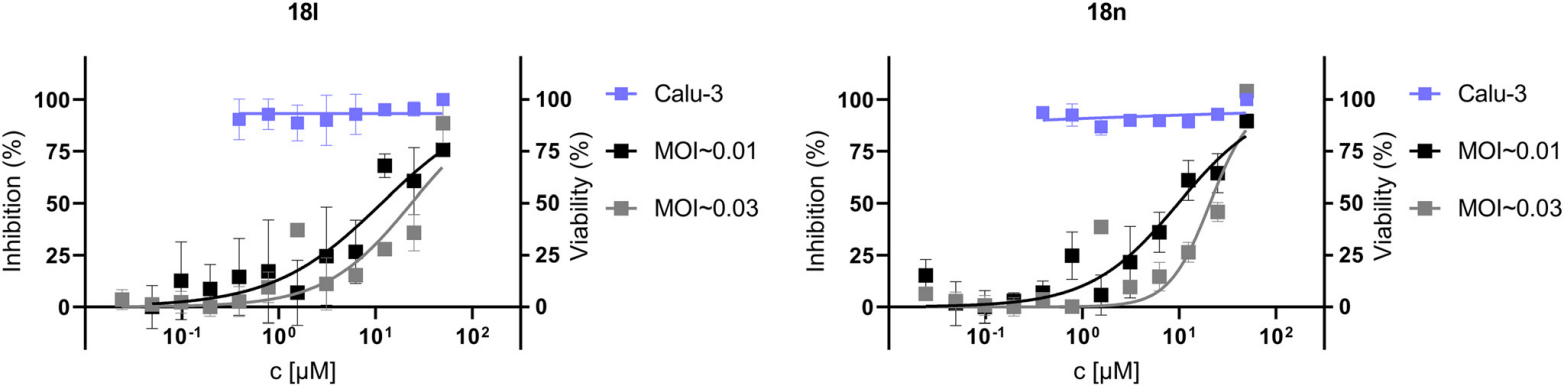
Antiviral effect of compounds 18l and 18n against SARS-CoV-2 (black and gray lines) in the Calu-3 cell line. The cell viability without virus in the presence of compounds is shown by the blue line. Plots were created in GraphPad Prism software version 10.2.3. and EC_50_ values were calculated using nonlinear regression.

We tested the cytotoxicity and the anti-SARS-CoV-2 activity of compounds 12l, 12n, 18l, 18n, and 18q in two types of cell cultures – Calu-3 and Vero E6. The compounds were prepared in 2-fold serial dilutions, starting from a 50 μM solution. Cells were pretreated with the inhibitors two hours prior to the infection with the SARS-CoV-2 strain hCoV-19/Czech Republic/NRL_6632_2/2020 at two different multiplicities of infection (MOI ∼0.03 and MOI ∼0.01). Simultaneously, the same experimental setup without virus was used to determine the compounds' cytotoxicity. Cells were incubated for three days at 37 °C in 5% CO_2_. Cell viability was then assessed using the XTT assay.^44^ We detected an antiviral effect for compounds 18l and 18n in the Calu-3 cell line, with EC_50_ values of 12 ± 6 μM and 10 ± 3 μM, respectively, at an MOI ∼0.01, without any observed cytotoxicity (CC_50_ > 50 μM; Fig. 4). For compounds 12l, 12n, and 18q, the EC_50_ values were determined to be above 50 μM in the Calu-3 cell line (see the ESI‡ for graphs, Fig. S4). No antiviral effect was observed in the Vero E6 cell line for any of the five inhibitors which may be explained by the defective interferon response of this cell line.^45^

## Conclusion

In this work, we describe the discovery of SARS-CoV-2 nsp14 inhibitors with novel structural motifs based on adenosine 5′-carboxamides derived from bicyclic amines. Through scaffold hopping, we found out that coumarin analogs are particularly effective. We also explored the importance of the 5′-carboxyl moiety for inhibitory activity. Analogs bearing a methylated amide, reversed amidic linker, sulfonamide, sulfide, or sulfone showed a significant decrease or complete loss of the inhibitory activity. Compound 18l (**HK370**), with an IC_50_ value of 31 nM, exhibited high selectivity for nsp14 over human RNMT (IC_50_ (RNMT) > 25 μM; CC_50_ > 50 μM), a favorable *in vitro* metabolic profile and good transepithelial permeability. Notably, our inhibitors show moderate efficacy in a cell-based assay (EC_50_: 12 ± 6 μM, Calu-3 cell line). Overall, this work provides a strong foundation for the development of targeted nsp14 inhibitors as potential treatments for COVID-19 and other coronavirus-related diseases.

## Abbreviations

SARS-CoV: Severe acute respiratory syndrome-related coronavirus
MERS-CoV: Middle East respiratory syndrome coronavirus
COVID-19: Coronavirus disease 2019
+ssRNA: Single-stranded positive-sense RNA
MTase: Methyltransferase
SAM: *S*-Adenosyl-l-methionine
SAH: *S*-Adenosyl-l-homocysteine
ER: Efflux ratio
MOI: Multiplicity of infection

## Author contributions

H. K., M. D., M. Š., T. O., and M. Š.: synthesis of the compounds; D. C., A. D., K. C., P. K., M. D., and J. K.: protein purification and biochemical assays; A. D. and M. Z.: cell-based assays; E. P.: compound characterization by NMR spectroscopy; B. K.: SC-XRD; H. K. and M. D.: writing – original draft; H. M.-K., E. B., J. W., and R. N.: supervision, funding acquisition, project administration, and writing – review & editing.

## Conflicts of interest

There are no conflicts to declare.

## Supplementary Material

MD-015-D4MD00422A-s001

MD-015-D4MD00422A-s002

## Data Availability

The data supporting this article have been included as part of the ESI.‡ Crystallographic data for 11f, 12c, and 17n have been deposited at the CCDC under 2360789, 2360790, and 2360791.
