# Supplementary material for: Discovery of highly potent SARS-CoV-2 nsp14 methyltransferase inhibitors based on adenosine 5′-carboxamides
Source: RSC Med Chem. 2024 Aug 1;15(10):3469–76. doi: 10.1039/d4md00422a (PMC11352099; doi:10.1039/d4md00422a)
Supplement: MD-015-D4MD00422A-s001 [file MD-015-D4MD00422A-s001.pdf]

Supplementary Information (SI) for RSC Medicinal Chemistry.
This journal is © The Royal Society of Chemistry 2024

Electronic supplementary information

Discovery of highly potent SARS-CoV-2 nsp14 methyltransferase inhibitors based on adenosine 5′-carboxamides

Hugo Kocek[a,b], Dominika Chalupská[a], Milan Dejmek[a], Alexandra Dvořáková[a], Michala Zgarbová[a,c], Michal Šála[a], Karel Chalupský[a], Petra Krafčíková[a], Tomáš Otava[a,d], Matúš Drexler[a], Eliška Procházková[a], Blanka Klepetářová[a], Milan Štefek[a,e], Ján Kozic[a], Helena Mertlíková-Kaiserová[a], Evzen Boura[a], Jan Weber[a], Radim Nencka[a,*]

[a] Institute of Organic Chemistry and Biochemistry of the Czech Academy of Sciences, Prague, Czech Republic.

[b] Faculty of Chemical Technology, University of Chemistry and Technology, Prague, Czech Republic

[c] Department of Genetics and Microbiology, Faculty of Science, Charles University, Prague, Czech Republic

[d] Faculty of Food and Biochemical Technology, University of Chemistry and Technology, Prague, Czech Republic

[e] Department of Organic Chemistry, Faculty of Science, Charles University, Prague, Czech Republic

Email: nencka@uochb.cas.cz

Contents

1. General Information 3
  1.1. Synthesis 3
  1.2. Instrumental methods 3
2. Synthesis 5
3. Biological data 48
  3.1. Nsp14 protein expression and purification 48
  3.2. Methyltransferase activity assay 48
  3.3. Plasma stability assay 51
  3.4. Microsomal stability 51
  3.5. Caco-2 permeability assay 54
  3.6. Antiviral and cytotoxicity assays 55
4. ^{1}H, ^{13}C, and ^{19}F NMR spectra of final compounds 57
5. UPLC purity of final compounds 83
6. X-ray crystallography 108
7. Docking study 111
8. References 112

1. General Information

1.1. Synthesis

Unless stated otherwise, all solvents were evaporated at 40 °C at 2 kPa, and the compounds were dried at 40 °C at 2 kPa. Starting compounds and reagents were purchased from commercial suppliers (Sigma-Aldrich, Fluorochem, AmBeed, TCI) and used without further purification. Acetonitrile was dried using activated 3Å molecular sieves. Dry dichloromethane was distilled from P_2O_5 and stored over 3Å molecular sieves.

1.2. Instrumental methods

Analytical High-Performance Liquid Chromatography (HPLC), low resolution mass spectra, UV absorbance and compound purity were measured on a Waters Ultra-high Performance Liquid Chromatography-Mass Spectrometry (UPLC-MS) system consisting of a Waters UPLC H-Class Core System, a UPLC photodiode array (PDA) detector and a Waters SQD2 or QDa mass spectrometer. The MS method used was electrospray ionization $(ESI)^+$ and/or $(ESI)^-$, cone voltage = 15 V, mass detector range 105–1000 Da or 100–1250 Da. Waters Cortecs UPLC C18 column, 1.6 µm, 2.1 × 50 mm was used with LC method: H_2O/MeCN, 0.1% formic acid as a modifier, gradient 0–100 %, run length 3.5 min, flow 0.7 ml/min).

Both normal **flash column chromatography** (using VWR International Silica gel 60, particle size 0.040–0.063 mm) and reverse-phase flash column chromatography (using C18 RediSep Rf columns) were conducted with a Combiflash® Rf system from Teledyne ISCO.

Purity of the final compounds was determined by UPLC MS and was 95% or higher. **Optical rotations** were measured in DMSO on Autopol IV polarimeter (Rudolph Research Analytical), $[\alpha]_D^{20}$ values are given in 10^{-1} deg·cm^2·g^{-1}. **High resolution mass spectra** were measured on LTQ Orbitrap XL (Thermo Fisher Scientific). The **melting points** of the final compounds used for biological testing were determined with a Büchi Melting Point Apparatus B-540 using open capillaries.

NMR spectra were measured on a Bruker Avance III NMR spectrometer equipped with a broad-band PRODIGY cryo-probe with ATM module (5 mm CPBBO BB-1H/19F/D Z-GRD) operating at 400 MHz for ^{1}H and 101 MHz for ^{13}C, on a Bruker Avance III NMR spectrometer equipped with a broad-band probe (5 mm BBO-1H Z-GRD) operating at 400 MHz for ^{1}H and 101 MHz for ^{13}C, or on a Bruker Avance III NMR spectrometer equipped with a broad-band cryo-probe probe with ATM module

(5 mm CPBBO BB-$^{1}H/^{19}F/^{15}N$/D Z/GRD) operating at 500 MHz for ^{1}H, 126 MHz for ^{13}C. All the samples were measured in DMSO-d_6 at 25 °C and the spectra were referenced to the residual solvent signal, [δ (^{1}H) = 2.50 ppm, δ (^{13}C) = 39.5 ppm]. Chemical shifts are given in ppm (δ-scale), coupling constants (J) in Hz. NMR signal assignment was done using a combination of 1D and 2D (H,H-COSY, H,C-HSQC and H,C-HMBC) spectra. The NMR experiments were performed by the standard pulse sequences from Bruker and the NMR data were processed in Bruker software Topspin 3.6. Nucleobase and ribose moiety of final compounds are numbered as indicated in **S1**. For numbering of 5' and C7 modifications see specific atom numbering for the corresponding compound.

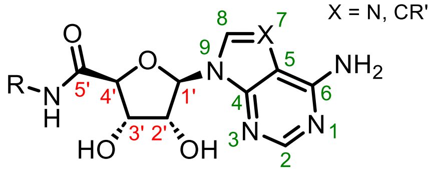

S1

Single-crystal X-ray diffraction data were collected at 180 K using Bruker D8 VENTURE system equipped with a Photon 100 CMOS detector, a multilayer monochromator, and a CuKα Incoatec microfocus sealed tube (λ = 1.54178 Å). The frames were integrated with the with Bruker SAINT[1] software package. The structures were solved by direct methods with SIR92[2] and were refined by full-matrix least-squares on F with CRYSTALS.[3] The positional and anisotropic thermal parameters of all non-hydrogen atoms were refined. All hydrogen atoms were located in a difference Fourier map, but those attached to carbon atoms were repositioned geometrically. They were initially refined with soft restraints on the bond lengths and angles to regularize their geometry, then their positions were refined with riding constraints.

2. Synthesis

((3a*R*,4*R*,6*R*,6a*R*)-6-(6-Amino-9*H*-purin-9-yl)-2,2-dimethyltetrahydrofuro[3,4-*d*][1,3]dioxol-4-yl)methanol (**8**)

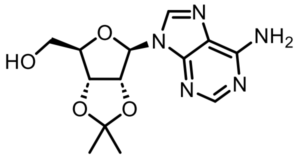

Compound **8** was prepared according to a published procedure.[4] Adenosine (**7**; 20 g, 75 mmol) was mixed with dry acetone (1200 mL) and 70% $HClO_4$ (9 mL). After 4 hours, the reaction mixture was concentrated to one-quarter of its volume, the precipitated product was collected on a sintered funnel and washed with chilled acetone. Compound **8** (19.48 g, yield 85%) was obtained as a white solid.

^{1}H NMR (400 MHz, DMSO-d_6) δ 8.34 (s, 1H), 8.16 (s, 1H), 7.34 (s, 2H), 6.12 (d, *J* = 3.2 Hz, 1H), 5.34 (dd, *J* = 6.1, 3.1 Hz, 1H), 4.96 (m, 1H), 4.22 (m, 1H), 3.55 (m, 2H), 1.54 (s, 3H), 1.32 (s, 3H) ppm. **^{13}C NMR** (101 MHz, DMSO-d_6) δ 156.13, 152.62, 148.80, 139.68, 119.10, 113.03, 89.61, 86.34, 83.21, 81.34, 61.57, 27.06, 25.17 ppm. **HRMS** (ESI) Calculated for $C_{13}H_{16}O_4N_5$, $[M - H]^-$ m/z: 306.12078, found 306.12057.

(3a*S*,4*S*,6*R*,6a*R*)-6-(6-Amino-9*H*-purin-9-yl)-2,2-dimethyltetrahydrofuro[3,4-*d*][1,3]dioxol-4-carboxylic acid (**9**)

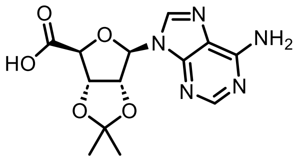

Compound **9** was prepared according to a published procedure.[5] Compound **8** (15 g, 48.8 mmol) was mixed with $PhI(OAc)_2$ (34.59 g, 107.38 mmol, 2.2 eq.), TEMPO (1.53 g, 9.76 mmol, 0.2 eq.), and H_2O/MeCN (98 mL, 1:1). The reaction mixture was stirred for 4 hours, after which the precipitated acid **9** was filtered using filter paper and washed first with acetonitrile and then with diethyl ether. Compound **9** (12.17 g, yield 78%) was obtained as a white solid.

^{1}H NMR (400 MHz, DMSO-d_6) δ 12.78 (s, 1H), 8.24 (s, 1H), 8.08 (s, 1H), 7.27 (s, 2H), 6.33 (s, 1H), 5.54 (dd, *J* = 6.0, 2.0 Hz, 1H), 5.47 (d, *J* = 6.0 Hz, 1H), 4.68 (d, *J* = 1.9 Hz, 1H), 1.52 (s, 3H), 1.35 (s, 3H) ppm. **^{13}C NMR** (101 MHz, DMSO-d_6) δ 170.65, 155.98, 152.32, 149.11, 140.33, 118.74, 112.65, 89.47, 85.37, 83.74, 83.38, 26.47, 24.90 ppm. **HRMS** (ESI) Calculated for $C_{13}H_{14}O_5N_5$, $[M - H]^-$ m/z: 320.10004, found 320.09981.

(2*S*,3*R*,4*R*,5*R*)-5-(4-Amino-5-iodo-7*H*-pyrrolo[2,3-*d*]pyrimidin-7-yl)-3,4-bis((tert-butyldimethylsilyl)oxy)tetrahydrofuran-2-carboxylic acid (**15**)

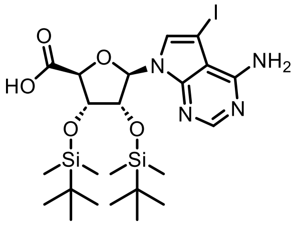

Synthesis of **15** was based on a published procedure.[5] Compound **14** (1.555 g, 2.505 mmol; prepared according to a published procedure[6]) was mixed with $PhI(OAc)_2$ (1.775 g, 5.512 mmol, 2.2 eq.), TEMPO (78.2 mg, 0.501 mmol, 0.2 eq.), and H_2O/MeCN (5 mL, 1:1). The reaction mixture was stirred for 2 hours. Then, the reaction mixture was evaporated and purified using flash column chromatography (cyclohexane/EtOAc, 5 to 50 %, 0,5 % of AcOH present in EtOAc) to afford **15** (850 mg, yield 52 %) as an off-white solid.

^{1}H NMR (400 MHz, DMSO) δ 13.71–13.55 (bs, 1H), 8.12 (s, 1H), 7.77 (s, 1H), 6.89–6.72 (bs, 2H), 6.22 (d, *J* = 7.1 Hz, 1H), 4.47–4.36 (m, 3H), 0.93 (s, 9H), 0.65 (s, 9H), 0.16 (s, 3H), 0.13 (s, 3H), -0.13 (s, 3H), -0.48 (s, 3H) ppm. **^{13}C NMR** (101 MHz, DMSO) δ 171.53, 157.17, 152.07, 150.43, 126.84, 103.07, 86.06, 83.02, 75.35, 75.12, 52.69, 25.64, 25.33, 17.78, 17.43, -4.86, -4.95, -4.99, -6.05 ppm. **HRMS** (ESI) Calculated for $C_{23}H_{40}O_5N_4ISi_2$, $[M + H]^+$ m/z: 635.15764, found 635.15752.

9-((3a*R*,4*R*,6*R*,6a*R*)-6-(Azidomethyl)-2,2-dimethyltetrahydrofuro[3,4-*d*][1,3]dioxol-4-yl)-9*H*-purin-6-amine (19)

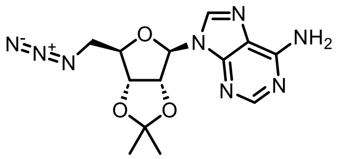

Compound **19** was prepared according to a published procedure.[7] Compound **8** (8 g, 26.0 mmol) was first co-distilled with dry pyridine (130 mL) and then dissolved in dry pyridine (130 mL). The solution was stirred in an ice bath for 30 minutes, after which mesyl chloride (2.4 mL, 31.2 mmol) was gradually added. After 90 minutes, only mesylated intermediate was observed on UPLC MS analysis. Volatiles were evaporated and the residue was mixed with EtOAc. The mixture was washed with brine, and the organic phase was dried over Na_2SO_4. The solution was evaporated to dryness and the crude product was used in the subsequent reaction without further purification.

The mesylated intermediate was dissolved in DMF (104 mL) along with NaN_3 (6.761 g, 104 mmol, 4 eq.). The reaction mixture was heated with stirring at 80 °C for 16 hours. Volatiles were evaporated, the residue was dissolved in EtOAc and washed with brine. The organic phase was dried over Na_2SO_4, evaporated and purified using flash column chromatography (EtOAc/EtOH; 0 to 10%) to afford **19** (6.03 g, yield 70%) as a white solid.

^{1}H NMR (400 MHz, DMSO-*d*$_6$) δ 8.34 (s, 1H), 8.18 (s, 1H), 7.34 (s, 2H), 6.21 (d, *J* = 2.6 Hz, 1H), 5.52 (dd, *J* = 6.3, 2.7 Hz, 1H), 5.00 (dd, *J* = 6.3, 3.1 Hz, 1H), 4.30 (ddd, *J* = 7.5, 4.8, 3.1 Hz, 1H), 3.62 (dd, *J* = 13.0, 7.2 Hz, 1H), 3.54 (dd, *J* = 13.0, 4.8 Hz, 1H), 1.54 (s, 3H), 1.33 (s, 3H) ppm. **^{13}C NMR** (101 MHz, DMSO) δ 156.13, 152.75, 148.75, 139.97, 119.13, 113.52, 89.05, 84.48, 82.88, 81.50, 51.50, 26.93, 25.15 ppm. **UPLC-MS** (ESI) Calculated for $C_{13}H_{17}N_8O_3$, $[M + H]^+$ m/z: 333.14, found 333.11.

9-((3a*R*,4*R*,6*R*,6a*R*)-6-(Aminomethyl)-2,2-dimethyltetrahydrofuro[3,4-*d*][1,3]dioxol-4-yl)-9*H*-purin-6-amin (20)

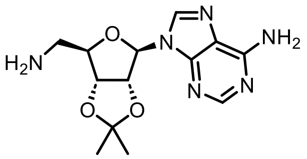

Compound **19** (6.0 g, 18.1 mmol) was dissolved in EtOH (180 mL), and the flask was backfilled with argon five times. Pd/C (10 % wt., 302 mg) was added, and a hydrogen balloon was attached (refilled as needed during the reaction). The reaction mixture was stirred for 20 hours at room temperature. The catalyst was filtered off on a pad of celite, and the filtrate was evaporated to afford **20** (3.89 g, yield 70%) as a light-yellow solid.

^{1}H NMR (400 MHz, DMSO-d_6) δ 8.37 (s, 1H), 8.16 (s, 1H), 7.33 (s, 2H), 6.08 (d, *J* = 3.3 Hz, 1H), 5.45 (dd, *J* = 6.3, 3.2 Hz, 1H), 4.98 (dd, *J* = 6.3, 2.8 Hz, 1H), 4.09 (td, *J* = 5.7, 2.8 Hz, 1H), 2.69 (m, 2H), 1.53 (s, 3H), 1.32 (s, 3H) ppm. **^{13}C NMR** (101 MHz, DMSO-d_6) δ 156.13, 152.71, 148.96, 139.95, 119.15, 113.13, 89.10, 86.87, 82.69, 81.59, 43.58, 27.06, 25.23 ppm. **HRMS** (ESI) Calculated for $C_{13}H_{19}O_3N_6$, $[M + H]^+$ m/z: 307.15131, found 307.15136.

***General procedure A*: synthesis of amides via $SOCl_2$ activation** (**11a–11f; 11k–p**)

To a suspension of acid **9** (300 mg, 0.93 mmol, 1 eq.) in dry acetonitrile (12 mL), $SOCl_2$ (120 µL, 1.6 mmol, 1.7 eq.) was added dropwise. The suspension was stirred at 40 °C for 40 minutes. Following this, the solvent was evaporated to yield a highly reactive **10**. Immediately after evaporation, **10** was dissolved in dry dichloromethane (20 mL), and corresponding amine (1 mmol, 1.1 eq.) followed by Et_3N (285 µL, 2.1 mmol, 2.2 eq.) were added. Conversion of **10** to **11** typically occurred within two hours. The resulting solution was then evaporated, adsorbed on silica gel and purified via reverse-phase C18 flash chromatography (H_2O/MeCN, 5 to 60 %).

Methyl 4-((3a*S*,4*S*,6*R*,6a*R*)-6-(6-amino-9*H*-purin-9-yl)-2,2-dimethyltetrahydrofuro[3,4-*d*][1,3]dioxol-4-carboxamido)benzoate (**11a**)

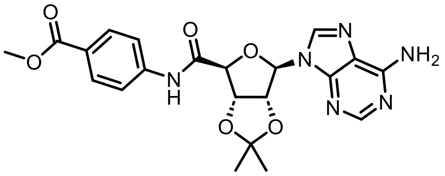

Obtained as a white solid (322 mg, yield 65 %).

^{1}H NMR (400 MHz, DMSO-*d₆*) δ 11.96 (s, 1H), 9.70 (s, 1H), 8.30 (s, 1H), 7.94 (s, 1H), 7.81–7.73 (m, 2H), 7.44–7.36 (m, 2H), 7.19 (s, 2H), 6.46 (d, *J* = 1.5 Hz, 1H), 5.58 (dd, *J* = 6.1, 1.9 Hz, 1H), 5.46 (dd, *J* = 6.2, 1.5 Hz, 1H), 4.84 (d, *J* = 1.9 Hz, 1H), 3.80 (s, 3H), 1.57 (s, 3H), 1.37 (s, 3H) ppm. **^{13}C NMR** (101 MHz, DMSO-d_6) δ 172.01, 168.15, 165.76, 155.88, 152.29, 148.73, 142.11, 140.40, 129.68, 124.37, 118.82, 112.86, 89.97, 86.42, 83.71, 83.35, 51.88, 26.61, 24.98 ppm. **HRMS** (ESI) Calculated for $C_{21}H_{23}O_6N_6$, $[M + H]^+$ m/z: 455.16736, found 455.16726.

(3a*S*,4*S*,6*R*,6a*R*)-6-(6-Amino-9*H*-purin-9-yl)-*N*-(4-carbamoylphenyl)-2,2-dimethyltetrahydrofuro[3,4-*d*][1,3]dioxol-4-carboxamide (**11b**)

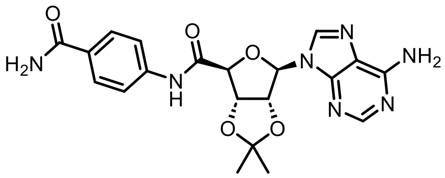

Obtained as a white solid (84 mg, yield 18 %, 80 % purity) which was used in the following step without further purification. **HRMS** (ESI) Calculated for $C_{20}H_{22}O_5N_7$, $[M + H]^+$ m/z: 440.16769, found 440.16738.

(3a*S*,4*S*,6*R*,6a*R*)-6-(6-Amino-9*H*-purin-9-yl)-*N*-(3-methoxyphenyl)-2,2-dimethyltetrahydrofuro[3,4-*d*][1,3]dioxole-4-carboxamide (**11c**)

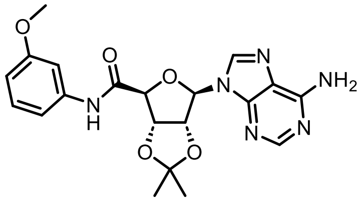

Obtained as an off-white solid (327 mg, yield 70 %).

^{1}H NMR (400 MHz, DMSO-*d₆*) δ 9.43 (s, 1H), 8.31 (s, 1H), 8.01 (s, 1H), 7.23 (s, 2H), 7.09 (t, *J* = 8.1 Hz, 1H), 6.90–6.82 (m, 2H), 6.58 (ddd, *J* = 8.3, 2.5, 1.0 Hz, 1H), 6.41 (d, *J* = 1.7 Hz, 1H), 5.52 (dd, *J* = 6.1, 2.0 Hz, 1H), 5.44 (dd, *J* = 6.1, 1.7 Hz, 1H), 4.77 (d, *J* = 2.0 Hz, 1H), 3.66 (s, 3H), 1.57 (s, 3H), 1.37 (s, 3H) ppm. **^{13}C NMR** (101 MHz, DMSO-d_6) δ 167.41, 159.11, 155.93, 152.41, 148.81, 140.18, 138.74, 129.05, 118.85, 112.97, 112.39, 109.58, 105.76, 89.81, 86.13, 83.40, 83.31, 54.90, 26.67, 25.02 ppm. **HRMS** (ESI) Calculated for $C_{20}H_{23}O_5N_6$, $[M + H]^+$ m/z: 427.17244, found 427.17227.

(3a*S*,4*S*,6*R*,6a*R*)-6-(6-Amino-9*H*-purin-9-yl)-*N*-(3-hydroxy-4-methoxyphenyl)-2,2-dimethyltetrahydrofuro[3,4-*d*][1,3]dioxole-4-carboxamide (**11d**)

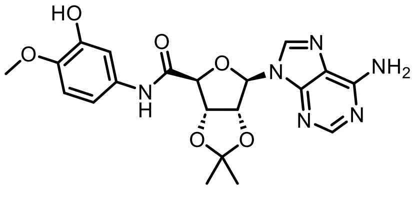

Obtained as a white solid (313 mg, yield 65 %).

^{1}H NMR (400 MHz, DMSO-d_6) δ 9.39 (s, 1H), 8.97 (s, 1H), 8.32 (s, 1H), 8.07 (s, 1H), 7.26 (s, 2H), 6.98 (d, *J* = 2.5 Hz, 1H), 6.74 (d, *J* = 8.7 Hz, 1H), 6.60 (dd, *J* = 8.7, 2.5 Hz, 1H), 6.37 (d, *J* = 1.9 Hz, 1H), 5.45–5.37 (m, 2H), 4.74 (d, *J* = 1.9 Hz, 1H), 3.69 (s, 3H), 1.57 (s, 3H), 1.36 (s, 3H) ppm. **^{13}C NMR** (101 MHz, DMSO-d_6) δ 166.76, 155.97, 152.52, 148.86, 146.10, 144.23, 139.98, 131.15, 118.85, 113.11, 112.03, 110.91, 108.61, 89.74, 85.78, 83.33, 83.20, 55.76, 26.76, 25.07 ppm. **HRMS** (ESI) Calculated for $C_{20}H_{23}O_6N_6$, $[M + H]^+$ m/z: 443.16736, found 443.16704.

(3a*S*,4*S*,6*R*,6a*R*)-6-(6-Amino-9*H*-purin-9-yl)-*N*-(3-hydroxy-4-methylphenyl)-2,2-dimethyltetrahydro furo[3,4-*d*][1,3]dioxole-4-carboxamide (**11e**)

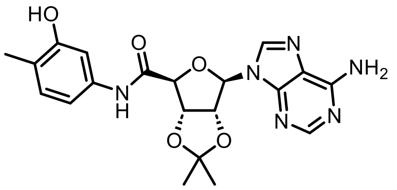

Obtained as an off-white solid (259 mg, yield 56 %).

^{1}H NMR (400 MHz, DMSO-*d$_6$*) δ 9.45 (s, 1H), 9.27 (s, 1H), 8.33 (s, 1H), 8.08 (s, 1H), 7.27 (s, 2H), 7.09 (d, *J* = 2.1 Hz, 1H), 6.89–6.83 (m, 1H), 6.51 (dd, *J* = 8.1, 2.1 Hz, 1H), 6.37 (d, *J* = 1.7 Hz, 1H), 5.45–5.37 (m, 2H), 4.75 (d, *J* = 1.7 Hz, 1H), 2.02 (s, 3H), 1.58 (s, 3H), 1.36 (s, 3H) ppm. **^{13}C NMR** (101 MHz, DMSO-*d$_6$*) δ 167.25, 156.28, 155.33, 152.88, 149.16, 140.25, 136.51, 130.28, 119.76, 119.16, 113.47, 110.89, 107.10, 90.08, 86.06, 83.63, 83.48, 27.08, 25.39, 15.79 ppm. **HRMS** (ESI) Calculated for $C_{20}H_{22}O_5N_6Na$, [*M* + Na]$^+$ m/z: 449.15439, found 449.15408.

Methyl 5-((3a*S*,4*S*,6*R*,6a*R*)-6-(6-amino-9*H*-purin-9-yl)-2,2-dimethyltetrahydrofuro[3,4-*d*][1,3] dioxole-4-carboxamido)-2-fluorobenzoate (**11f**)

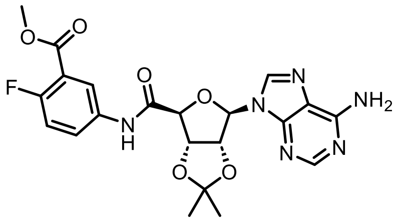

Obtained as a white solid (211 mg, yield 48 %).

^{1}H NMR (400 MHz, DMSO-*d*$_6$) δ 9.61 (s, 1H), 8.29 (s, 1H), 7.94 (s, 1H), 7.86 (dd, *J* = 6.6, 2.8 Hz, 1H), 7.42 (ddd, *J* = 9.0, 4.3, 2.8 Hz, 1H), 7.18 (s, 2H), 7.15 (m, 1H), 6.46 (d, *J* = 1.5 Hz, 1H), 5.58 (dd, *J* = 6.1, 1.9 Hz, 1H), 5.45 (dd, 1H), 4.80 (d, *J* = 1.9 Hz, 1H), 3.84 (s, 3H), 1.56 (s, 3H), 1.37 (s, 3H) ppm. **^{13}C NMR** (101 MHz, DMSO-*d*$_6$) δ 168.20, 163.92, 157.29 (d, *J* = 254.7 Hz), 156.19, 152.51, 148.98, 140.84, 134.24 (dd, *J* = 10.5, 3.0 Hz), 126.79 (m), 123.06 (d, *J* = 10.9 Hz), 119.15, 117.69 (d, *J* = 10.9 Hz), 117.07 (d, *J* = 23.4 Hz), 113.11, 90.30, 86.71, 84.06, 83.62, 52.66, 26.90, 25.27 ppm. **^{19}F NMR** (376 MHz, DMSO-*d*$_6$) δ -116.13 (ddd, *J* = 10.8, 6.7, 4.4 Hz) ppm. **HRMS** (ESI) Calculated for $C_{21}H_{22}O_6N_6F$, [*M* + H]$^+$ m/z: 473.15794, found 473.15808.

(3a*S*,4*S*,6*R*,6a*R*)-6-(6-Amino-9*H*-purin-9-yl)-*N*-(2,3-dihydrobenzo[*b*][1,4]dioxin-6-yl)-2,2-dimethyl tetrahydrofuro[3,4-*d*][1,3]dioxole-4-carboxamide (**11k**)

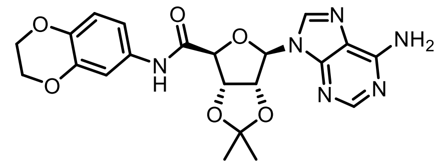

Obtained as a white solid (240 mg, yield 57 %).

^{1}H NMR (400 MHz, DMSO-*d*$_6$) δ 9.35 (s, 1H), 8.29 (s, 1H), 8.02 (s, 1H), 7.24 (s, 2H), 6.89 (s, 1H), 6.66 (s, 2H), 6.39 (s, 1H), 5.48 (m, 1H), 5.42 (m, 1H), 4.73 (s, 1H), 4.17 (s, 4H), 1.56 (s, 3H), 1.36 (s, 3H) ppm. **^{13}C NMR** (101 MHz, DMSO-*d*$_6$) δ 167.30, 156.25, 152.71, 149.13, 142.89, 140.41, 140.06, 131.49, 119.13, 116.64, 113.77, 113.29, 109.72, 90.04, 86.32, 83.63, 64.35, 64.16, 27.00, 25.34 ppm. **HRMS** (ESI) Calculated for $C_{21}H_{23}O_6N_6$, [*M* + H]$^+$ m/z: 455.16736, found 455.16726.

(3a*S*,4*S*,6*R*,6a*R*)-6-(6-Amino-9*H*-purin-9-yl)-2,2-dimethyl-*N*-(4-methyl-2-oxo-2*H*-chromen-7-yl) tetrahydrofuro[3,4-*d*][1,3]dioxole-4-carboxamide (**11l**)

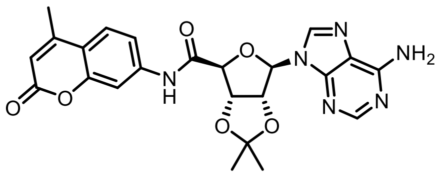

Obtained as a white solid (237 mg, yield 46 %).

^{1}H NMR (400 MHz, DMSO-*d*$_6$) δ 9.75 (s, 1H), 8.30 (s, 1H), 7.93 (s, 1H), 7.56 (d, *J* = 8.7 Hz, 1H), 7.43 (d, *J* = 2.1 Hz, 1H), 7.21 (dd, *J* = 8.7, 2.1 Hz, 1H), 7.15 (s, 2H), 6.48 (s, 1H), 6.24 (d, *J* = 1.4 Hz, 1H), 5.62 (dd, *J* = 6.1, 1.9 Hz, 1H), 5.46 (dd, *J* = 6.1, 1.4 Hz, 1H), 4.86 (d, *J* = 1.9 Hz, 1H), 2.36 (d, *J* = 1.3 Hz, 3H), 1.57 (s, 3H), 1.38 (s, 3H) ppm. **^{13}C NMR** (101 MHz, DMSO-*d*$_6$) δ 168.64, 160.25, 156.13, 153.41, 153.32, 152.47, 148.99, 141.38, 140.72, 125.58, 119.11, 116.08, 115.57, 113.09, 112.72, 106.60, 90.27, 86.85, 84.04, 83.68, 26.86, 25.23, 18.23 ppm. **HRMS** (ESI) Calculated for $C_{23}H_{23}O_6N_6$, [*M* + H]$^+$ m/z: 479.16736, found 479.16699.

(3a*S*,4*S*,6*R*,6a*R*)-6-(6-Amino-9*H*-purin-9-yl)-2,2-dimethyl-*N*-(2-oxo-4-(trifluoromethyl)-2*H*-chromen-7-yl)tetrahydrofuro[3,4-*d*][1,3]dioxole-4-carboxamide (**11m**)

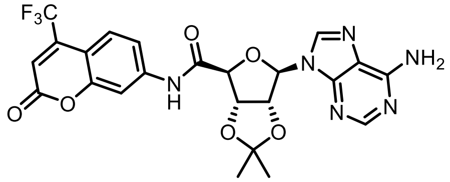

Obtained as a white solid (265 mg, yield 53 %).

^{1}H NMR (400 MHz, DMSO-d_6) δ 9.91 (s, 1H), 8.30 (s, 1H), 7.93 (s, 1H), 7.53 (m, 1H), 7.50 (m, 1H), 7.34 (dd, *J* = 8.9, 2.1 Hz, 1H), 7.13 (s, 2H), 6.88 (s, 1H), 6.49 (s, 1H), 5.64 (dd, *J* = 6.1, 1.9 Hz, 1H), 5.47 (m, 1H), 4.88 (d, *J* = 1.8 Hz, 1H), 1.57 (s, 3H), 1.38 (s, 3H) ppm. **^{13}C NMR** (101 MHz, DMSO-d_6) δ 169.02, 158.82, 156.12, 154.43, 152.46, 148.96, 142.41, 140.76, 139.38 (d, *J* = 32.2 Hz), 125.05, 123.27 (m), 119.09, 116.83, 114.93 (d, *J* = 5.6 Hz), 113.07, 108.78, 107.17, 90.30, 86.95, 84.10, 83.71, 26.83, 25.21 ppm. **^{19}F NMR** (376 MHz, DMSO-d_6) δ -63.58 ppm. **HRMS** (ESI) Calculated for $C_{23}H_{20}O_6N_6F_3$, [*M* + H]$^+$ m/z: 533.13909, found 533.13927.

(3a*S*,4*S*,6*R*,6a*R*)-6-(6-Amino-9*H*-purin-9-yl)-2,2-dimethyl-*N*-(4-methyl-2-oxo-1,2-dihydroquinolin-7-yl)tetrahydrofuro[3,4-*d*][1,3]dioxole-4-carboxamide (**11n**)

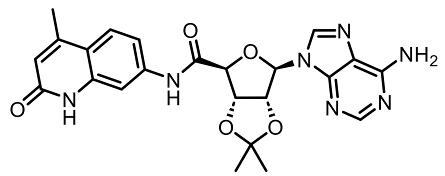

Obtained as a slightly yellow solid (425 mg, yield 95 %) of limited purity due to unfavorable physical properties, and used in the following step without further purification step. **UPLC-MS** (ESI) Calculated for $C_{23}H_{24}N_7O_5$, [*M* + H]$^+$ m/z: 478.18, found 478.26.

(3a*S*,4*S*,6*R*,6a*R*)-6-(6-Amino-9*H*-purin-9-yl)-2,2-dimethyl-*N*-(2-oxo-1,2,3,4-tetrahydro quinolin-7-yl)tetrahydrofuro[3,4-*d*][1,3]dioxol-4-carboxamide (**11o**)

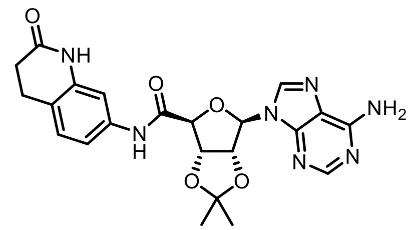

Obtained as a white solid (290 mg, yield 67 %).

^{1}H NMR (400 MHz, DMSO-*d$_6$*) δ 10.06 (s, 1H), 9.56 (s, 1H), 8.31 (s, 1H), 8.07 (s, 1H), 7.26 (s, 2H), 7.10 (d, *J* = 2.1 Hz, 1H), 6.97 (d, *J* = 8.2 Hz, 1H), 6.68 (dd, *J* = 8.1, 2.1 Hz, 1H), 6.39 (d, *J* = 1.9 Hz, 1H), 5.46 (dd, *J* = 6.1, 2.1 Hz, 1H), 5.40 (dd, *J* = 6.1, 1.9 Hz, 1H), 4.77 (d, *J* = 2.1 Hz, 1H), 2.76 (m, 2H), 2.39 (m, 2H), 1.57 (s, 3H), 1.36 (s, 3H) ppm. **^{13}C NMR** (101 MHz, DMSO-*d$_6$*) δ 170.24, 167.28, 156.08, 152.60, 148.84, 140.08, 138.22, 136.60, 127.47, 119.16, 118.87, 113.84, 113.12, 107.30, 89.82, 85.95, 83.40, 83.29, 30.52, 26.76, 25.09, 24.32 ppm. **HRMS** (ESI) Calculated for $C_{22}H_{24}O_5N_7$, $[M + H]^+$ m/z: 466.18334, found 466.18315.

(3a*S*,4*S*,6*R*,6a*R*)-6-(6-Amino-9*H*-purin-9-yl)-2,2-dimethyl-*N*-(quinolin-7-yl)tetrahydrofuro[3,4-*d*][1,3]dioxole-4-carboxamide (**11p**)

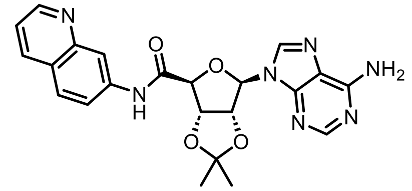

Obtained as an off-white solid (263 mg, yield 63 %).

^{1}H NMR (400 MHz, DMSO-*d$_6$*) δ 9.78 (s, 1H), 8.81 (dd, *J* = 4.2, 1.7 Hz, 1H), 8.34 (s, 1H), 8.25–8.19 (m, 1H), 8.19–8.15 (m, 1H), 7.97 (s, 1H), 7.76 (d, *J* = 8.8 Hz, 1H), 7.39 (q, *J* = 4.2 Hz, 1H), 7.34 (dd, *J* = 8.8, 2.1 Hz, 1H), 7.17 (s, 2H), 6.48 (d, *J* = 1.5 Hz, 1H), 5.62 (dd, *J* = 6.1, 2.0 Hz, 1H), 5.48 (dd, *J* = 6.0, 1.6 Hz, 1H), 4.89 (d, *J* = 1.9 Hz, 1H), 1.59 (s, 3H), 1.39 (s, 3H) ppm. **^{13}C NMR** (101 MHz, DMSO-*d*6) δ 168.36, 156.00, 152.55, 151.00, 148.98, 148.12, 140.73, 138.66, 135.82, 128.17, 124.89, 121.00, 120.53, 118.96, 117.42, 113.22, 90.21, 86.72, 83.86, 83.64, 26.83, 25.18 ppm. **HRMS** (ESI) Calculated for $C_{22}H_{22}O_4N_7$, $[M + H]^+$ m/z: 448.17278, found 448.17264.

(3a*S*,4*S*,6*R*,6a*R*)-6-(6-Amino-9*H*-purin-9-yl)-*N*-(4-iodophenyl)-2,2-dimethyltetrahydrofuro[3,4-*d*][1,3]dioxol-4-carboxamide (**S2**)

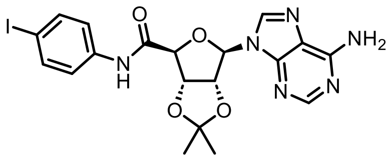

Obtained as a white solid (3.19 g, yield 65 %, starting from 3 g of **9**).

^{1}H NMR (400 MHz, DMSO-d_6) δ 9.52 (s, 1H), 8.29 (s, 1H), 7.97 (s, 1H), 7.51 (m, 2H), 7.22 (s, 2H), 7.09 (m, 2H), 6.43 (d, J = 1.6 Hz, 1H), 5.54 (dd, J = 6.0, 2.0 Hz, 1H), 5.44 (dd, J = 6.1, 1.6 Hz, 1H), 4.78 (d, J = 1.9 Hz, 1H), 1.56 (s, 3H), 1.36 (s, 3H) ppm. **^{13}C NMR** (101 MHz, DMSO-d_6) δ 167.66, 155.89, 152.32, 148.75, 140.30, 137.50, 136.89, 122.27, 118.82, 112.88, 89.85, 87.63, 86.30, 83.53, 83.33, 26.63, 25.09 ppm. **HRMS** (ESI) Calculated for $C_{19}H_{20}O_4N_6I$, $[M + H]^+$ m/z: 523.05852, found 523.05866.

General procedure B: Suzuki cross-coupling (**11g–11j**)

Iodo-derivative **S2** (300 mg, 0.57 mmol, 1 eq.) was mixed with Na_2CO_3 (116 mg, 1.09 mmol, 2 eq.) and a 10 ml mixture of 1,4-dioxane and water (4:1). The flask was backfilled with argon three times. Then, boronic acid or its ester (0.86 mmol, 1.5 eq.) and $Pd(dppf)Cl_2 \cdot CH_2Cl_2$ (24 mg, 0.029 mmol, 5 mol %) were added, and the backfilling with argon was repeated two more times. The reaction mixture was stirred at 100 °C, typically forming the product within 2 hours. After cooling, the mixture was transferred to an Erlenmeyer flask containing 150 mL of EtOAc acetate and Na_2SO_4. The drying agent was filtered off and the final product was isolated using C18 reverse-phase chromatography (H_2O/MeCN, 10 to 65 %).

(3a*S*,4*S*,6*R*,6a*R*)-6-(6-Amino-9*H*-purin-9-yl)-*N*-(4-(furan-3-yl)phenyl)-2,2-dimethyltetrahydrofuro[3,4-*d*][1,3]dioxol-4-carboxamide (**11g**)

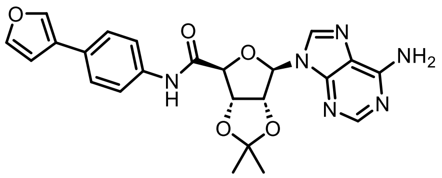

Obtained as a white solid (161 mg, yield 61 %).

^{1}H NMR (400 MHz, DMSO-d_6) δ 9.52 (s, 1H), 8.32 (s, 1H), 8.09 (dd, J = 1.6, 0.9 Hz, 1H), 8.02 (s, 1H), 7.70 (m, 1H), 7.44 (m, 2H), 7.28 (m, 2H), 7.23 (s, 2H), 6.89 (dd, J = 1.9, 0.9 Hz, 1H), 6.43 (d, J = 1.7 Hz, 1H), 5.53 (dd, J = 6.1, 2.1 Hz, 1H), 5.45 (dd, J = 6.1, 1.8 Hz, 1H), 4.80 (d, J = 2.0 Hz, 1H), 1.58 (s, 3H), 1.37 (s, 3H) ppm. **^{13}C NMR** (101 MHz, DMSO-d_6) δ 167.37, 155.93, 152.39, 148.82, 144.14, 140.22, 138.81, 136.48, 127.44, 125.43, 120.42, 118.86, 112.96, 108.57, 89.83, 86.15, 83.45, 83.34, 26.68, 25.02 ppm. **HRMS** (ESI) Calculated for $C_{23}H_{23}O_5N_6$, $[M + H]^+$ m/z: 463.17244, found 463.17237.

(3a*S*,4*S*,6*R*,6a*R*)-6-(6-Amino-9*H*-purin-9-yl)-2,2-dimethyl-*N*-(4-(thiophen-3-yl)phenyl)tetrahydrofuro[3,4-*d*][1,3]dioxol-4-carboxamide (**11h**)

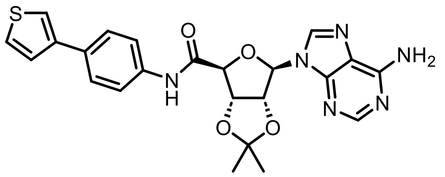

Obtained as a yellow solid (209 mg, 76 %).

^{1}H NMR (400 MHz, DMSO-d_6) δ 9.53 (s, 1H), 8.32 (s, 1H), 8.02 (s, 1H), 7.77 (m, 1H), 7.60 (dd, *J* = 5.0, 2.9 Hz, 1H), 7.55 (m, 2H), 7.50 (dd, *J* = 5.0, 1.4 Hz, 1H), 7.31 (m, 2H), 7.23 (s, 2H), 6.43 (d, *J* = 1.8 Hz, 1H), 5.54 (dd, *J* = 6.1, 2.1 Hz, 1H), 5.45 (dd, *J* = 6.1, 1.8 Hz, 1H), 4.80 (d, *J* = 2.0 Hz, 1H), 1.58 (s, 3H), 1.37 (s, 3H) ppm. **^{13}C NMR** (101 MHz, DMSO-d_6) δ 167.40, 155.94, 152.40, 148.83, 141.02, 140.21, 136.66, 130.69, 126.92, 125.95, 120.38, 120.06, 118.87, 113.96, 89.83, 86.16, 83.45, 83.33, 26.68, 25.02 ppm. **HRMS** (ESI) Calculated for $C_{23}H_{23}O_4N_6S$, $[M + H]^+$ m/z: 479.14960, found 479.14944.

(3a*S*,4*S*,6*R*,6a*R*)-*N*-(4-(1*H*-Pyrazol-4-yl)phenyl)-6-(6-amino-9*H*-purin-9-yl)-2,2-dimethyl tetrahydrofuro[3,4-*d*][1,3]dioxol-4-carboxamide (**11i**)

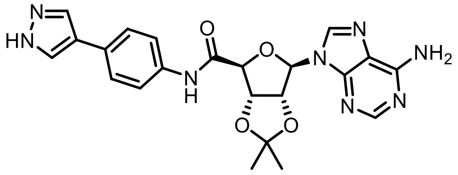

Obtained as a white solid (209 mg, 79 %).

^{1}H NMR (400 MHz, DMSO-d_6) δ 12.87 (s, 1H), 9.48 (s, 1H), 8.32 (s, 1H), 8.09 (s, 1H), 8.03 (s, 1H), 7.84 (s, 1H), 7.43 (m, 2H), 7.28–7.20 (m, 4H), 6.42 (d, *J* = 1.8 Hz, 1H), 5.52 (dd, *J* = 6.1, 2.1 Hz, 1H), 5.44 (dd, *J* = 6.1, 1.8 Hz, 1H), 4.79 (d, *J* = 2.0 Hz, 1H), 1.58 (s, 3H), 1.37 (s, 3H) ppm. **^{13}C NMR** (101 MHz, DMSO-d_6) δ 167.24, 155.95, 152.42, 148.84, 140.19, 135.51, 128.60, 124.95, 120.81, 120.55, 118.87, 113.08, 89.81, 86.09, 83.41, 83.33, 26.70, 25.03 ppm. **HRMS** (ESI) Calculated for $C_{22}H_{23}O_4N_8$, $[M + H]^+$ m/z: 463.18368, found 463.18363.

(3a*S*,4*S*,6*R*,6a*R*)-6-(6-Amino-9*H*-purin-9-yl)-*N*-(4-(3,5-dimethylisoxazol-4-yl)phenyl)-2,2-dimethyltetrahydrofuro[3,4-*d*][1,3]dioxol-4-carboxamide (**11j**)

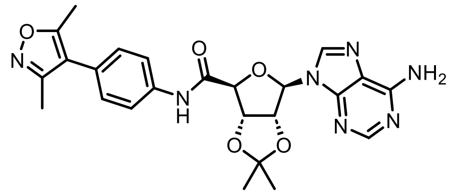

Obtained as a yellow solid (185 mg, 66 %).

^{1}H NMR (400 MHz, DMSO-d_6) δ 9.59 (s, 1H), 8.31 (s, 1H), 8.02 (s, 1H), 7.36 (m, 2H), 7.24–7.18 (m, 4H), 6.44 (d, *J* = 1.6 Hz, 1H), 5.55 (dd, *J* = 6.0, 2.0 Hz, 1H), 5.46 (dd, *J* = 6.0, 1.7 Hz, 1H), 4.81 (d, *J* = 2.0 Hz, 1H), 2.37 (s, 3H), 2.19 (s, 3H), 1.58 (s, 3H), 1.37 (s, 3H) ppm. **^{13}C NMR** (101 MHz, DMSO-d_6) δ 167.58, 164.79, 158.11, 155.93, 152.43, 148.83, 140.21, 136.97, 128.79, 125.11, 120.32, 117.85, 114.53, 110.96, 89.79, 86.15, 83.46, 83.32, 26.68, 25.03, 11.32, 10.48 ppm. **HRMS** (ESI) Calculated for $C_{24}H_{26}O_5N_7$, $[M + H]^+$ m/z: 492.19899, found 492.19911.

***General procedure C*:** synthesis of amides via T_3P activation (**16l**, **16n**, **16q**)

To an ice-cold solution of **15** (1 eq.) in dry THF (33 mL per 1 mmol) was added Et_3N (2 eq.) and a corresponding amine (2 eq.). Subsequently, T_3P (propanephosphonic anhydride, 50 % w/w solution in ethyl acetate, 1.5 eq.) was added dropwise. The reaction mixture was allowed to warm to room temperature and stirred for 2 to 24 h (UPLC-MS monitoring). Then, the reaction mixture was diluted with 150 mL of EtOAc and washed with brine. The organic phase was dried over Na_2SO_4, evaporated and purified using reverse-phase C18 flash chromatography (H_2O/MeCN, 50 to 100 %).

(2*S*,3*R*,4*R*,5*R*)-5-(4-Amino-5-iodo-7*H*-pyrrolo[2,3-*d*]pyrimidin-7-yl)-3,4-bis((tert-butyldimethylsilyl)oxy)-*N*-(4-methyl-2-oxo-2*H*-chromen-7-yl)tetrahydrofuran-2-carboxamide (**16l**)

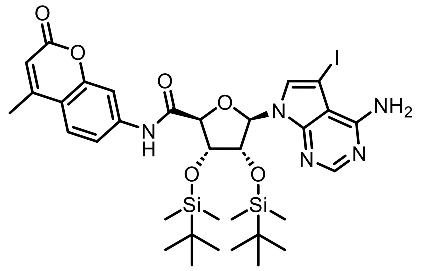

Obtained as an off-white solid (251 mg, yield 56 %; starting from 360 mg, 0.567 mmol of **15**).

^{1}H NMR (400 MHz, DMSO-*d*$_6$) δ 10.76 (s, 1H), 8.09 (s, 1H), 7.83–7.76 (m, 3H), 7.57 (dd, *J* = 8.6, 2.1 Hz, 1H), 6.75 (s, 2H), 6.32 (m, 1H), 6.14 (d, *J* = 6.1 Hz, 1H), 4.79 (dd, *J* = 6.0, 4.2 Hz, 1H), 4.59–4.51 (m, 2H), 2.42 (d, *J* = 1.3 Hz, 3H), 0.91 (s, 9H), 0.74 (s, 9H), 0.13 (s, 3H), 0.11 (s, 3H), -0.09 (s, 3H), -0.34 (s, 3H) ppm. **^{13}C NMR** (101 MHz, DMSO-*d*$_6$) δ 169.09, 160.61, 157.59, 153.81, 153.75, 152.43, 150.46, 141.46, 128.40, 126.47, 116.62, 116.35, 113.13, 107.28, 104.06, 88.45, 84.49, 74.94, 74.78, 52.46, 26.01, 25.83, 18.40, 18.14, 17.91, -4.37, -4.44, -4.62, -5.37 ppm. **HRMS** (ESI) Calculated for $C_{33}H_{47}O_6N_5ISi_2$, [*M* + H]$^+$ m/z: 792.21041, found 792.21022.

(2*S*,3*R*,4*R*,5*R*)-5-(4-Amino-5-iodo-7*H*-pyrrolo[2,3-*d*]pyrimidin-7-yl)-3,4-bis((tert-butyldimethylsilyl)oxy)-*N*-(4-methyl-2-oxo-1,2-dihydroquinolin-7-yl)tetrahydrofuran-2-carboxamide (16n)

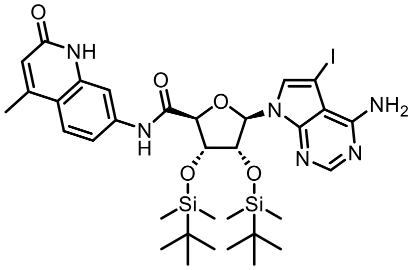

Obtained as a slightly yellow solid (249 mg, yield 76 %; starting from 264 mg, 0.416 mmol of **15**).

^{1}H NMR (400 MHz, DMSO-d_6) δ 11.58 (s, 1H), 10.70 (s, 1H), 8.12 (s, 1H), 7.92 (q, J = 2.2 Hz, 1H), 7.84 (s, 1H), 7.70 (d, J = 8.7 Hz, 1H), 7.33 (dd, J = 8.8, 2.1 Hz, 1H), 6.75 (br s, 2H), 6.30 (d, J = 1.6 Hz, 1H), 6.13 (d, J = 6.0 Hz, 1H), 4.77 (dd, J = 6.1, 4.3 Hz, 1H), 4.56 (d, J = 2.6 Hz, 1H), 4.52 (dd, J = 4.3, 2.7 Hz, 1H), 2.42–2.37 (m, 3H), 0.92 (s, 9H), 0.74 (s, 9H), 0.14 (s, 3H), 0.11 (s, 3H), -0.09 (s, 3H), -0.34 (s, 3H) ppm. **^{13}C NMR** (101 MHz, DMSO-d_6) δ 168.35, 161.91, 157.32, 152.18, 150.00, 147.58, 139.61, 139.43, 127.86, 125.43, 119.54, 116.26, 114.26, 105.60, 103.65, 88.11, 84.10, 74.62, 74.55, 52.13, 25.68, 25.50, 18.39, 17.79, 17.57, -4.71, -4.80, -4.97, -5.71 ppm. **HRMS** (ESI) Calculated for $C_{33}H_{48}O_5N_6ISi_2$, $[M + H]^+$ m/z: 791.22639, found 791.22684.

(2*S*,3*R*,4*R*,5*R*)-5-(4-Amino-5-iodo-7H-pyrrolo[2,3-*d*]pyrimidin-7-yl)-3,4-bis((tert-butyldimethylsilyl)oxy)-*N*-methyl-*N*-(4-methyl-2-oxo-1,2-dihydroquinolin-7-yl)tetrahydrofuran-2-carboxamide (16q)

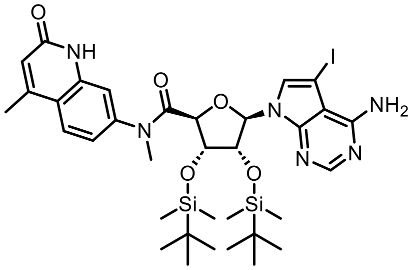

Obtained as a slightly yellow solid (200 mg, yield 42 %; starting from 375 mg, 0.591 mmol of **15**).

^{1}H NMR (400 MHz, DMSO-d_6) δ 11.59 (s, 1H), 10.73 (s, 1H), 9.17 (s, 1H), 9.06 (s, 2H), 8.18 (s, 1H), 8.16 (s, 1H), 7.89 (d, J = 2.1 Hz, 1H), 7.70 (d, J = 8.7 Hz, 1H), 7.36 (dd, J = 8.7, 2.1 Hz, 1H), 7.06–6.78 (bs, 2H), 6.30 (d, J = 1.5 Hz, 1H), 6.20 (d, J = 5.7 Hz, 1H), 4.82 (dd, J = 5.7, 4.3 Hz, 1H), 4.62–4.58 (m, 1H), 4.58–4.53 (m, 1H), 2.39 (d, J = 1.2 Hz, 3H), 0.92 (s, 9H), 0.77 (s, 9H), 0.13 (s, 3H), 0.12 (s, 3H), -0.06 (s, 3H), -0.28 (d, J = 0.9 Hz, 3H) ppm. **^{13}C NMR** (101 MHz, DMSO-d_6) δ 169.41, 162.36, 157.55, 152.72, 151.33, 148.29, 139.90, 127.17, 121.60, 121.06, 113.95, 103.42, 85.76, 81.23, 76.76, 75.10, 53.23, 38.03, 25.81, 25.79, 18.90, 17.99, -4.26, -4.49, -5.16 ppm. **HRMS** (ESI) Calculated for $C_{34}H_{50}O_5N_6ISi_2$, $[M + H]^+$ m/z: 805.24204, found 805.24180.

General procedure D: Sonogashira cross-coupling[8] (**17l**, **17n**, **17q**)

A mixture of intermediate **16** (1 eq.), 5-ethynylpyrimidine (2 eq.), and Et_3N (3 eq.) in dry THF (15 mL per 1 mmol) was degassed and the flask was backfilled with argon three times. Then, CuI (0.3 eq.) and $Pd(PPh_3)_2Cl_2$ (0.1 eq.) were added, and the backfilling with argon was repeated two more times. The reaction mixture was then heated at 60 °C for 2 hours. After reaction completion the reaction mixture was diluted with 150 mL of EtOAc and washed with brine. The organic layer was dried over anhydrous Na_2SO_4, evaporated and purified using reverse-phase C18 flash chromatography (H_2O/MeCN, 50 to 100 %).

(2*S*,3*R*,4*R*,5*R*)-5-(4-Amino-5-(pyrimidin-5-ylethynyl)-7*H*-pyrrolo[2,3-*d*]pyrimidin-7-yl)-3,4-bis((tert-butyldimethylsilyl)oxy)-*N*-(4-methyl-2-oxo-2*H*-chromen-7-yl)tetrahydrofuran-2-carboxamide (**17l**)

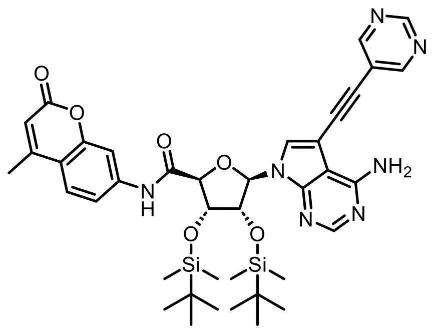

Obtained as an off-white solid (203 mg, yield 88 %; starting from 235 mg, 0.297 mmol of **16l**).

^{1}H NMR (400 MHz, DMSO-d_6) δ 10.80 (s, 1H), 9.15 (s, 1H), 9.05 (s, 2H), 8.15 (s, 1H), 8.13 (s, 1H), 7.84–7.75 (bs, 2H), 7.57 (dd, *J* = 8.7, 2.1 Hz, 1H), 7.09–6.70 (m, 2H), 6.31 (d, *J* = 1.3 Hz, 1H), 6.20 (d, *J* = 5.6 Hz, 1H), 4.83 (m, 1H), 4.61–4.55 (m, 2H), 2.41 (d, *J* = 1.3 Hz, 3H), 0.91 (s, 9H), 0.76 (s, 9H), 0.12 (s, 3H), 0.11 (s, 3H), -0.06 (s, 3H), -0.28 (s, 3H) ppm. **^{13}C NMR** (101 MHz, DMSO-d_6) δ 168.73, 160.00, 158.66, 157.60, 156.47, 153.56, 153.13, 153.10, 150.08, 141.25, 128.64, 126.11, 119.32, 116.13, 115.95, 112.87, 106.87, 102.22, 94.42, 89.22, 88.64, 85.07, 83.94, 74.80, 74.68, 29.03, 25.72, 25.60, 18.06, 17.84, 17.65, -4.62, -4.74, -4.97, -5.42 ppm. **HRMS** (ESI) Calculated for $C_{39}H_{50}O_6N_7Si_2$, $[M + H]^+$ m/z: 768.33556, found 768.33496.

(2*S*,3*R*,4*R*,5*R*)-5-(4-Amino-5-(pyrimidin-5-ylethynyl)-7*H*-pyrrolo[2,3-*d*]pyrimidin-7-yl)-3,4-bis((tert-butyldimethylsilyl)oxy)-*N*-(4-methyl-2-oxo-1,2-dihydroquinolin-7-yl)tetrahydrofuran-2-carboxamide (**17n**)

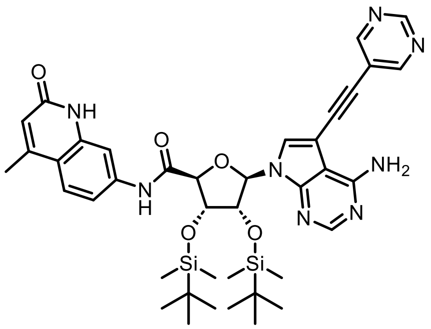

Obtained as a slightly yellow solid (212 mg, yield 95 %; starting from 230 mg, 0.291 mmol of **16n**).

^{1}H NMR (400 MHz, DMSO-d_6) δ 11.59 (s, 1H), 10.73 (s, 1H), 9.17 (s, 1H), 9.06 (s, 2H), 8.18 (s, 1H), 8.16 (s, 1H), 7.89 (q, *J* = 2.1 Hz, 1H), 7.70 (d, *J* = 8.7 Hz, 1H), 7.36 (dd, *J* = 8.7, 2.1 Hz, 1H), 7.12–6.75 (bs, 2H), 6.33–6.28 (m, 1H), 6.20 (d, *J* = 5.6 Hz, 1H), 4.82 (dd, *J* = 5.7, 4.3 Hz, 1H), 4.60 (dd, *J* = 3.1, 0.8 Hz, 1H), 4.58– 4.53 (m, 1H), 2.39 (d, *J* = 1.2 Hz, 3H), 0.92 (s, 9H), 0.77 (s, 9H), 0.13 (s, 3H), 0.12 (s, 3H), -0.06 (s, 3H), -0.27 (s, 3H) ppm. **^{13}C NMR** (101 MHz, DMSO-d_6) δ 168.40, 157.58, 156.58, 158.63, 153.15, 149.93, 147.58, 139.39, 139.29, 139.29, 125.43, 119.55, 119.27, 116.31, 114.26, 111.23, 105.62, 102.10, 94.22, 89.17, 88.53, 84.92, 84.02, 74.90, 74.61, 28.55, 25.68, 25.53, 18.40, 17.79, 17.59, -4.68, -4.81, -5.00, -5.51 ppm. **HRMS** (ESI) Calculated for $C_{39}H_{51}O_5N_8Si_2$, [*M* + H]$^+$ m/z: 767.35155, found 767.35208.

(2*S*,3*R*,4*R*,5*R*)-5-(4-Amino-5-(pyrimidin-5-ylethynyl)-7*H*-pyrrolo[2,3-*d*]pyrimidin-7-yl)-3,4-bis((tert-butyldimethylsilyl)oxy)-*N*-methyl-*N*-(4-methyl-2-oxo-1,2-dihydroquinolin-7-yl)tetrahydrofuran-2-carboxamide (**17q**)

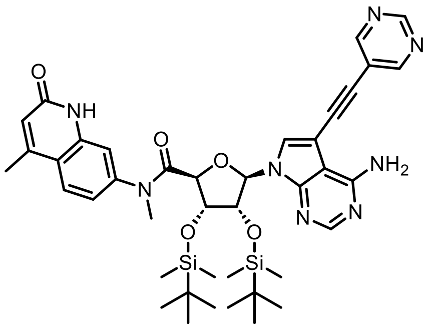

Obtained as a slightly yellow solid (165 mg, yield 89 %; starting from 192 mg, 0.239 mmol of **16q**).

^{1}H NMR (400 MHz, DMSO) δ 11.74 (s, 1H), 9.17 (s, 1H), 9.11 (s, 2H), 8.22 (s, 1H), 8.17 (s, 1H), 7.81 (d, *J* = 8.5 Hz, 1H), 7.25–7.16 (m, 2H), 7.02–6.75 (bs, 2H), 6.44 (m, 1H), 6.24 (d, *J* = 6.8 Hz, 1H), 4.84 (s, 1H), 4.44–4.35 (m, 2H), 2.42 (d, *J* = 1.2 Hz, 3H), 0.67 (s, 9H), 0.64 (s, 9H), 0.02 (s, 3H), -0.16 (s, 3H), -0.24 (s, 3H), -0.30 (s, 3H) ppm. **HRMS** (ESI) Calculated for $C_{40}H_{53}O_5N_8Si_2$, [*M* + H]$^+$ m/z: 781.36720, found 781.36762.

***General procedure E*:** synthesis of amides and sulfonamides (**21a**, **21k**, **22k**)

To an ice-cooled solution of amine **20** (300 mg, 0.98 mmol) and Et_3N (200 µL, 1.47 mmol, 1.5 eq.) in dry dichloromethane (30 mL) sulfonyl/acyl chloride (0.98 mmol, 1 eq.) was added dropwise. After stirring for 2 hours at 0 °C the solution was diluted with DCM, washed with brine and the organic phase was dried over Na_2SO_4, evaporated, and used without further purification in the next step, as outlined in *General procedure F*.

***General procedure F*:** removal of protecting groups (**12a–12p**, **18l**, **18n**, **18q**)

Protected nucleoside (**11a–11p**, **17l**, **17n**, **17q**, products of *General procedure E*) was dissolved in 80% formic acid (aqueous solution, 2 ml per 1 mmol of nucleoside) and the reaction mixture was stirred at room temperature for 16 hours or 36 hours in case of **18l**, **18n**, and **18q**. Solvent was removed in vacuo and the product was purified using reverse-phase C18 flash chromatography (H_2O/MeCN, 2 to 40 %).

Methyl 4-((2*S*,3*S*,4*R*,5*R*)-5-(6-amino-9*H*-purin-9-yl)-3,4-dihydroxytetrahydrofuran-2-carboxamido)benzoate (**12a**)

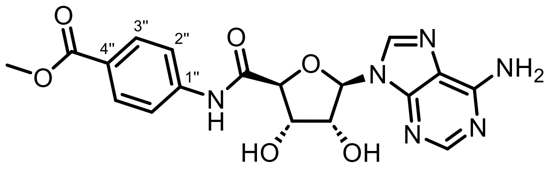

Compound **12a** (67 mg, yield 67 %) was prepared from **11a** (110 mg, 0,24 mmol) according to *General procedure F* and obtained as a white solid.

^{1}H NMR (400 MHz, DMSO-d_6) δ 10.69 (s, 1H, CO**NH**), 8.47 (s, 1H, H8), 8.15 (s, 1H, H2), 7.98 (m, 2H, H3''), 7.79 (m, 2H, H2''), 7.41 (s, 2H, NH_2), 6.06 (d, $J_{1'-2'}$ = 7.0 Hz, 1H, H1'), 5.88 (bs, 1H, 3'OH), 5.68 (bs, 1H, 2'OH), 4.68 (m, 1H, H2'), 4.58 (d, $J_{4'-3'}$ = 2.0 Hz, 1H, H4'), 4.37 (m, 1H, H3'), 3.84 (s, 3H, CO**OMe**) ppm.

^{13}C NMR (101 MHz, DMSO-d_6) δ 168.87 (**CO**NH), 165.75 (**CO**OMe), 156.21 (C6), 152.66 (C2), 149.16 (C4), 142.35 (C1''), 140.17 (C8), 130.25 (C3''), 124.83 (C4''), 119.68 (C2''), 119.45 (C5), 87.85 (C1'), 84.28 (C4'), 73.18 (C3'), 72.58 (C2'), 51.94 (CO**OMe**) ppm.

HRMS (ESI) Calculated for $C_{18}H_{19}O_6N_6$, $[M + H]^+$ m/z: 415.13606, found 415.13599.

$[\alpha]_D^{20}$ -57.9 (*c* 0.247, DMSO).

Melting point: 169.8-171.7 °C.

(2*S*,3*S*,4*R*,5*R*)-5-(6-Amino-9*H*-purin-9-yl)-*N*-(4-carbamoylphenyl)-3,4-dihydroxytetrahydrofuran-2-carboxamide (**12b**)

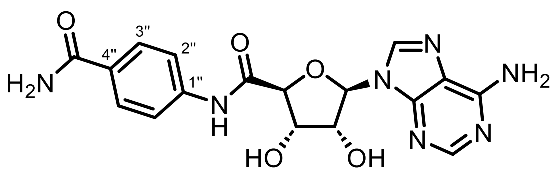

Compound **12b** (32 mg, yield 59 %) was prepared from **11b** (60 mg, 0.137 mmol) according to *General procedure F* and obtained as a white solid.

^{1}H NMR (400 MHz, DMSO-d_6) δ 10.59 (s, 1H, CO**NH**), 8.46 (s, 1H, H8), 8.14 (s, 1H, H2), 7.86–7.92 (m, 3H, H3'', 4''-CO$\mathbf{NH_A}$), 7.69 (m, 2H, H2''), 7.42 (s, 2H, NH_2), 7.28 (s, 1H, CO$\mathbf{NH_B}$), 6.06 (d, $J_{1'-2'}$ = 6.9 Hz, 1H, H1'), 5.84 (d, $J_{OH-3'}$ = 4.6 Hz, 1H, 3'OH), 5.66 (d, $J_{OH-2'}$ = 6.2 Hz, 1H, 2'OH), 4.68 (m, 1H, H2'), 4.56 (d, $J_{4'-3'}$ = 2.2 Hz, 1H, H4'), 4.36 (m, 1H, H3') ppm.

^{13}C NMR (101 MHz, DMSO-d_6) δ 168.58 (**CO**NH), 167.27 (4''-**CO**NH_2), 156.22 (C6), 152.59 (C2), 149.12 (C4), 140.51 (C1"), 140.21 (C8), 129.70 (C4''), 128.34 (C3''), 119.47 (C5), 119.41 (C2"), 87.84 (C1'), 84.33 (C4'), 73.16 (C3'), 72.52 (C2') ppm.

HRMS (ESI) Calculated for $C_{17}H_{18}O_5N_7$, [*M* + H]$^+$ m/z: 400.13639, found 400.13611.

$[\alpha]_D^{20}$ -38.2 (*c* 0.191, DMSO).

Melting point: 280.2 °C, decomposition.

(2*S*,3*S*,4*R*,5*R*)-5-(6-Amino-9*H*-purin-9-yl)-3,4-dihydroxy-*N*-(3-methoxyphenyl)tetrahydrofuran-2-carboxamide (**12c**)

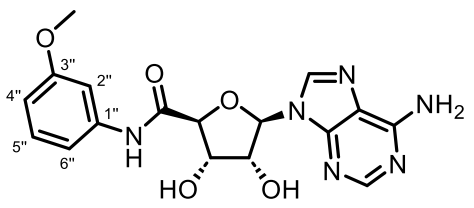

Compound **12c** (201 mg, yield 77 %) was prepared from **11c** (290 mg, 0.680 mmol) according to *General procedure F* and obtained as a white solid.

^{1}H NMR (400 MHz, DMSO-d_6) δ 10.41 (s, 1H, CO**NH**), 8.46 (s, 1H, H8), 8.14 (s, 1H, H2), 7.42 (s, 2H, NH_2), 7.32–7.24 (m, 2H, H2′′, H5′′), 7.18 (dm, $J_{6''-5''}$ = 8.1, 1H, H6′′), 6.72 (ddd, $J_{4''-5''}$ = 8.5, $J_{4''-2''}$ = 2.5, $J_{4''-6''}$ = 0.9 Hz, 1H, H4′′), 6.05 (d, $J_{1'-2'}$ = 7.0 Hz, 1H, H1′), 5.82 (d, $J_{OH-3'}$ = 4.6 Hz, 1H, 3′OH), 5.64 (d, $J_{OH-2'}$ = 6.2 Hz, 1H, 2′OH), 4.68 (m, 1H, H2′), 4.52 (d, $J_{4'-3'}$ = 2.2 Hz, 1H, H4′), 4.33 (m, 1H, H3′), 3.75 (s, 3H, 3′′-**OMe**) ppm.

^{13}C NMR (101 MHz, DMSO-d_6) δ 168.27 (**CO**NH), 159.49 (C3′′), 156.24 (C6), 152.56 (C2), 149.10 (C4), 140.28 (C8), 139.09 (C1′′), 129.61 (C5′′), 119.49 (C5), 112.48 (C6′′), 109.65 (C4′′), 106.07 (C2′′), 87.85 (C1′), 84.43 (C4′), 73.16 (C3′), 72.49 (C2′), 55.04 (3′′-**OMe**) ppm.

HRMS (ESI) Calculated for $C_{17}H_{19}O_5N_6$, $[M + H]^+$ m/z: 387.14114, found 387.14096.

$[\alpha]_D^{20}$ -25.3 (*c* 0.300, DMSO).

Melting point: 235.8-237.4 °C.

(2*S*,3*S*,4*R*,5*R*)-5-(6-Amino-9*H*-purin-9-yl)-3,4-dihydroxy-*N*-(3-hydroxy-4-methoxyphenyl)tetrahydrofuran-2-carboxamide (**12d**)

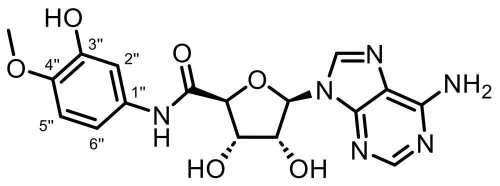

Compound **12d** (179 mg, yield 68 %) was prepared from **11d** (289 mg, 0.653 mmol) according to *General procedure F* and obtained as a white solid.

^{1}H NMR (400 MHz, DMSO-*d₆*) δ 10.31 (s, 1H, CO**NH**), 9.15 (s, 1H, 3′′OH), 8.46 (s, 1H, H8), 8.16 (s, 1H, H2), 7.44 (s, 2H, NH_2), 7.19 (d, $J_{2''-6''}$ = 2.5 Hz, 1H, H2′′), 6.98 (dd, $J_{6''-5''}$ = 8.7, $J_{6''-2''}$ = 2.5 Hz, 1H, H6′′), 6.90 (d, $J_{5''-6''}$ = 8.7 Hz, 1H, H5′′), 6.03 (d, $J_{1'-2'}$ = 7.1 Hz, 1H, H1′), 5.81 (d, $J_{OH-3'}$ = 4.5 Hz, 1H, 3′OH), 5.63 (d, $J_{OH-2'}$ = 6.3 Hz, 1H, 2′OH), 4.65 (m, 1H, H2′), 4.49 (d, $J_{4'-3'}$ = 2.0 Hz, 1H, H4′), 4.28 (m, 1H, H3′), 3.75 (s, 3H, 4′′**-OMe**) ppm.

^{13}C NMR (101 MHz, DMSO-*d₆*) δ 167.69 (**CO**NH), 156.25 (C6), 152.62 (C2), 148.99 (C4), 146.47 (C3′′), 144.46 (C4′′), 140.35 (C8), 131.39 (C1′′), 119.51 (C5), 112.45 (C5′′), 111.07 (C6′′), 108.65 (C2′′), 87.87 (C1′), 84.58 (C4′), 73.18 (C3′), 72.49 (C2′), 55.84 (4′′**-OMe**) ppm.

HRMS (ESI-) Calculated for $C_{17}H_{17}O_6N_6$, [*M* - H]$^-$ m/z: 401.12151, found 401.12099.

$[\alpha]_D^{20}$ -39.1 (c 0.312, DMSO).

Melting point: 198.3-199.7 °C.

(2*S*,3*S*,4*R*,5*R*)-5-(6-Amino-9*H*-purin-9-yl)-3,4-dihydroxy-*N*-(3-hydroxy-4-methylphenyl)tetrahydrofuran-2-carboxamide (**12e**)

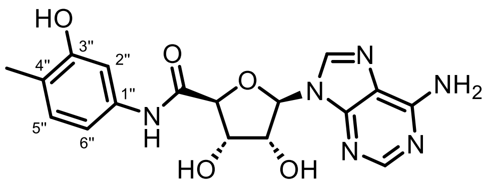

Compound **12e** (131 mg, yield 63 %) was prepared from **11e** (230 mg, 0.539 mmol) according to *General procedure F* and obtained as a white solid

^{1}H NMR (400 MHz, DMSO-d_6) δ 10.34 (s, 1H, CO**NH**), 9.43 (s, 1H, 3′′OH), 8.46 (s, 1H, H8), 8.18 (s, 1H, H2), 7.44 (s, 2H, NH_2), 7.24 (d, $J_{2''-6''}$ = 2.0 Hz, 1H, H2′′), 7.02 (d, $J_{5''-6''}$ = 8.2 Hz, 1H, H5′′), 6.92 (dd, $J_{6''-5''}$ = 8.2, $J_{6''-2''}$ = 2.0 Hz, 1H, H6′′), 6.04 (d, $J_{1'-2'}$ = 7.1 Hz, 1H, H1′), 5.82 (d, $J_{OH-3'}$ = 4.5 Hz, 1H, 3′OH), 5.63 (d, $J_{OH-2'}$ = 6.3 Hz, 1H, 2′OH), 4.64 (m, 1H, H2′), 4.51 (d, $J_{4'-3'}$ = 2.1 Hz, 1H, H4′), 4.28 (m, 1H, H3′), 2.09 (s, 3H, 4′′-**Me**) ppm.

^{13}C NMR (101 MHz, DMSO-d_6) δ 167.91 (**CO**NH), 156.25 (C6), 155.34 (C3′′), 152.71 (C2), 148.99 (C4), 140.32 (C8), 136.47 (C1′′), 130.34 (C5′′), 119.77 (C4”), 119.51 (C5), 110.85 (C6′′), 106.86 (C2′′), 87.90 (C1′), 84.57 (C4′), 73.20 (C3′), 72.51 (C2′), 15.52 (4′′-**Me**) ppm.

HRMS (ESI) Calculated for $C_{17}H_{17}O_5N_6$, [*M* - H]$^-$ m/z: 385.12659, found 385.12617.

[α]$_D^{20}$ -24.1 (c 0.299, DMSO).

Melting point: 179.7-181.3 °C.

Methyl 5-((2*S*,3*S*,4*R*,5*R*)-5-(6-amino-9*H*-purin-9-yl)-3,4-dihydroxytetrahydrofuran-2-carboxamido)-2-fluorobenzoate (12f)

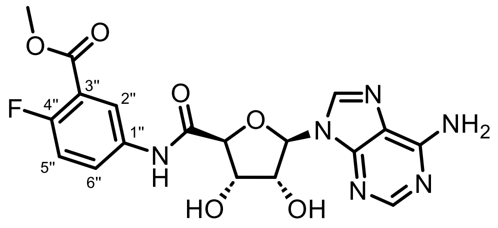

Compound **12f** (56 mg, yield 31 %) was prepared from **11f** (200 mg, 0.423 mmol) according to *General procedure F* and obtained as a white solid.

^{1}H NMR (400 MHz, DMSO-*d₆*) δ 10.67 (s, 1H, CO**NH**), 8.44 (s, 1H, H8), 8.20 (dd, $J_{2''\text{-F}}$ = 6.6, $J_{2''\text{-}6''}$ = 2.8 Hz, 1H, H2''), 8.16 (s, 1H, H2), 7.90 (ddd, $J_{6''\text{-}5''}$ = 9.1, $J_{6''\text{-F}}$ = 4.2, $J_{6''\text{-}2''}$ = 2.8 Hz, 1H, H6''), 7.43 (s, 2H, NH_2), 7.37 (dd, $J_{5''\text{-F}''}$ = 10.5, $J_{5''\text{-}6''}$ = 9.1 Hz, 1H, H5''), 6.05 (d, $J_{1'\text{-}2'}$ = 7.2 Hz, 1H, H1'), 5.86 (d, $J_{OH\text{-}3'}$ = 4.5 Hz, 1H, 3'OH), 5.64 (d, $J_{OH\text{-}2'}$ = 6.3 Hz, 1H, 2'OH), 4.70 (m, 1H, H2'), 4.53 (d, $J_{4'\text{-}3'}$ = 2.0 Hz, 1H, H4'), 4.35 (m, 1H, H3'), 3.88 (s, 3H, CO**OMe**) ppm.

^{13}C NMR (101 MHz, DMSO-*d₆*) δ 168.50 (**CO**NH), 163.72 (d, $J_{CO\text{-F}}$ = 3.9 Hz, **CO**OMe), 157.20 (d, $J_{4''\text{-F}}$ = 254.9 Hz, H4''), 156.26 (C6), 152.57 (C2), 149.05 (C4), 140.41 (C8), 134.31 (d, $J_{1''\text{-F}}$ = 3.0 Hz, C1''), 126.83 (d, $J_{6''\text{-F}}$ = 8.8 Hz, C6''), 123.02 (C2''), 119.56 (C5), 117.91 (d, $J_{3''\text{-F}}$ = 11.4 Hz, C3''), 117.51 (d, $J_{5''\text{-F}}$ = 23.3 Hz, H5''), 87.89 (C1'), 84.37 (C4'), 73.14 (C3'), 72.28 (C2'), 52.46 (CO**OMe**) ppm.

^{19}F NMR (376 MHz, DMSO-*d₆*) δ -115.53 (ddd, $J_{5''\text{-F}''}$ =10.5, $J_{F\text{-}2''}$ = 6.5, $J_{F\text{-}6''}$ = 4.2 Hz) ppm.

HRMS (ESI) Calculated for $C_{18}H_{18}O_6N_6F$, [*M* + H]$^+$ m/z: 433.12664, found 433.12643.

$[\alpha]_D^{20}$ -20.0 (c 0.284, DMSO).

Melting point: 260.0 °C, decomposition.

(2*S*,3*S*,4*R*,5*R*)-5-(6-Amino-9*H*-purin-9-yl)-*N*-(4-(furan-3-yl)phenyl)-3,4-dihydroxytetrahydrofuran-2-carboxamide (**12g**)

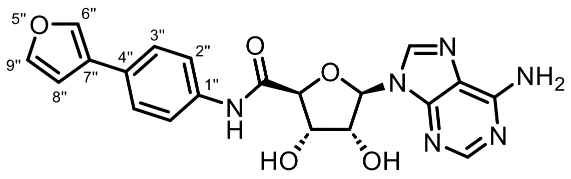

Compound **12g** (50 mg, yield 50 %) was prepared from **11g** (110 mg, 0.238 mmol) according to *General procedure F* and obtained as a white solid.

^{1}H NMR (400 MHz, DMSO-d_6) δ 10.51 (s, 1H, CO**NH**), 8.47 (s, 1H, H8), 8.16–8.14 (m, 2H, H2, H9′′), 7.73 (dd, $J_{9''-7''}$ = 1.8, $J_{9''-6''}$ = 1.8 Hz, 1H, H6′′), 7.68–7.58 (m, 4H, H2′′, H3′′), 7.44 (s, 2H, NH_2), 6.95 (dd, $J_{7''-9''}$ = 1.8, $J_{7''-6''}$ = 0.8 Hz, 1H, H8′′), 6.06 (d, $J_{1'-2'}$ = 7.1 Hz, 1H, H1′), 5.85 (d, $J_{OH-3'}$ = 4.5 Hz, 1H, 3′OH), 5.66 (d, $J_{OH-2'}$ = 6.2 Hz, 1H, 2′OH), 4.69 (m, 1H, H2′), 4.54 (d, $J_{4'-3'}$ = 2.1 Hz, 1H, H4′), 4.34 (m, 1H, H3′) ppm.

^{13}C NMR (101 MHz, DMSO-d_6) δ 168.22 (**CO**NH), 156.27 (C6), 152.60 (C2), 149.09 (C4), 144.24 (C6′′), 140.34 (C8), 138.93 (C9′′), 136.73 (C1′′), 127.86 (C4′′), 125.92 (C3′′), 125.42 (C7′′), 120.62 (C2′′), 119.53 (C5), 108.61 (C8′′), 87.89 (C1′), 84.48 (C4′), 73.19 (C3′), 72.50 (C2′) ppm.

HRMS (ESI) Calculated for $C_{20}H_{19}O_5N_6$, $[M + H]^+$ m/z: 423.14114, found 423.14090.

$[\alpha]_D^{20}$ -67.7 (*c* 0.274, DMSO).

Melting point: 213.3 °C, decomposition.

(2*S*,3*S*,4*R*,5*R*)-5-(6-Amino-9*H*-purin-9-yl)-3,4-dihydroxy-*N*-(4-(thiophen-3-yl)phenyl)tetrahydrofuran-2-carboxamide (**12h**)

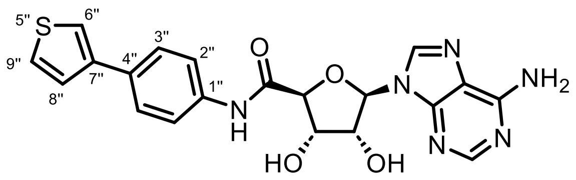

Compound **12h** (109 mg, yield 63 %) was prepared from **11h** (190 mg, 0.397 mmol) according to *General procedure F* and obtained as a slightly yellow solid.

^{1}H NMR (400 MHz, DMSO-d_6) δ 10.54 (s, 1H, CO**NH**), 8.47 (s, 1H, C8), 8.16 (s, 1H, C2), 7.83 (dd, $J_{6''-9''}$ = 2.9, $J_{6''-8''}$ = 1.3 Hz, 1H, C6''), 7.78–7.71 (m, 2H, H3''), 7.71–7.64 (m, 2H, H2''), 7.63 (dd, $J_{9''-8''}$ = 5.0, $J_{9''-6''}$ = 2.9 Hz, 1H, H8''), 7.55 (dd, $J_{8''-9''}$ = 5.0, $J_{8''-6''}$ = 1.3 Hz, 1H, H9''), 7.44 (s, 2H, NH_2), 6.06 (d, $J_{1'-2'}$ = 7.0 Hz, 1H, H1'), 5.86 (d, $J_{OH-3'}$ = 4.5 Hz, 1H, 3'OH), 5.67 (d, $J_{OH-2'}$ = 6.2 Hz, 1H, 2'OH), 4.69 (m, 1H, H2'), 4.55 (d, $J_{4'-3'}$ = 2.2 Hz, 1H, H4'), 4.35 (m, 1H, H3') ppm.

^{13}C NMR (101 MHz, DMSO-d_6) δ 168.27 (**CO**NH), 156.27 (C6), 152.63 (C2), 149.11 (C4), 140.99 (C7''), 140.34 (C8), 136.95 (C1''), 131.10 (C4''), 127.05 (C8''), 127.05 (C3''), 126.45 (C9''), 126.03 (C2''), 120.62 (C6''), 120.28 (C5), 87.90 (C1'), 84.50 (C4'), 73.21 (C3'), 72.53 (C2') ppm.

HRMS (ESI) Calculated for $C_{20}H_{19}O_4N_6S$, $[M + H]^+$ m/z: 439.11830, found 439.11814.

$[\alpha]_D^{20}$ -75.3 (*c* 0.299, DMSO).

Melting point: 266.1 °C, decomposition.

(2*S*,3*S*,4*R*,5*R*)-*N*-(4-(1*H*-Pyrazol-4-yl)phenyl)-5-(6-amino-9*H*-purin-9-yl)-3,4-dihydroxytetrahydrofuran-2-carboxamide (**12i**)

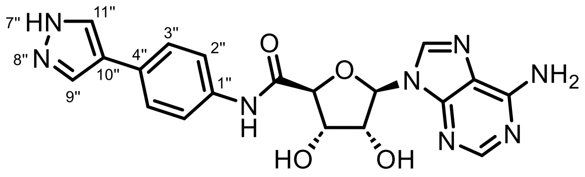

Compound **12i** (50 mg, yield 29 %) was prepared from **11i** (190 mg, 0.411 mmol) according to *General procedure F* and obtained as a white solid**.**

^{1}H NMR (400 MHz, DMSO-d_6) δ 12.90 (s, 1H, NH), 10.48 (s, 1H, CO**NH**), 8.47 (s, 1H, H8), 8.15 (s, 1H, H2), 8.22–7.77 (bs, 2H, H7′′, H9′′), 7.64–7.58 (s, 4H, H2′′, H3′′), 7.44 (s, 2H, NH_2), 6.05 (d, $J_{1'\text{-}2'}$ = 7.1 Hz, 1H, H1′), 5.84 (d, $J_{OH\text{-}3'}$ = 4.5 Hz, 1H, 3′OH), 5.66 (d, $J_{OH\text{-}2'}$ = 6.3 Hz, 1H, 2′OH), 4.68 (m, 1H, H2′), 4.53 (d, $J_{4'\text{-}3'}$ = 2.1 Hz, 1H, H4′), 4.33 (m, 1H, H3′) ppm.

^{13}C NMR (101 MHz, DMSO-d_6) δ 168.08 (**CO**NH), 156.24 (C6), 152.56 (C2), 149.07 (C4), 140.36 (C8), 135.79 (C1′′), 128.97 (C4′′ or C8′′), 125.40 (C3′′), 120.79 (C2′′), 120.70 (C4′′ or C8′′), 119.52 (C5), 87.88 (C1′), 84.50 (C4′), 73.19 (C3′), 72.49 (C2′) ppm. Signals of C7′′ and C9′′ were not detected.

HRMS (ESI) Calculated for $C_{19}H_{19}O_4N_8$, $[M + H]^+$ m/z: 423.15238, found 423.15221.

$[\alpha]_D^{20}$ -54.39 (*c* 0.285, DMSO).

Melting point: 287.3 °C, decomposition.

(2*S*,3*S*,4*R*,5*R*)-5-(6-Amino-9*H*-purin-9-yl)-*N*-(4-(3,5-dimethylisoxazol-4-yl)phenyl)-3,4-dihydroxytetrahydrofuran-2-carboxamide (**12j**)

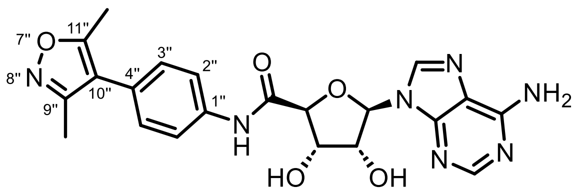

Compound **12j** (114 mg, yield 83 %) was prepared from **11j** (150 mg, 0.305 mmol) according to the *General procedure F* and obtained as a white solid.

^{1}H NMR (400 MHz, DMSO-d_6) δ 10.57 (s, 1H, CO**NH**), 8.47 (s, 1H, H8), 8.17 (s, 1H, H2), 7.78–7.70 (m, 2H, H2′′), 7.43 (s, 2H, NH_2), 7.42–7.36 (m, 2H, H3′′), 6.06 (d, $J_{1'-2'}$ = 7.1 Hz, 1H, H1′), 5.87 (bs, 1H, 3′OH), 5.67 (bs, 1H, 2′OH), 4.69 (m, 1H, H2′), 4.55 (d, $J_{4'-3'}$ = 2.1 Hz, 1H, H4′), 4.34 (m, 1H, H3′), 2.41 (s, 3H, C7′′/9′′-**Me**), 2.23 (s, 3H, C7′′/9′′-**Me**) ppm.

^{13}C NMR (101 MHz, DMSO-d_6) δ 168.43 and 164.90 (C7′′, C9′′), 158.14 (**CO**NH), 156.25 (C6), 152.65 (C2), 149.12 (C4), 140.30 (C8), 137.26 (C1′′), 129.33 (C3′′), 125.51 (C4′′), 120.56 (C2′′), 119.50 (C5), 115.54 (C8′′), 87.84 (C1′), 84.45 (C4′), 73.24 (C3′), 72.50 (C2′), 11.32 (C7′′/9′′-**Me**), 10.48 (C7′′/9′′-**Me**) ppm.

HRMS (ESI) Calculated for $C_{21}H_{22}O_5N_7$, $[M + H]^+$ m/z: 452.16769, found 452.16738.

$[\alpha]_D^{20}$ -62.7 (*c* 0.305, DMSO).

Melting point: 184.1 °C, decomposition.

(2*S*,3*S*,4*R*,5*R*)-5-(6-Amino-9*H*-purin-9-yl)-*N*-(2,3-dihydrobenzo[*b*][1,4]dioxin-6-yl)-3,4-dihydroxytetrahydrofuran-2-carboxamide (**12k**)

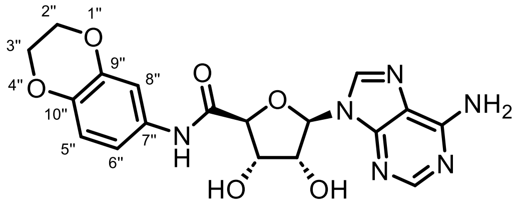

Compound **12k** (173 mg, yield 86 %) was prepared from **11k** (220 mg, 0.484 mmol) according to the *General procedure F* and obtained as a white solid.

^{1}H NMR (400 MHz, DMSO-d_6) δ 10.35 (s, 1H, CO**NH**), 8.44 (s, 1H, H8), 8.11 (s, 1H, H2), 7.40 (s, 2H, NH_2), 7.21 (d, $J_{8''\text{-}6''}$ = 2.5 Hz, 1H, H8''), 6.99 (dd, $J_{6''\text{-}5''}$ = 8.7, $J_{6''\text{-}8''}$ = 2.5 Hz, 1H, H6''), 6.84 (d, $J_{5''\text{-}6''}$ = 8.7 Hz, 1H, H5''), 6.02 (d, $J_{1'\text{-}2'}$ = 7.1 Hz, 1H, H1'), 5.83 (d, $J_{OH\text{-}3'}$ = 4.5 Hz, 1H, 3'OH), 5.67 (d, $J_{OH\text{-}2'}$ = 6.3 Hz, 1H, 2'OH), 4.64 (m, 1H, H2'), 4.48 (d, $J_{4'\text{-}3'}$ = 2.1 Hz, 1H, H4'), 4.29 (m, 1H, H3'), 4.26–4.16 (m, 4H, H2'', H3'') ppm.

^{13}C NMR (101 MHz, DMSO-d_6) δ 168.04 (**CO**NH), 156.33 (C6), 152.70 (C2), 149.18 (C4), 143.12 (C10''), 140.53 (C8), 140.21 (C9'), 131.56 (C7''), 119.58 (C5), 117.04 (C5''), 113.76 (C6''), 109.72 (C8''), 88.01 (C1'), 84.64 (C4'), 73.31 (C3'), 72.65 (C2'), 64.34 and 64.12 (C2'', C3'') ppm.

HRMS (ESI) Calculated for $C_{18}H_{19}O_6N_6$, $[M + H]^+$ m/z: 415.13606, found 415.13617.

$[\alpha]_D^{20}$ -52.4 (*c* 0.338, DMSO).

Melting point: 194.8-196.3 °C.

(2*S*,3*S*,4*R*,5*R*)-5-(6-Amino-9*H*-purin-9-yl)-3,4-dihydroxy-*N*-(4-methyl-2-oxo-2*H*-chromen-7-yl)tetrahydrofuran-2-carboxamide (**12l**)

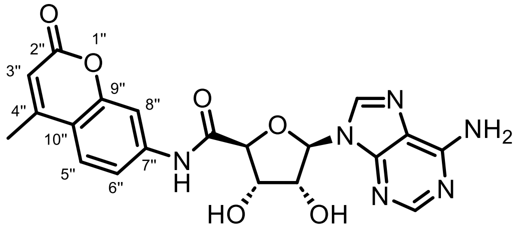

Compound **12l** (114 mg, yield 59 %) was prepared from **11l** (210 mg, 0.439 mmol) according to the *General procedure F* and obtained as a white solid**.**

^{1}H NMR (400 MHz, DMSO-d_6) δ 10.71 (s, 1H, CO**NH**), 8.47 (s, 1H, H8), 8.14 (s, 1H, H2), 7.81 (d, $J_{8''-6''}$ = 2.1 Hz, 1H, H8''), 7.78 (d, $J_{5''-6''}$ = 8.7 Hz, 1H, H5''), 7.54 (dd, $J_{6''-5''}$ = 8.7, $J_{6''-8''}$ = 2.1 Hz, 1H, H6''), 7.41 (s, 2H, NH_2), 6.30 (d, $J_{3''\text{-Me}}$ = 1.3 Hz, 1H, H3''), 6.08 (d, $J_{1'-2'}$ = 6.9 Hz, 1H, H1'), 5.86 (d, $J_{\text{OH-}3'}$ = 4.6 Hz, 1H, 3'OH), 5.67 (d, $J_{\text{OH-}2'}$ = 6.2 Hz, 1H, 2'OH), 4.71 (m, 1H, H2'), 4.58 (d, $J_{4'-3'}$ = 2.3 Hz, 1H, H4'), 4.40 (m, 1H, H3'), 2.42 (d, $J_{\text{Me-}3''}$ = 1.3 Hz, 3H, 4''-**Me**) ppm.

^{13}C NMR (101 MHz, DMSO-d_6) δ 168.98 (**CO**NH), 159.92 (C2''), 156.21 (C6), 153.49 (C9''), 153.02 (C4''), 152.60 (C2), 149.23 (C4), 141.23 (C7''), 140.16 (C8), 125.95 (C5''), 119.44 (C5), 116.12 (C6''), 115.71 (C10''), 112.70 (C3''), 106.84 (C8''), 87.82 (C1'), 84.23 (C4'), 73.15 (C3'), 72.54 (C2'), 17.97 (C4''-**Me**) ppm.

HRMS (ESI) Calculated for $C_{20}H_{19}O_6N_6$, $[M + H]^+$ m/z: 439.13606, found 439.13653.

$[\alpha]_D^{20}$ -83.5 (*c* 0.310, DMSO).

Melting point: 201.2 °C, decomposition.

(2*S*,3*S*,4*R*,5*R*)-5-(6-Amino-9*H*-purin-9-yl)-3,4-dihydroxy-*N*-(2-oxo-4-(trifluoromethyl)-2*H*-chromen-7-yl)tetrahydrofuran-2-carboxamide (**12m**)

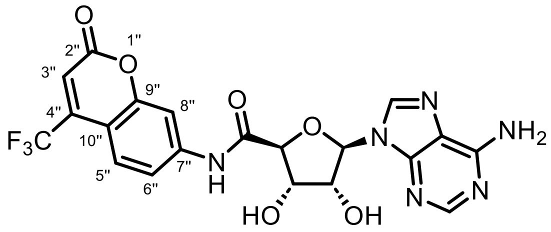

Compound **12m** (145 mg, yield 82 %) was prepared from **11m** (218 mg, 0.409 mmol) according to the *General procedure F* and obtained as a white solid.

^{1}H NMR (400 MHz, DMSO-d_6) δ 10.82 (s, 1H, CO**NH**), 8.45 (s, 1H, H8), 8.13 (s, 1H, H2), 7.87 (d, $J_{8''\text{-}6''}$ = 2.1 Hz, 1H, H8''), 7.71 (dq, $J_{5''\text{-}6''}$ = 8.9, $J_{5''\text{-CF3}}$ =1.8 Hz, 1H, H5''), 7.56 (dd, $J_{6''\text{-}5''}$ = 8.9, $J_{6''\text{-}8''}$ = 2.1 Hz, 1H, H6''), 7.34 (s, 2H, NH_2), 6.88 (s, 1H, H3''), 6.05 (d, $J_{1'\text{-}2'}$ = 6.9 Hz, 1H, H1'), 5.95 (d, $J_{\text{OH-}3'}$ = 4.7 Hz, 1H, 3'OH), 5.80 (d, $J_{\text{OH-}2'}$ = 6.2 Hz, 1H, 2'OH), 4.69 (m, 1H, H2'), 4.58 (d, $J_{4'\text{-}3'}$ = 2.3 Hz, 1H, H4'), 4.39 (m, 1H, H3') ppm.

^{13}C NMR (101 MHz, DMSO-d_6) δ 169.74 (**CO**NH), 159.15 (C2''), 156.44 (C6), 154.88 (C9''), 153.14 (C2), 149.57 (C4), 142.48 (C7''), 140.75 (C8), 139.70 (q, $J_{4''\text{-CF3}}$ = 32.3 Hz, C4''), 125.95 (C5''), 121.99 (q, $J_{\text{C-F3}}$ = 275.2 Hz, 4''-**CF_3**), 119.69 (C5), 117.42 (C6''), 115.34 (q, $J_{3''\text{-CF3}}$ = 6.5 Hz, C3''), 109.50 (C10''), 107.96 (C8''), 88.31 (C1'), 84.62 (C4'), 73.57 (C3'), 72.97 (C2') ppm.

^{19}F NMR (376 MHz, DMSO-d_6) δ -63.64.

HRMS (ESI) Calculated for $C_{20}H_{16}O_6N_6F_3$, $[M + H]^+$ m/z: 493.10779, found 493.10757.

$[\alpha]_D^{20}$ -89.6 (*c* 0.299, DMSO).

Melting point: 256.8 °C, decomposition.

(2*S*,3*S*,4*R*,5*R*)-5-(6-Amino-9*H*-purin-9-yl)-3,4-dihydroxy-*N*-(4-methyl-2-oxo-1,2-dihydroquinolin-7-yl)tetrahydrofuran-2-carboxamide (**12n**)

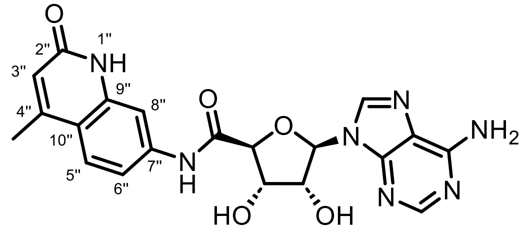

Compound **12n** (40 mg, yield 11 %) was prepared from **11n** (400 mg, 0.838 mmol) according to the *General procedure F* and obtained as an off-white solid.

^{1}H NMR (400 MHz, DMSO-d_6) δ 11.66 (s, 1H, H1′′), 10.70 (s, 1H, CO**NH**), 8.46 (s, 1H, H8), 8.17 (s, 1H, H2), 7.81 (d, $J_{8''-6''}$ = 2.1 Hz, 1H, H8′′), 7.71 (d, $J_{5''-6''}$ = 8.8 Hz, 1H, H5′′), 7.39 (s, 2H, NH_2), 7.34 (dd, $J_{6''-5''}$ = 8.8, $J_{6''-8''}$ = 2.1 Hz, 1H, H6′′), 6.31 (s, 1H, H3′′), 6.05 (d, $J_{1'-2'}$ = 6.9 Hz, 1H, H1′), 5.92 (bs, 1H, 2′OH or 3′OH), 5.76 (bs, 1H, 2′OH or 3′OH), 4.65 (dd, $J_{2'-1'}$ = 6.9, $J_{2'-3'}$ = 4.6 Hz, 1H, H2′), 4.57 (d, $J_{4'-3'}$ = 2.2 Hz, 1H, H4′), 4.35 (dd, $J_{3'-2'}$ = 4.6, $J_{3'-4'}$ = 2.2 Hz, 1H, H3′), 2.39 (d, $J_{Me-3''}$ = 1.2 Hz, 3H, 4′′-**Me**) ppm.

^{13}C NMR (101 MHz, DMSO-d_6) δ 169.00 (**CO**NH), 162.48 (C2′′), 156.39 (C6), 153.09 (C2), 149.28 (C4), 148.29 (C4′′), 140.57 (C8), 139.82 and 139.49 (C7”, C9′′), 125.76 (C5′′), 119.63 (C5′), 119.55 (C3”), 116.61 (C10′′), 114.99 (C6′′), 106.14 (C8′′), 88.23 (C1′), 84.63 (C4′), 73.46 (C3′), 72.89 (C2′), 18.65 (4′′**Me**) ppm.

HRMS (ESI) Calculated for $C_{20}H_{20}O_5N_7$, [*M* + H]$^+$ m/z: 438.15204, found 438.15239.

[α]$_D^{20}$ -68.4 (*c* 0.191, DMSO).

Melting point: 250.1 °C, decomposition.

(2*S*,3*S*,4*R*,5*R*)-5-(6-Amino-9*H*-purin-9-yl)-3,4-dihydroxy-*N*-(2-oxo-1,2,3,4-tetrahydroquinolin-7-yl)tetrahydrofuran-2-carboxamide (**12o**)

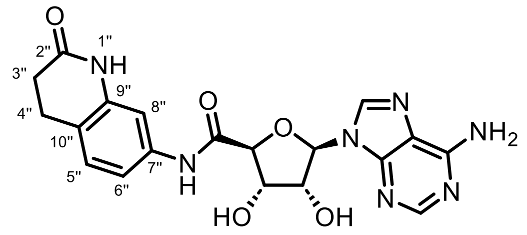

Compound **12o** (135 mg, yield 56 %) was prepared from **11o** (265 mg, 0.569 mmol) according to the *General procedure F* and obtained as an off-white solid.

^{1}H NMR (400 MHz, DMSO-d_6) δ 10.52 (s, 1H, CO**NH**), 10.15 (s, 1H, H1''), 8.41 (s, 1H, H8), 8.12 (s, 1H, H2), 7.30 (s, 2H, NH_2), 7.19 (d, $J_{8''-6''}$ = 2.0 Hz, 1H, H8''), 7.14 (d, $J_{5''-6''}$ = 8.3 Hz, 1H, H5''), 7.03 (dd, $J_{6''-5''}$ = 8.3, $J_{6''-8''}$ = 2.0 Hz, 1H, H6''), 6.02–5.98 (m, 2H, H1', 3'OH), 5.87 (d, $J_{OH-2'}$ = 6.2 Hz, 1H, 2'OH), 4.62 (m, 1H, H2'), 4.50 (d, $J_{4'-3'}$ = 2.0 Hz, 1H, H4'), 4.29 (m, 1H, H3'), 2.84–2.78 (m, 2H, 4''), 2.46–2.36 (m, 2H, 3'') ppm.

^{13}C NMR (101 MHz, DMSO-d_6) δ 172.26 (C2''), 169.12 (**CO**NH), 156.64 (C6), 153.52 (C2), 149.53 (C4), 141.39 (C8), 138.85 (C9''), 137.14 (C7''), 128.91 (C5''), 120.88 (C10''), 119.96 (C5), 115.55 (C6''), 108.52 (C8''), 88.78 (C1'), 85.23 (C4'), 73.88 (C3'), 73.21 (C2'), 30.98 (C3''), 24.82 (C4'') ppm.

HRMS (ESI) Calculated for $C_{19}H_{20}O_5N_7$, $[M + H]^+$ m/z: 426.15204, found 426.15176.

$[\alpha]_D^{20}$ -62.8 (*c* 0.300, DMSO).

Melting point: 231.0-232.2°C.

(2*S*,3*S*,4*R*,5*R*)-5-(6-Amino-9*H*-purin-9-yl)-3,4-dihydroxy-*N*-(quinolin-7-yl)tetrahydrofuran-2-carboxamide (**12p**)

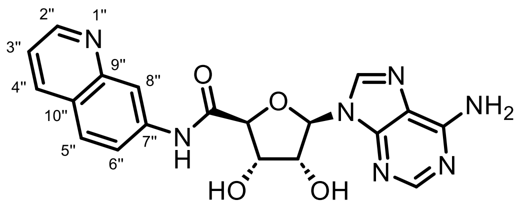

Compound **12p** (199 mg, yield 87 %) was prepared from **11p** (250 mg, 0.559 mmol) according to the *General procedure F* and obtained as an off-white solid.

^{1}H NMR (400 MHz, DMSO-d_6) δ 10.75 (s, 1H, CO**NH**), 8.87 (dd, $J_{2''-3''}$ = 4.2, $J_{2''-4''}$ = 1.7 Hz, 1H, H2''), 8.51 (s, 1H, H8), 8.44 (d, $J_{8''-6''}$ = 2.0 Hz, 1H, H8''), 8.31 (ddd, $J_{4''-3''}$ = 8.4, $J_{4''-2''}$ = 1.7, $J_{4''-5''}$ = 0.6 Hz, 1H, H4''), 8.16 (s, 1H, H2), 7.98 (d, $J_{5''-6''}$ = 8.8 Hz, 1H, H5''), 7.78 (dd, $J_{6''-5''}$ = 8.8, $J_{6''-8''}$ = 2.0 Hz, 1H, H6''), 7.46 (dd, $J_{3''-4''}$ = 8.4, $J_{3''-2''}$ = 4.2 Hz, 1H, H3''), 7.44 (s, 2H, NH_2), 6.09 (d, $J_{1'-2'}$ = 7.0 Hz, 1H, H1'), 5.88 (d, $J_{OH-3'}$ = 4.6 Hz, 1H, 3'OH), 5.69 (d, $J_{OH-2'}$ = 6.2 Hz, 1H, 2'OH), 4.73 (m, 1H, H2'), 4.62 (d, $J_{4'-3'}$ = 2.2 Hz, 1H, H4'), 4.41 (m, 1H, H3') ppm.

^{13}C NMR (101 MHz, DMSO-d_6) δ 168.83 (**CO**NH), 156.25 (C6), 152.61 (C2), 151.06 (C2''), 149.20 (C4), 148.22 (C9''), 140.25 (C8), 138.78 (C7''), 135.61 (C4''), 128.60 (C5''), 124.87 (C10''), 120.91 (C6''), 120.45 (C3''), 119.45 (C5), 117.48 (C8''), 87.85 (C1'), 84.39 (C4'), 73.21 (C3'), 72.59 (C2') ppm.

HRMS (ESI) Calculated for $C_{19}H_{18}O_4N_7$, $[M + H]^+$ m/z: 408.14148, found 408.14172.

$[\alpha]_D^{20}$ -88.2 (*c* 0.306, DMSO).

Melting point: 192.9-193.6 °C.

(2*S*,3*S*,4*R*,5*R*)-5-(4-Amino-5-(pyrimidin-5-ylethynyl)-7*H*-pyrrolo[2,3-*d*]pyrimidin-7-yl)-3,4-dihydroxy-*N*-(4-methyl-2-oxo-2*H*-chromen-7-yl)tetrahydrofuran-2-carboxamide (**18l**)

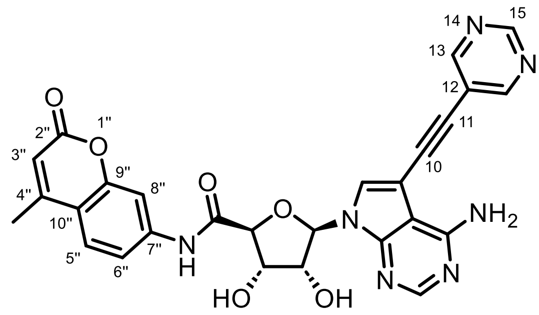

Compound **18l** (20 mg, yield 16 %) was prepared from **17l** (180 mg, 0.234 mmol) according to the *General procedure F* and obtained as a pale-yellow solid.

^{1}H NMR (400 MHz, DMSO-d_6) δ 10.79 (s, 1H, CO**NH**), 9.16 (s, 1H, H15), 9.07 (s, 2H, H13), 8.18 (s, 1H, H2), 8.14 (s, 1H, H8), 7.81 (d, $J_{8''\text{-}6''}$ = 2.1 Hz, 1H, H8′′), 7.76 (d, $J_{5''\text{-}6''}$ = 8.7 Hz, 1H, H5′′), 7.58 (dd, $J_{6''\text{-}5''}$ = 8.7, $J_{6''\text{-}8''}$ = 2.1 Hz, 1H, H6′′), 6.91 (s, 2H, NH_2), 6.30 (bs, 1H, H3′′), 6.26 (d, $J_{1'\text{-}2'}$ = 6.5 Hz, 1H, H1′), 5.89 (bs, 1H, 3′OH), 5.69 (d, $J_{OH\text{-}2'}$ = 5.3 Hz, 1H, 2′OH), 4.60 (d, $J_{4'\text{-}3'}$ = 2.5 Hz, 1H, H4′), 4.57 (m, 1H, H2′), 4.36 (m, 1H, H3′), 2.41 (bs, 3H, 4′′-**Me**) ppm.

^{13}C NMR (101 MHz, DMSO-d_6) δ 169.36 (**CO**NH), 159.91 (C2′′), 158.60 (C13), 157.54 (C6), 156.39 (C15), 153.52 (C9′′), 153.09 (C2), 153.03 (C10′′), 150.29 (C4), 141.43 (C7′′), 128.44 (C8), 125.97 (C5′′), 119.32 (C12), 115.92 (C6′′), 115.65 (C4′′), 112.67 (C3′′), 106.58 (C8′′), 101.97 (C5), 94.23 and 89.32 (C7, C10), 87.59 (C1′), 84.95 (C11), 83.76 (C4′), 73.69 (C2′), 73.20 (C3′), 17.99 (4′′-Me) ppm.

HRMS (ESI) Calculated for $C_{27}H_{22}O_6N_7$, $[M + H]^+$ m/z: 540.16261, found 540.16238.

$[\alpha]_D^{20}$ +74.1 (*c* 0.239, DMSO).

Melting point: 202.9 °C, decomposition.

(2*S*,3*S*,4*R*,5*R*)-5-(4-Amino-5-(pyrimidin-5-ylethynyl)-7*H*-pyrrolo[2,3-*d*]pyrimidin-7-yl)-3,4-dihydroxy-*N*-(4-methyl-2-oxo-1,2-dihydroquinolin-7-yl)tetrahydrofuran-2-carboxamide (**18n**)

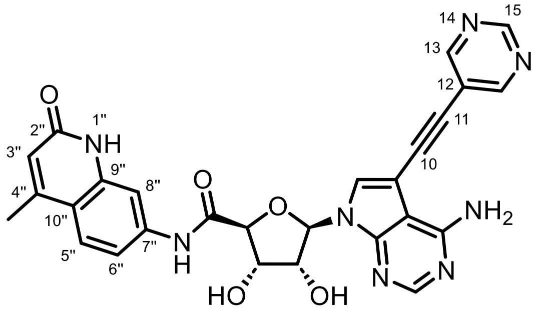

Compound **18n** (45 mg, yield 33 %) was prepared from **17n** (197 mg, 0.257 mmol) according to the *General procedure F* and obtained as a pale-brown solid.

^{1}H NMR (400 MHz, DMSO-d_6) δ 11.60 (s, 1H, H1′), 10.69 (s, 1H, **CO**NH), 9.17 (s, 1H, H15), 9.07 (s, 2H, H13), 8.19 (s, 1H, H2), 8.18 (s, 1H, H8), 7.85 (d, $J_{8''-6''}$ = 2.1 Hz, 1H, H8′′), 7.68 (d, $J_{5''-6''}$ = 8.7 Hz, 1H, H5′′), 7.37 (dd, $J_{6''-5''}$ = 8.7, $J_{6''-8''}$ = 2.1 Hz, 1H, H6′′), 6.92 (bs, 2H, NH_2), 6.29 (d, $J_{3''-Me}$ = 1.2 Hz, 1H, H3′′), 6.23 (d, $J_{1'-2'}$ = 6.4 Hz, 1H, H1′), 5.97–5.65 (m, 2H, 2′OH, 3′OH), 4.60 (d, $J_{4'-3'}$ = 2.7 Hz, 1H, H4′), 4.55 (dd, $J_{2'-1'}$ = 6.4, $J_{2'-3'}$ = 4.6 Hz, 1H, H2′), 4.32 (dd, $J_{3'-2'}$ = 4.6, $J_{3'-4'}$ = 2.7 Hz, 1H, H3′), 2.39 (d, $J_{Me-3''}$ = 1.3 Hz, 3H, 4′′-**Me**) ppm.

^{13}C NMR (101 MHz, DMSO-d_6) δ 169.07 (**CO**NH), 161.95 (C2′′), 158.62 (C13), 157.56 (C6), 156.43 (C15), 153.16 (C2), 150.13 (C4), 147.58 (C4′′), 139.80 and 139.42 (C7′′, C9′′),128.54 (C8), 125.40 (C5′′), 119.43 (C3′′), 119.33 (C12), 116.13 (C10′′), 114.19 (C6′′), 105.43 (C8′′), 101.99 (C5), 94.09 and 89.34 (C7, C10), 87.81 (C1′), 84.91 (C11), 83.79 (C4′), 73.86 (C2′), 73.25 (C3′), 18.38 (4′′Me) ppm.

HRMS (ESI) Calculated for $C_{27}H_{23}O_5N_8$, [*M* + H]$^+$ m/z: 539.17859, found 539.17883.

$[\alpha]_D^{20}$ -85.8 (*c* 0.152, DMSO).

Melting point: 255.8 °C, decomposition.

(2*S*,3*S*,4*R*,5*R*)-5-(4-Amino-5-(pyrimidin-5-ylethynyl)-7*H*-pyrrolo[2,3-*d*]pyrimidin-7-yl)-3,4-dihydroxy-*N*-methyl-*N*-(4-methyl-2-oxo-1,2-dihydroquinolin-7-yl)tetrahydrofuran-2-carboxamide (**18q**)

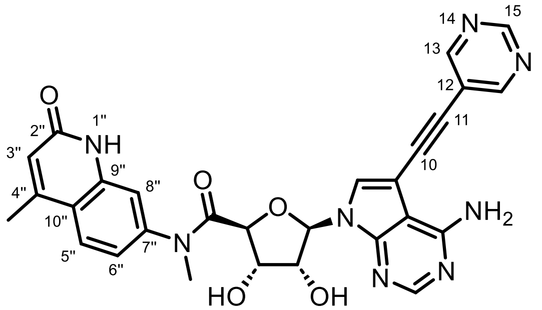

Compound **18q** (15 mg, yield 14 %) was prepared from **17q** (150 mg, 0.192 mmol) according to the *General procedure F* and obtained as an off-white solid.

^{1}H NMR (500 MHz, DMSO) δ 11.69 (s, 1H, H1′), 9.17 (s, 1H, H15), 9.10 (s, 2H, H13), 8.19 (s, 1H, H8), 8.16 (s, 1H, H2), 7.80 (d, $J_{5''-6''}$ = 8.5 Hz, 1H, H5″), 7.21 (d, $J_{8''-6''}$ = 1.4 Hz, 1H, H8″), 7.18 (dd, $J_{6''-5''}$ = 8.4, $J_{6''-8''}$ = 2.2 Hz, 1H, H6″), 6.87 (s, 2H, NH_2), 6.44 (s, 1H, H3″), 6.21 (s, 1H, H1′), 5.60 (s, 1H, 2′OH), 5.50 (s, 1H, 3′OH), 4.32–4.28 (m, 1H, H2′), 4.35 (s, 1H, H4′), 4.32–4.28 (m, 1H, H3′), 3.29 (s, 3H, CO**NMe**), 2.44 (s, 3H, 4″-**Me**) ppm.

^{13}C NMR (126 MHz, DMSO) δ 169.33 (**CO**NMe), 161.65 (C2″), 158.62 (C13), 157.45 (C6), 156.38 (C15), 153.12 (C2), 150.47 (C4), 147.58 (C4″), 143.82 (C7″), 139.44 (C9″), 127.56 (C8), 126.52 (C5″), 121.31 (C3″), 120.44 (C6″), 119.34 (C12), 119.01 (C10″), 113.40 (C8″), 101.49 (C5), 94.39 and 89.41 (C7, C10), 86.85 (C1′), 84.90 (C11), 80.16 (C4′), 75.18 (C2′), 73.10 (C3′), 37.40 (CO**NMe**), 18.48 (4″-**Me**) ppm.

HRMS (ESI) Calculated for $C_{28}H_{25}O_5N_8$, $[M + H]^+$ m/z: 553.19424, found 553.19457.

$[\alpha]_D^{20}$ +152.3 (*c* 0.168, DMSO).

Melting point: 275.2 °C, decomposition.

Methyl 4-((((2*R*,3*S*,4*R*,5*R*)-5-(6-amino-9*H*-purin-9-yl)-3,4-dihydroxytetrahydrofuran-2-yl)methyl)carbamoyl)benzoate (**21a**)

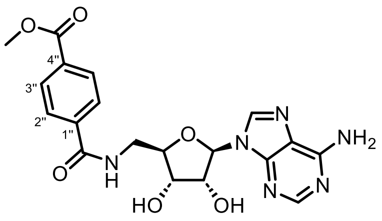

Compound **21a** (109 mg, yield 26 % over 2 steps) was prepared according to the *General procedures E* and *F* and obtained as a white solid.

^{1}H NMR (400 MHz, DMSO-d_6) δ 8.91 (t, $J_{NH\text{-}5'}$ = 5.8 Hz, 1H, CO**NH**), 8.36 (s, 1H, H8), 8.06–8.00 (m, 3H, H3′′, H2), 7.97 (m, 2H, H2′′), 7.31 (s, 2H, NH_2), 5.87 (d, $J_{1'\text{-}2'}$ = 6.1 Hz, 1H, H1′), 5.47 (d, $J_{OH\text{-}2'}$= 6.2 Hz, 1H, 2′OH), 5.30 (d, $J_{OH\text{-}3'}$ = 4.8 Hz, 1H, 3′OH), 4.76 (m, 1H, H2′), 4.20 (m, 1H, H3′), 4.09 (m, 1H, H4′), 3.88 (s, 3H, 4′′-**OMe**), 3.64 (m, 2H, H5′) ppm.

^{13}C NMR (101 MHz, DMSO-d_6) δ 165.89 (1′′-**CO**NH), 165.69 (4′′-**CO**OMe), 156.10 (C6), 152.51 (C2), 149.27 (C4), 140.29 (C8), 138.55 (C1′′), 131.76 (C4′′), 129.12 (C3′′), 127.66 (2′′), 119.41 (C5), 87.71 (C1′), 83.04 (C4′), 72.51 (C2′), 71.30 (C3′), 52.37 (4′′-CO**OMe**), 41.80 (C5′) ppm.

HRMS (ESI) Calculated for $C_{19}H_{21}O_6N_6$, $[M + H]^+$ m/z: 429.15171, found 429.15140.

$[\alpha]_D^{20}$ -53.4 (*c* 0.290, DMSO).

Melting point: 235.0 °C, decomposition.

***N*-(((2*R*,3*S*,4*R*,5*R*)-5-(6-Amino-9*H*-purin-9-yl)-3,4-dihydroxytetrahydrofuran-2-yl)methyl)-2,3-dihydrobenzo[*b*][1,4]dioxine-6-carboxamide** (**21k**)

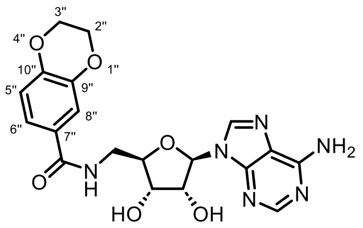

Compound **21k** (149 mg, yield 36 % over 2 steps) was prepared according to the *General procedures E* and *F* and obtained as a white solid.

^{1}H NMR (400 MHz, DMSO-d_6) δ 8.54 (t, $J_{NH\text{-}5'}$ = 5.8 Hz, 1H, CO**NH**), 8.33 (s, 1H, H8), 8.09 (s, 1H, H2), 7.39 (d, $J_{8''\text{-}6''}$ = 2.1 Hz, 1H, H8''), 7.36 (dd, $J_{6''\text{-}5''}$ = 8.3, $J_{6''\text{-}8''}$ = 2.1 Hz, 1H, H6''), 7.29 (s, 2H, NH_2), 6.90 (d, $J_{5''\text{-}6''}$ = 8.3 Hz, 1H, H5''), 5.85 (d, $J_{1'\text{-}2'}$ = 6.4 Hz, 1H, H1'), 5.49 (d, $J_{OH\text{-}2'}$ = 6.1 Hz, 1H, 2'OH), 5.31 (dd, $J_{OH\text{-}3'}$ = 4.6, 1.4 Hz, 1H, 3'OH), 4.72 (m, 1H, H2'), 4.30–4.21 (m, 4H, H2'', H3''), 4.16–4.11 (m, 1H, H3'), 4.09–4.02 (m, 1H, H4') ppm. Signal of H5' is overlapped with water signal but is visible in HSQC.

^{13}C NMR (101 MHz, DMSO-d_6) δ 166.22 (**CO**NH), 156.23 (C6), 152.80 (C2), 149.45 (C4), 146.24 (C10''), 143.08 (C9''), 140.54 (C8), 127.51 (C7''), 120.95 (C6''), 119.56 (C5), 116.99 (C5''), 116.46 (C8''), 87.81 (C1'), 83.54 (C4''), 72.74 (C2'), 71.44 (C3'), 64.53 and 64.19 (C2'', C3''), 41.80 (C5') ppm.

HRMS (ESI) Calculated for $C_{19}H_{21}O_6N_6$, $[M + H]^+$ m/z: 429.15171, found 429.15157.

$[\alpha]_D^{20}$ -71.8 (*c* 0.308, DMSO).

Melting point: 155.9-156.7 °C.

***N*-(((2*R*,3*S*,4*R*,5*R*)-5-(6-Amino-9*H*-purin-9-yl)-3,4-dihydroxytetrahydrofuran-2-yl)methyl)-2,3-dihydrobenzo[*b*][1,4]dioxine-6-sulfonamide** (**22k**)

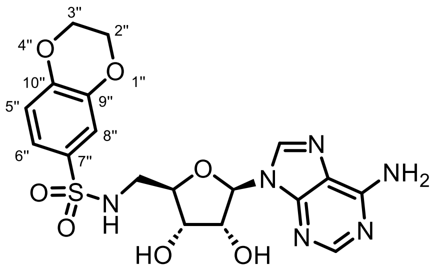

Compound **22k** (75 mg, yield 14 % over 2 steps) was prepared according to the *General procedures E* and *F* and obtained as a white solid.

^{1}H NMR (400 MHz, DMSO-d_6) δ 8.31 (s, 1H, H8), 8.14 (s, 1H, H2), 7.40 (s, 2H, NH_2), 7.25 (dd, $J_{6''\text{-}5''}$ = 8.4, $J_{6''\text{-}8''}$ = 2.2 Hz, 1H, H6''), 7.22 (d, $J_{8''\text{-}6''}$ = 2.2 Hz, 1H, H8''), 7.02 (d, $J_{5''\text{-}6''}$ = 8.4 Hz, 1H, H5''), 5.82 (d, $J_{1'\text{-}2'}$ = 6.6 Hz, 1H, H1'), 5.58–5.28 (bs, 2H, 2'OH, 3'OH), 4.69 (dd, $J_{2'\text{-}2'OH}$ = 6.7, $J_{2'\text{-}3'}$ = 5.1 Hz, 1H, H2'), 4.35–4.25 (m, 4H, H2'', H3''), 4.07 (dd, $J_{3'\text{-}2'}$ = 5.1, $J_{3'\text{-}4'}$ = 2.7 Hz, 1H, H3'), 4.00 (m, 1H, H4'), 3.03 (m, 2H, H5') ppm.

^{13}C NMR (101 MHz, DMSO-d_6) δ 156.26 (C6), 152.21 (C2), 148.79 (C4), 146.82 (C10''), 143.28 (C9''), 140.48 (C8), 132.50 (C7''), 120.00 (C6''), 119.54 (C5), 117.60 (C5''), 115.47 (C8''), 87.84 (C1'), 84.33 (C4'), 73.16 (C2'), 72.52 (C3'), 64.33 (C2''/C3''), 64.05(C2''/C3''), 44.88 (C5') ppm.

HRMS (ESI) Calculated for $C_{18}H_{21}O_7N_6S$, $[M + H]^+$ m/z: 465.11869, found 465.11859.

$[\alpha]_D^{20}$ -1.8 (*c* 0.207, DMSO).

Melting point: 201.1 °C, decomposition.

7-((((2*S*,3*S*,4*R*,5*R*)-5-(6-Amino-9*H*-purin-9-yl)-3,4-dihydroxytetrahydrofuran-2-yl)methyl)thio)-4-methyl-2*H*-chromen-2-one (**24**)

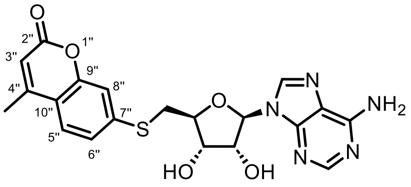

5'-deoxy-5'-chloroadenosine **23** (400 mg, 1.4 mmol, 1 eq.), 7-mercapto-4-methylcoumarin (323 mg, 1.68 mmol, 1.2 eq.), and K_2CO_3 (387 mg, 2.8 mmol, 2 eq.) were mixed in ethanol (10 mL) overnight at room temperature. The reaction mixture was then evaporated, dissolved in DMSO and purified using reverse phase chromatography (H_2O/MeCN, 5 to 40 %) to afford **24** (252 mg, yield 41%) as an off-white solid.

^{1}H NMR (400 MHz, DMSO-d_6) δ 8.34 (s, 1H, H8), 8.14 (s, 1H, H2), 7.60 (d, $J_{5''-6''}$ = 8.4 Hz, 1H, H5''), 7.34 (d, $J_{8''-6''}$ = 1.9 Hz, 1H, H8''), 7.29 (s, 2H, NH_2), 7.26 (dd, $J_{6''-5''}$ = 8.4, $J_{6''-8''}$ = 1.9 Hz, 1H, H6''), 6.31 (d, $J_{3''-CH3}$ = 1.3 Hz, 1H, H3''), 5.90 (d, $J_{1'-2'}$ = 5.6 Hz, 1H, H1'), 5.70–5.34 (bs, 2H, 2'OH, 3'OH), 4.82 (m, 1H, H2'), 4.25 (m, 1H, H3'), 4.07 (m, 1H, H4'), 3.59 (dd, J_{GEM} = 14.0, $J_{5'a-4'}$ = 5.4 Hz, 1H, H5'a), 3.45 (dd, J_{GEM} = 14.0, $J_{5'b-4'}$ = 7.3 Hz, 1H, H5'b), 2.39 (d, $J_{CH3-3''}$ = 1.3 Hz, 3H, 4''-**Me**) ppm.

^{13}C NMR (101 MHz, DMSO-d_6) δ 159.95 (C2''), 156.05 (C6), 155.99 and 153.28 (C9'', C10''), , 153.03 (C2), 152.60 (C4), 149.38 (C7''), 142.05 (C8), 125.46 (C5''), 122.77 (C6''), 119.15 (C5), 116.86 (C4''), 113.52 (C8''), 113.18 (C3''), 87.52 (C1'), 82.47 (C4'), 72.66 (C2'), 72.57 (C3'), 33.95 (C5'), 17.99 (C4''-**Me**) ppm.

HRMS (ESI) Calculated for $C_{20}H_{20}O_5N_5S$, $[M + H]^+$ m/z: 442.11797, found 442.11791.

$[\alpha]_D^{20}$ +4.9 (*c* 0.258, DMSO).

Melting point: 240.6 °C, decomposition.

7-((((2*S*,3*S*,4*R*,5*R*)-5-(6-Amino-9H-purin-9-yl)-3,4-dihydroxytetrahydrofuran-2-yl)methyl)sulfonyl)-4-methyl-2*H*-chromen-2-one (25)

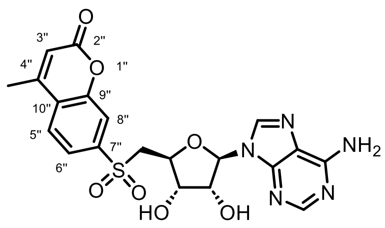

Synthesis of **25** was based on a published procedure.[9] Compound **24** (160 mg, 0.362 mmol, 1 eq.) and Oxone® (223 mg, 0.362 mmol, 1 eq.) were dissolved in water (25 mL) and stirred at room temperature for 4 hours. Then, the reaction mixture was evaporated, dissolved in DMSO and the desired product was purified using reverse phase flash chromatography (H_2O/MeCN, 5 to 35 %) to afford **25** (83 mg, yield 48 %) as a light-yellow solid.

^{1}H NMR (400 MHz, DMSO-*d*$_6$) δ 8.07 (s, 1H, H8), 7.98 (s, 1H, H2), 7.55 (d, $J_{8''\text{-}6''}$ = 1.8 Hz, 1H, H8''), 7.51 (dd, $J_{6''\text{-}5''}$ = 8.3, $J_{6''\text{-}8''}$ = 1.8 Hz, 1H, H6''), 7.45 (d, $J_{5''\text{-}6''}$ = 8.3 Hz, 1H, H5''), 7.17 (s, 2H, NH_2), 6.45 (d, $J_{3''\text{-}CH3}$ = 1.4 Hz, 1H, H3''), 5.72 (d, $J_{1'\text{-}2'}$ = 7.1 Hz, 1H, H1'), 5.52 (d, $J_{OH\text{-}3'}$ = 4.0 Hz, 1H, 3'OH), 5.46 (d, $J_{OH\text{-}2'}$ = 6.5 Hz, 1H, 2'OH), 5.02 (m, 1H, H2'), 4.37 (dm, $J_{4'\text{-}5'a}$ = 10.6 Hz, 1H, H4'), 4.27 (dd, J_{GEM} = 14.7, $J_{5'a\text{-}4'}$ = 10.6 Hz, 1H, H5'a), 4.10 (m, 1H, H3'), 3.83 (dd, J_{GEM} = 14.7, $J_{5'b\text{-}4'}$ = 2.1 Hz, 1H, H5'b), 2.34 (d, *J* = 1.4 Hz, 3H, 4''-**Me**) ppm.

^{13}C NMR (101 MHz, DMSO-*d*$_6$) δ 158.82 (C2''), 155.76 (C6), 152.30 (C2), 151.61 and 151.57 (C9'', C10''), 148.61 (C4), 142.00 (C7''), 140.49 (C8), 125.24 (C5''), 123.03 (C4''), 123.01 (C6''), 119.21 (C5), 116.93 (C3''), 116.27 (C8''), 87.75 (C1'), 79.93 (C4'), 73.30 (C3'), 70.89 (C2'), 58.14 (C5'), 18.09 (C4''-**Me**) ppm.

HRMS (ESI) Calculated for $C_{20}H_{20}O_7N_5S$, $[M + H]^+$ m/z: 474.10780, found 474.10772.

$[\alpha]_D^{20}$ +78.9 (*c* 0.254, DMSO).

Melting point: 205.6 °C, decomposition.

3. Biological data

3.1. Nsp14 protein expression and purification

E. coli BL21(DE3)RIL were transformed with plasmid DNA encoding SARS-CoV-2 nsp14, and the protein was expressed as a fusion protein with N-terminal His 8x and SUMO tag in LB media supplemented with 20µM $ZnSO_4$ at 18 °C for 16 hours. Cells were harvested by centrifugation, lysed by sonication in lysis buffer (50 mM Tris, pH 8, 300 mM NaCl, 5 mM MgSO4, 10% glycerol, 3 mM β-mercaptoethanol, 20 mM imidazole) and centrifuged again. The supernatant was mixed with Ni-NTA resin to bind the protein of interest (nsp14 with His 8x and SUMO tag). The resin was washed with lysis buffer, and the protein was eluted with lysis buffer supplemented with 300 mM imidazole. The eluted protein was then treated with Ulp1 protease and dialyzed to lysis buffer. The solution was passed through a Ni-NTA column to collect nsp14. Protein was concentrated and further purified using size exclusion chromatography on HiLoad 16/600 Superdex 200 gel filtration column (GE Healthcare) in 10 mM HEPES, pH 7.5, 150 mM NaCl. Fractions containing the pure protein were identified by SDS-PAGE, pooled, and concentrated to 5.5 mg/ml. The protein solution was then aliquoted, frozen in liquid nitrogen, and stored at -80 °C.

3.2. Methyltransferase activity assay

For measuring the inhibition of SARS-CoV-2 N7-methyltransferase nsp14, we followed the method detailed in our previous publication.[10] Briefly, the reactions were conducted in 1536-well plates with a total volume of 4 µl per well. The reaction mixture included 4 µM S-Adenosyl-L-methionine and 4 µM GpppA in a reaction buffer (5 mM Tris pH 8.0, 1 mM TCEP, 0.1 mg/ml BSA, 0.005% Triton X-100, 1 mM $MgCl_2$). The reaction was initiated with 20 nM SARS-CoV-2 nsp14 and incubated at 24°C for 30 minutes. Reactions were stopped with 5% formic acid and analyzed using an Echo mass spectrometry system coupled with a Sciex 6500 triple-quadrupole mass spectrometer. The rate of MTase activity was quantified by measuring S-adenosylhomocysteine (SAH) production in multiple-reaction-monitoring (MRM) mode with the interface heated to 350°C. The decluttering potential was 20 V, the entrance potential 10 V, and the collision energy 28 eV. Ten nanoliters were injected in the mobile phase (flow rate of 0.40 ml/min; 100% methanol). The characteristic product ion of SAH, m/z385.1 > 134.1, was used for quantification. For IC_{50} determination, compounds were tested in concentrations ranging from 12.5 pM to 12.5 µM. Dose-response curves were generated, and the IC_{50} values were calculated using a variable slope model in GraphPad software.

	IC_{50}	SD	Hill slope	SD	95 % CI (profile likelihood)	
					IC_{50}	Hill slope
**12a**	> 25	N. D.	N. D.	N. D.	> 25	N. D.
**12d**	> 25	N. D.	N. D.	N. D.	> 25	N. D.
**12k**	1.813	0.246	-1.01	0.132	1.56 to 2.11	-1.17 to -0.87
**12l**	0.364	0.039	-1.39	0.203	0.323 to 0.410	-1.64 to -1.18
**12m**	1.471	0.141	-1.01	0.085	1.32 to 1.64	-1.11 to -0.92
**12n**	0.350	0.038	-1.19	0.148	0.310 to 0.395	-1.38 to -1.04
**12o**	6.548	1.438	-0.91	0.175	5.12 to 8.38	-1.13 to -0.73
**12p**	4.813	2.540	-1.45	1.235	3.07 to 8.81	-3.80 to -1.02
**18l**	0.031	0.005	-1.09	0.138	0.025 to 0.037	-1.25 to -0.94
**18n**	0.043	0.005	-1.29	0.162	0.038 to 0.049	-1.50 to -1.13
**18q**	0.891	0.116	-1.02	0.107	0.769 to 1.032	-1.16 to -0.91
**22k**	15.350	2.554	-1.55	0.378	12.76 to 18.54	-2.02 to -1.17
**24**	3.665	0.396	-1.04	0.098	3.24 to 4.14	-1.17 to -0.93
**25**	> 25	N. D.	N. D.	N. D.	> 25	N. D.
**SIN**	0.455	0.051	-1.37	0.169	0.400 to 0.516	-1.58 to -1.19

Table S1. Inhibitory activity [μM] of synthesized compounds against SARS-CoV-2 nsp14 MTase and corresponding standard deviation (SD), Hill slope, and 95 % confidentiality interval (CI). Sinefungin (SIN) was used as a reference inhibitor. N. D., not determined.

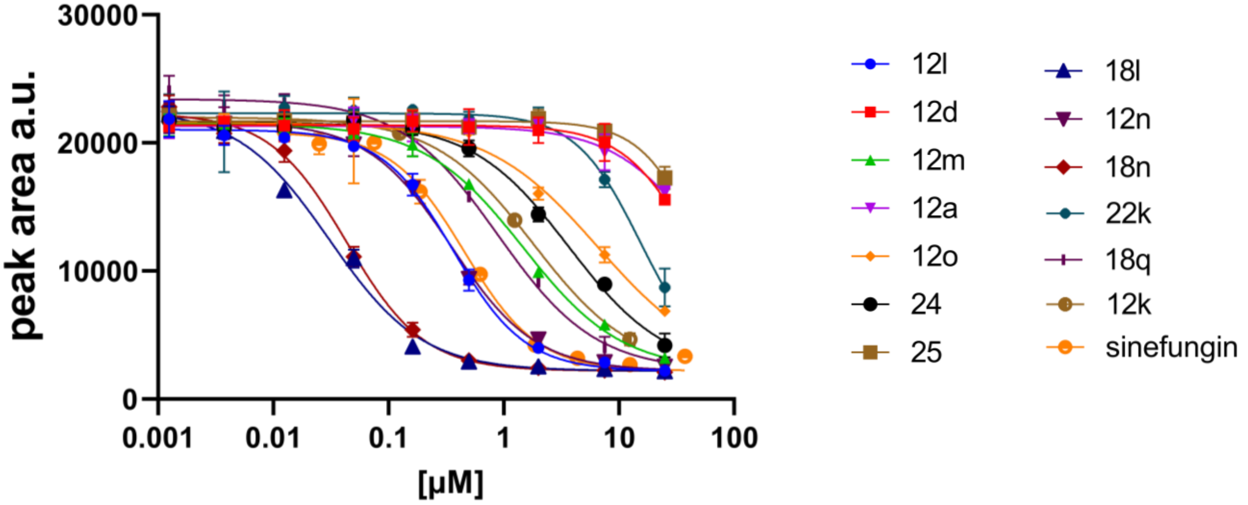

Figure S1. Concentration-dependent inhibition of SARS-CoV-2 nsp14 MTase by **12a**, **12d**, **12k**, **12l**, **12m**, **12n**, **12o**, **18l**, **18n**, **18q**, **22k**, **24**, **25**, and sinefungin.

3.3. Plasma stability assay

To determine plasma stability of the compounds, 10 µM of these were incubated with human pooled plasma from 50 donors (Biowest) for 10, 30, 60 and 120 min at 37 °C. The reactions were terminated by adding four volumes of ice-cold methanol, the samples were then mixed vigorously frozen at -20°C for 1h and left overnight at 8°C. After that, the samples were centrifuged, and the supernatant was analyzed by means of LC-MS/MS (SCIEX Triple Quad™ 7500 QTRAP® Ready). Zero time points were prepared by adding ice-cold methanol to the compound prior the addition of the plasma.

3.4. Microsomal stability

Microsomal stability assay was performed using the 0.5 mg/ml human pooled liver microsomal preparation (Thermo Scientific) and 10 µM compounds in 90 mM TRIS-Cl buffer pH 7.4 containing 2 mM NADPH and 2 mM $MgCl_2$ for 10, 30 and 45 min at 37°C. The reactions were terminated by the addition of four volumes of ice-cold methanol, mixed vigorously, frozen at -20°C for 1h and left overnight at 8°C. After that, the samples were centrifuged and the supernatants were analyzed by means of LC-MS/MS (SCIEX Triple Quad™ 7500 QTRAP® Ready). Zero time points were prepared by adding ice-cold methanol to the mixture of compound with cofactors prior to the addition of microsomes. The microsomal half-lives ($t_{1/2}$) were calculated using the equation $t_{1/2}$=0.693/k, where k is the slope found in the linear fit of the natural logarithm of the fraction remaining of the parent compound vs. incubation time. Intrinsic clearance (CL_{int}) was calculated using the following formula:

$$CL_{int} = V * \frac{ln2}{t_{1/2}}$$

where V = incubation volume per milligram of microsomal protein (µL/mg) and $t_{1/2}$ = microsomal half-life.

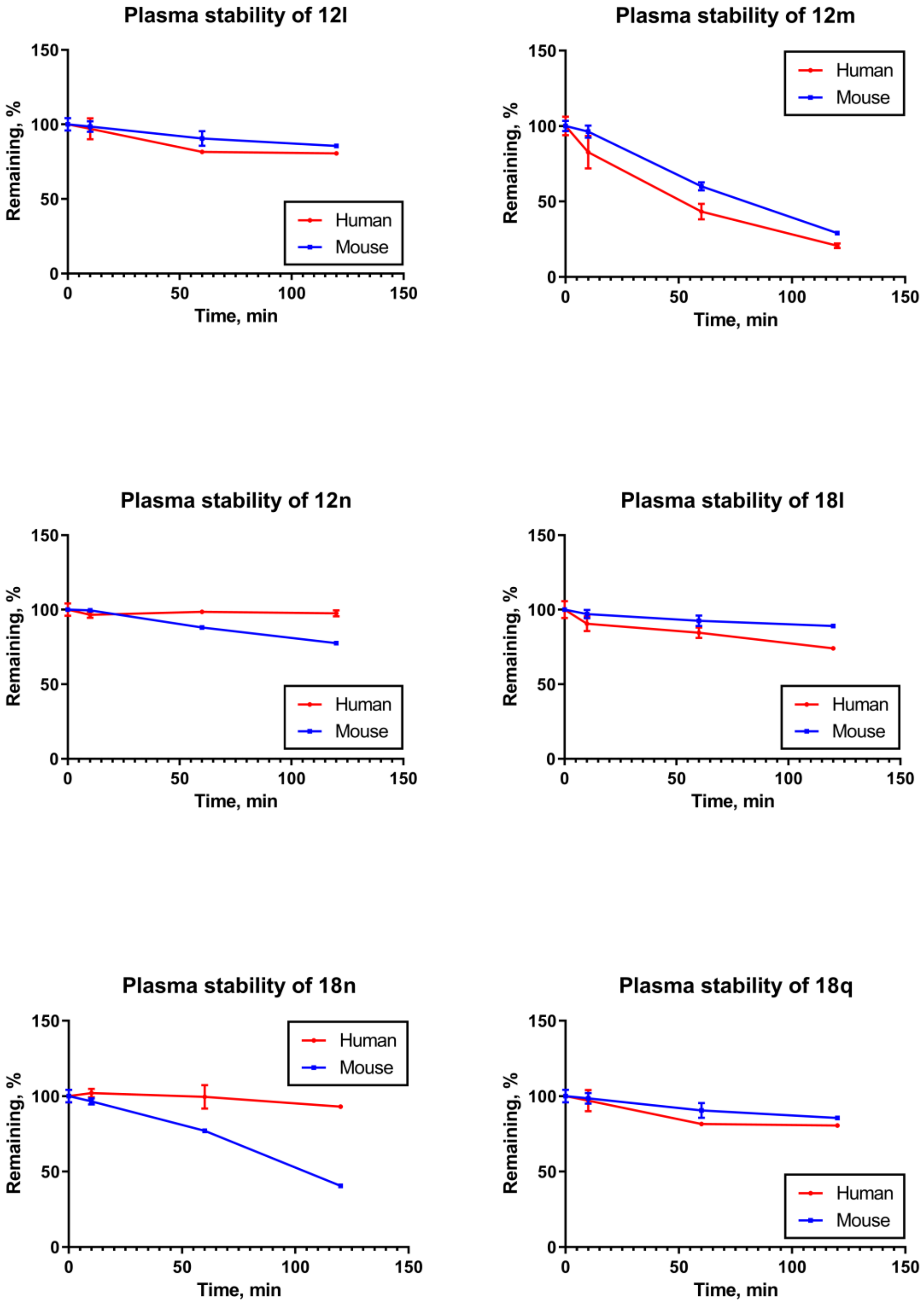

Figure S2. Stability of **12l**, **12m**, **12n**, **18l**, **18n**, and **18q** in human and mouse plasma.

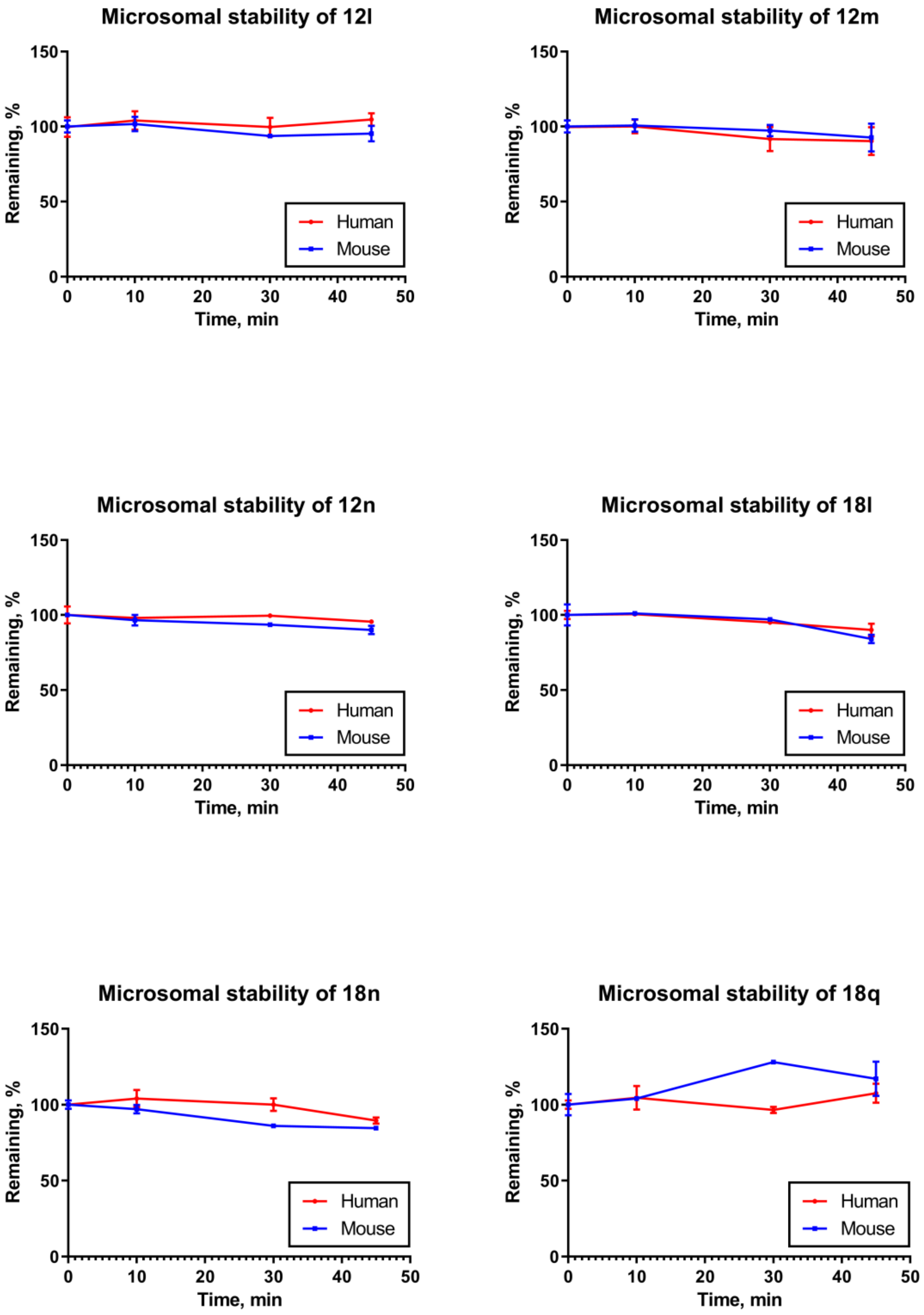

Figure S3. Stability of **12l**, **12m**, **12n**, **18l**, **18n**, and **18q** in human and mouse liver microsomes.

3.5. Caco-2 permeability assay

Transepithelial bi-directional transport of compounds (10 µM, pH 7.4/7.4) was tested on 21-day differentiated Caco-2 monolayers. Briefly, the cells were seeded at a density of 67,500 cells per 96-well plate insert (Millicell®, Merck KGaA, Darmstadt, Germany). They were left to differentiate for 21 days with medium exchange every 2–3 days. Prior to the experiment, the medium from both apical and basolateral well was exchanged for HBSS buffer (containing 10 mM HEPES and 25 mM glucose) and left to equilibrate for 30 min. Compound permeability was assayed by replacing pure HBSS with HBSS containing 10 µM compounds and 220 µM Lucifer yellow (LY) solution in an apical insert (A to B transport) or in a basolateral well (B to A transport). The treated cells were then returned to the incubator for 2 h at 37 °C. After that, samples were collected from both apical inserts and basolateral wells and analyzed using LC-MS/MS (SCIEX Triple Quad™ 7500 QTRAP® Ready). LC separation was done on Synergi 4µm Fusion 50 × 2 mm column (Phenomenex) using a water/acetonitrile gradient with 0.1% formic acid starting from 2 to 98% for 4.5 min. The apparent permeability coefficient (Papp) was calculated from the following equation: Papp = (dQ / dt) / C0 * A, where dQ/dt is the rate of absorption of the drug across the cells, C0 is the donor compartment concentration at time zero, and A is the area of the monolayer. Efflux ratio was expressed as (Papp B-A) / (Papp A-B). Recovery (%) was determined as (total compound mass in donor and receiver compartments at the end of the incubation / initial compound mass in the donor compartment) x 100. The data represent means ± SD of two independent experiments performed in triplicate.

	**Papp (cm/s) A-B x 10^{-6}**	**Recovery (%)**	**Papp (cm/s) B-A x 10^{-6}**	**Recovery (%)**	**Efflux ratio**
**12l**	3.1 ± 1.3	85	10.7 ± 2.5	83	3.4
**12m**	8.0 ± 2.9	96	32.9 ± 5.3	130	4.1
**12n**	4.4 ± 1.8	79	2.2 ± 0.4	87	0.5
**18l**	10.1 ± 4.0	87	8.3 ± 0.5	101	0.8
**18n**	11.3 ± 3.7	148	7.3 ± 1.3	110	0.6
**18q**	2.0 ± 0.3	89	2.1 ± 0.4	101	1.0
**Digoxin**	1.7 ± 0.2	131	13.8 ± 0.9	77	8.5
**Atenolol**	0.3 ± 0.1	105	n.d.	n.d.	n.d.
**verapamil**	n.d.	n.d.	n.d.	n.d.	n.d.

Table S2. Results of Caco-2 permeability assay including recovery values. Digoxin, atenolol and verapamil were used as control compounds.

3.6. Antiviral and cytotoxicity assays

The anti-SARS-CoV-2 activity was assessed by evaluating the inhibition of virus-induced cytopathic effects (CPE) in both Vero E6 and Calu-3 cell line. Cells were cultured in DMEM containing 2% FBS, penicillin (100 U/mL), and streptomycin (100 µg/mL) (all Merck) in 384-well plates, with each well containing 5,000 Vero E6 cells or 15,000 Calu-3 cells. Test compounds were prepared in two-fold serial dilutions, starting from 50 µM using an ASSIST pipetting robot and applied to the cells in triplicate. After a 2-hour pretreatment at 37°C and 5% CO_2, cells were infected with SARS-CoV-2 (strain hCoV-19/Czech Republic/NRL_6632_2/2020) at MOIs of ~0.03 or ~0.01.

Following a three-day incubation at 37°C with 5% CO_2, cell viability was assessed using the XTT assay (Sigma-Aldrich). The production of orange-colored formazan by viable cells was quantified by measuring absorbance at 450 nm with an EnVision plate reader (Perkin Elmer). The effective concentration (EC_{50}) values, which reduce the viral CPE by 50%, were determined using nonlinear regression in GraphPad Prism 10.2.3.

To evaluate cytotoxicity (CC_{50}), the same serial dilutions of each compound were incubated with Vero E6 and Calu-3 cells without virus. The cytotoxic concentration (CC_{50}) values, which reduce the cell viability by 50%, were determined as above. The standard deviation (SD) was calculated from the 95 % confidence interval and the sample size using the following formula: SD = [(upper limit – lower limit) / 3.92] × √N.

**Compound**	**EC_{50} [µM]**	**95 % CI [µM]**	**SD [µM]**
**18l**	12	7.3-20	5.6
**18n**	9.8	7.3-13	2.5

Table S3. Antiviral effect of compounds **18l** and **18n** against SARS-CoV-2 in Calu-3 cell line and the corresponding 95 % confidence interval.

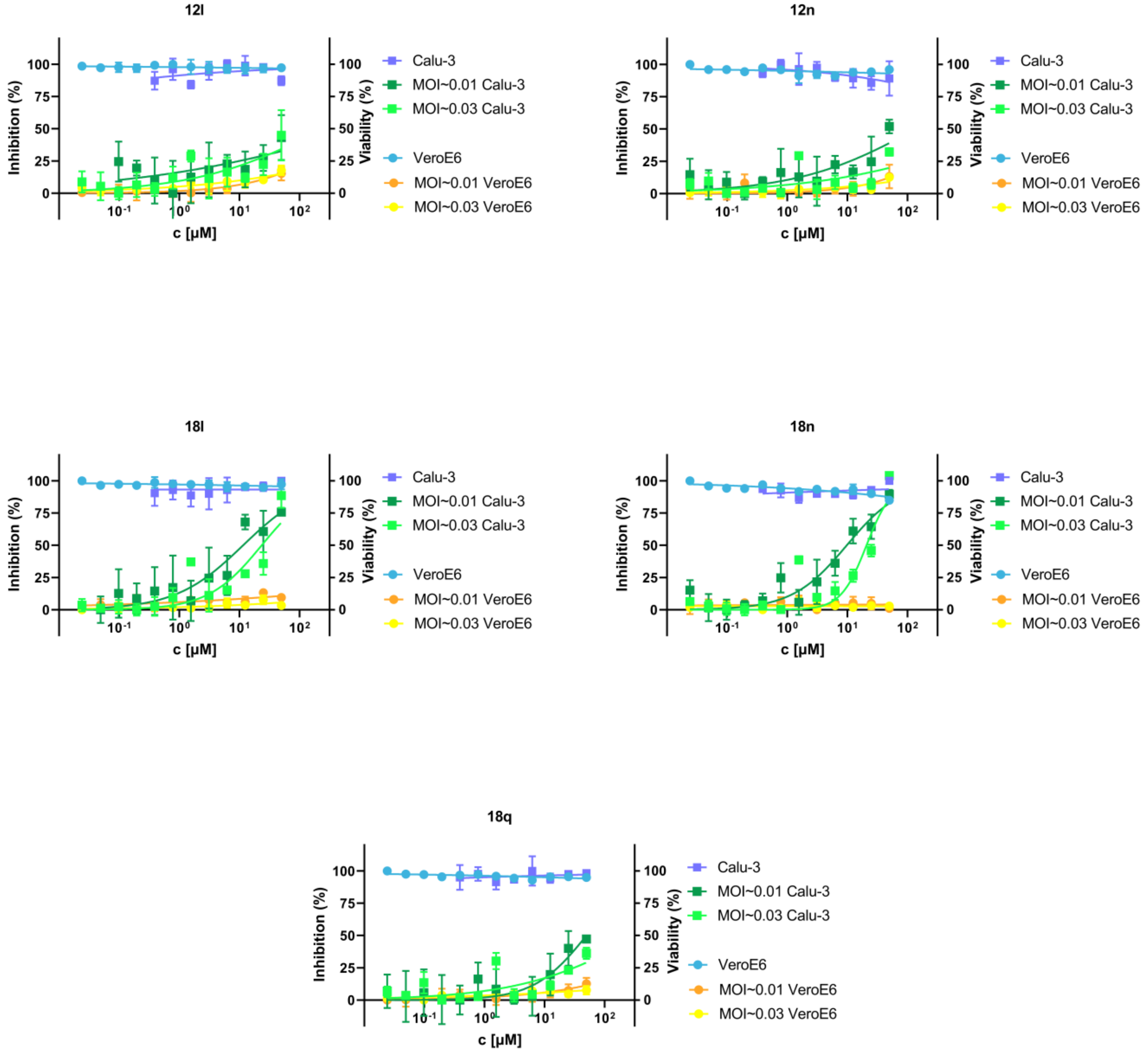

Figure S4. Antiviral effect of compounds **12l**, **12n**, **18l**, **18n** and **18q** against SARS-CoV-2 in Calu-3 (dark and light green lines) and in Vero E6 (yellow and orange lines) cell lines. The cells viability without virus in the presence of compounds in Calu-3 and Vero E6 cell lines are shown by violet and blue lines, respectively.

4. ^{1}H, ^{13}C, and ^{19}F NMR spectra of final compounds

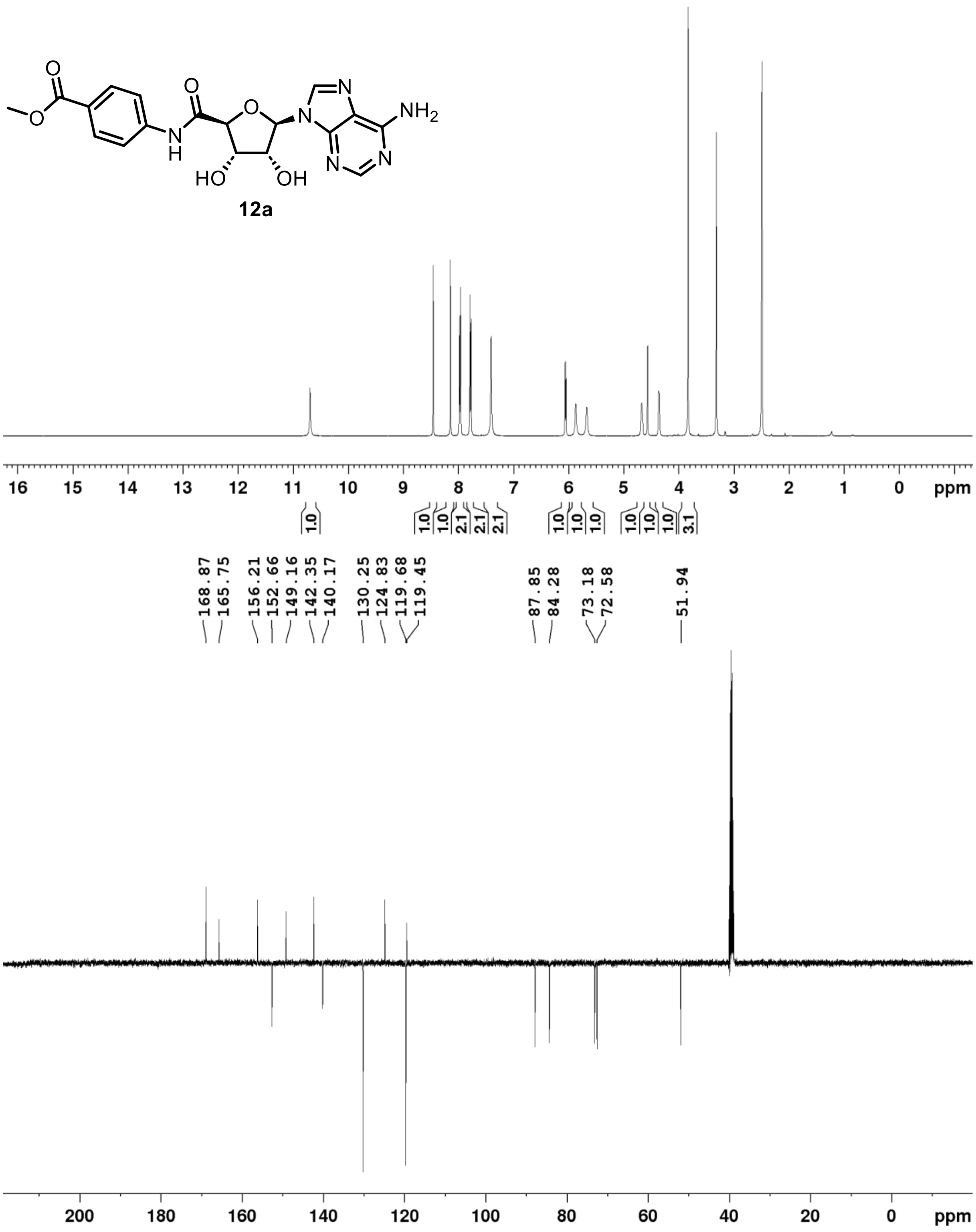

Figure S5. ^{1}H (top) and ^{13}C APT (bottom) NMR spectra of **12a** measured in DMSO-d_6.

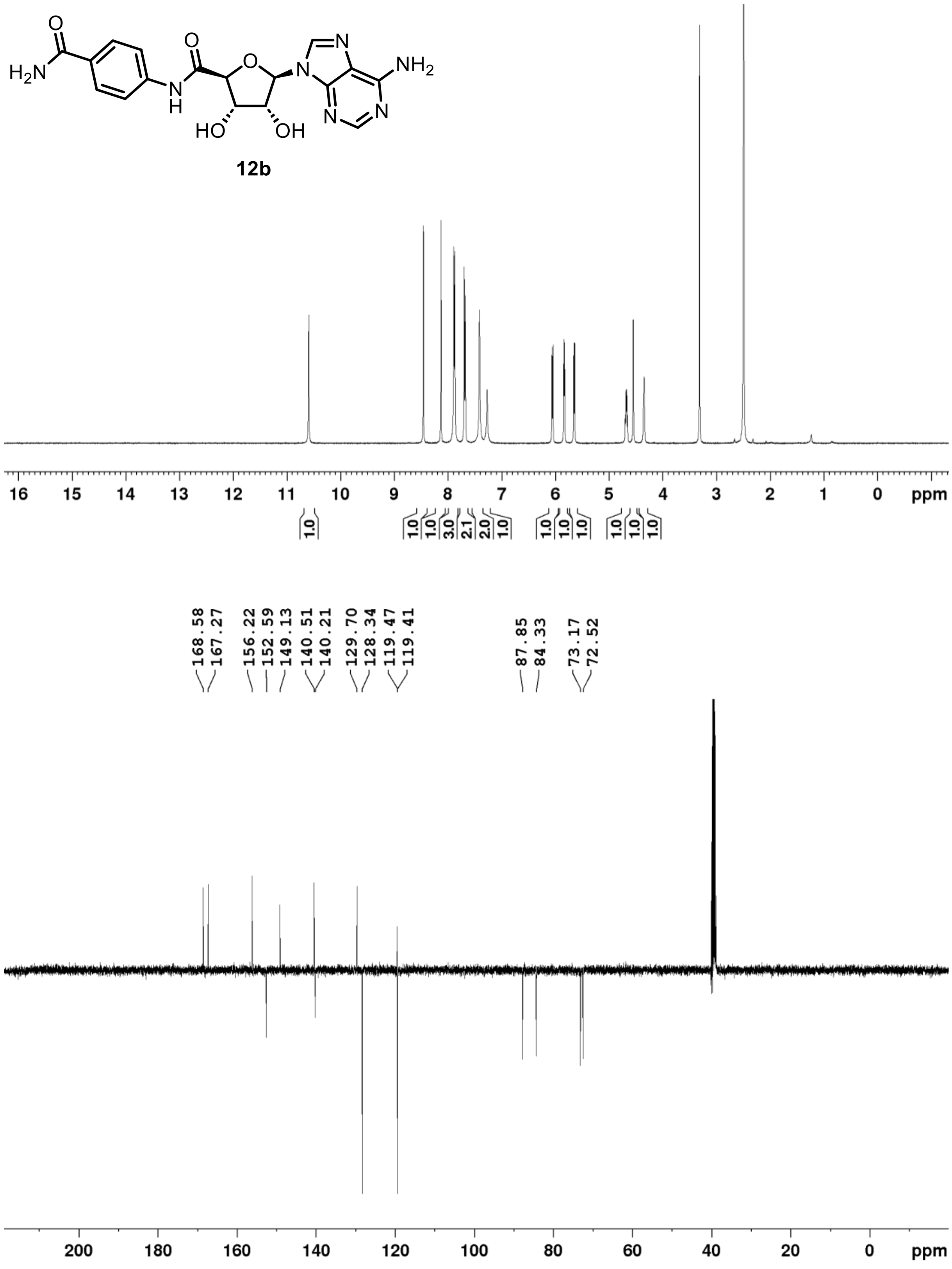

Figure S6. ^{1}H (top) and ^{13}C APT (bottom) NMR spectra of **12b** measured in DMSO-d_6.

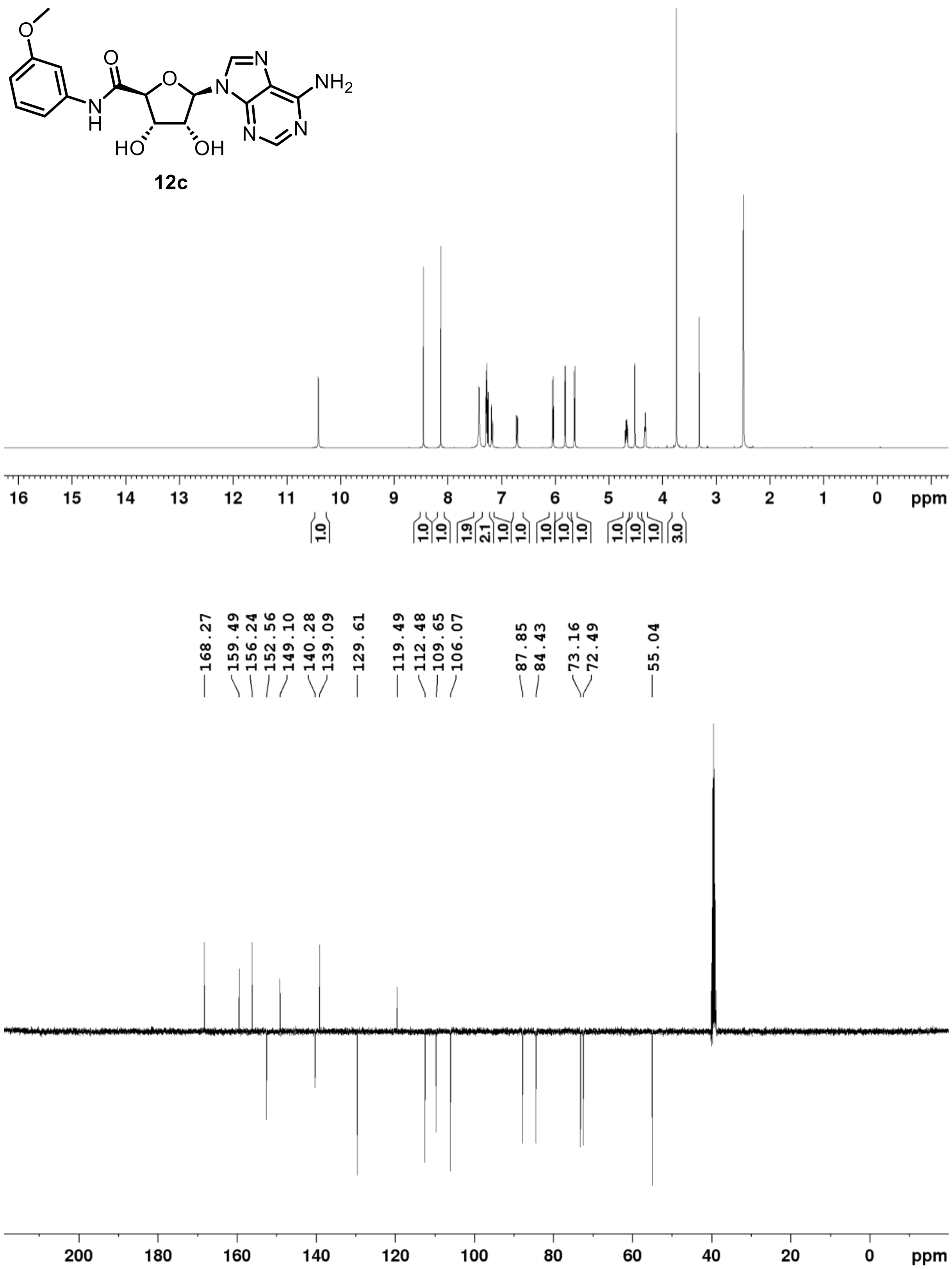

Figure S7. ^{1}H (top) and ^{13}C APT (bottom) NMR spectra of **12c** measured in DMSO-d_6.

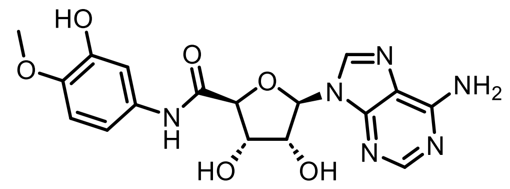

12d

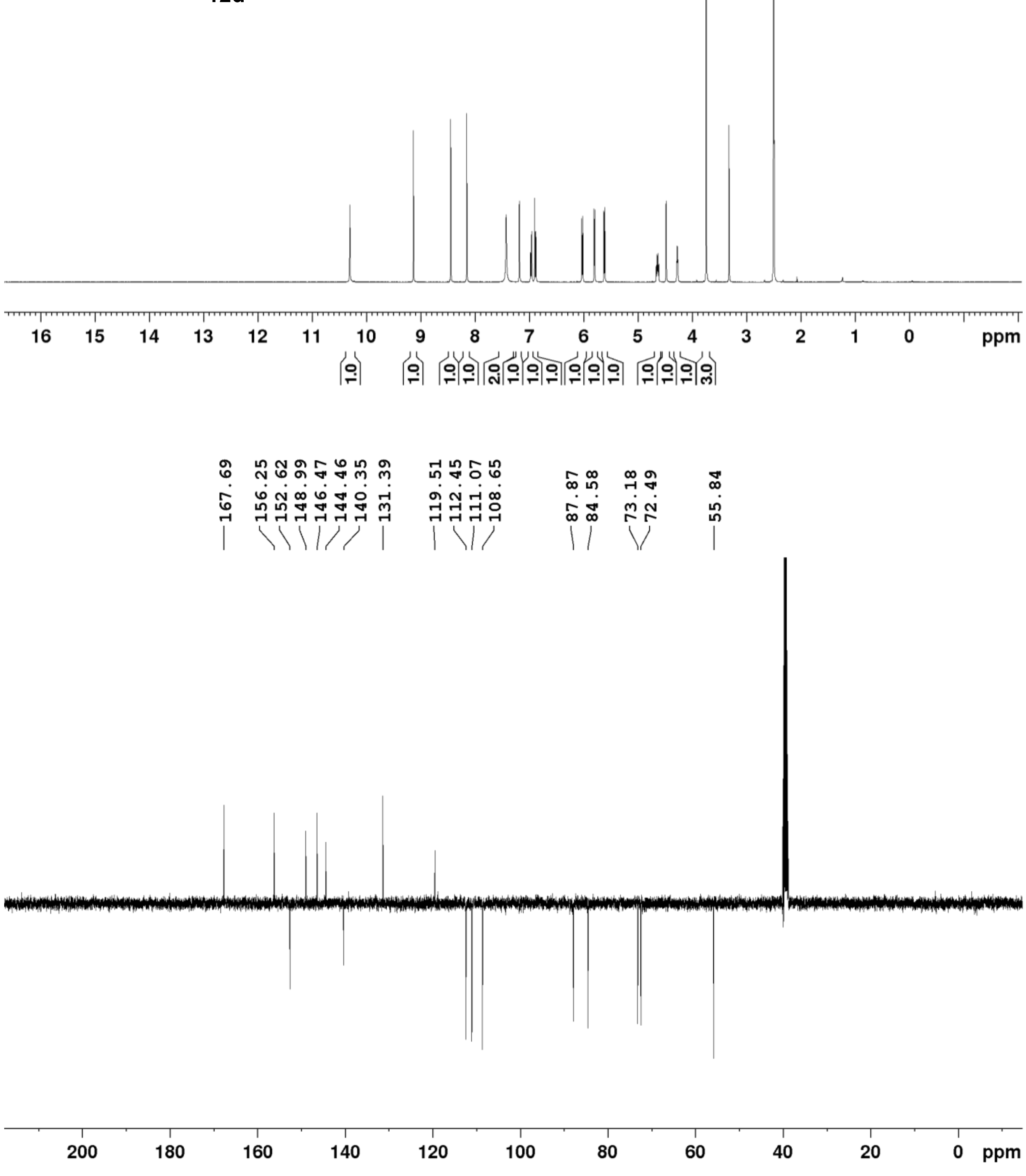

Figure S8. ^{1}H (top) and ^{13}C APT (bottom) NMR spectra of **12d** measured in DMSO-d_6.

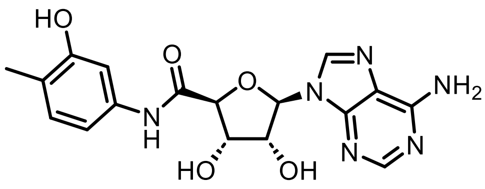

12e

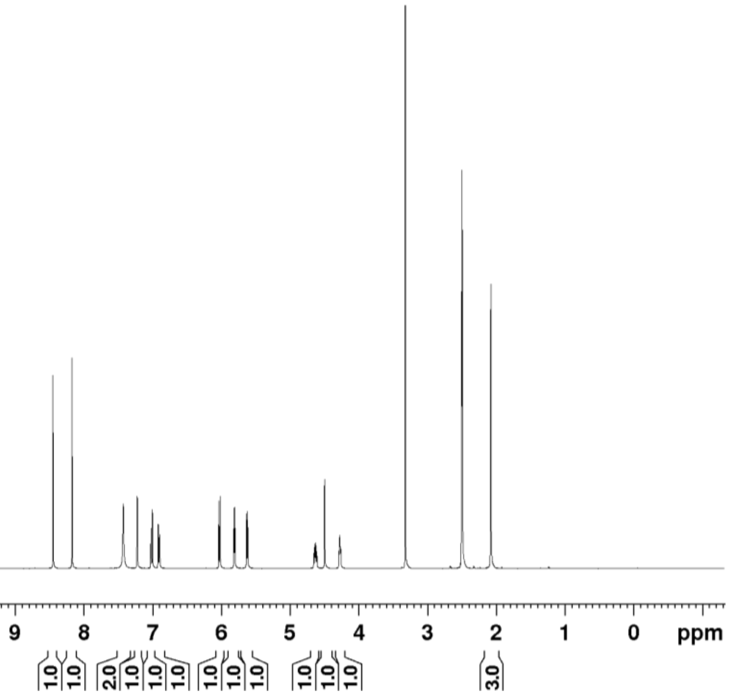

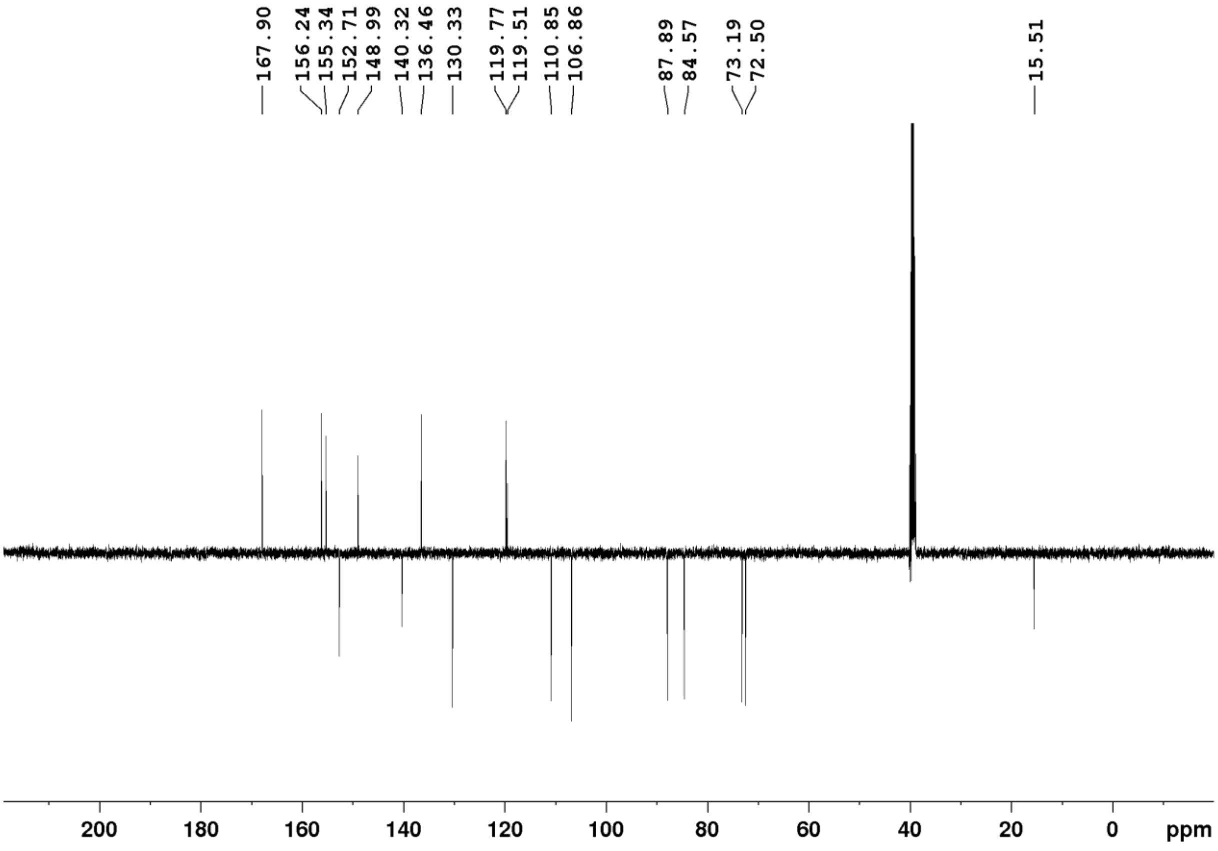

Figure S9. ^{1}H (top) and ^{13}C APT (bottom) NMR spectra of **12e** measured in DMSO-d_6.

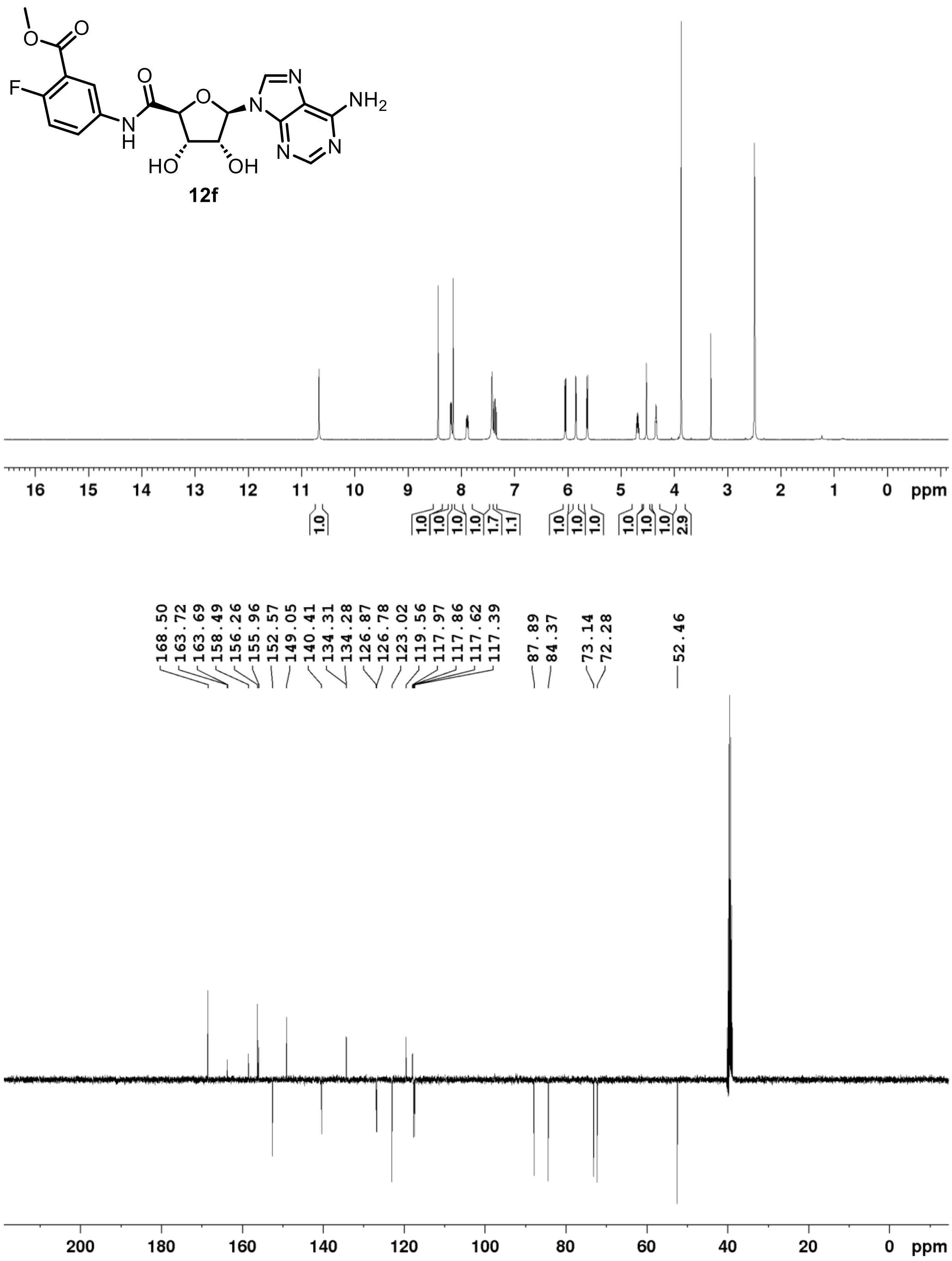

Figure S10. ^{1}H (top) and ^{13}C APT (bottom) NMR spectra of **12f** measured in DMSO-d_6.

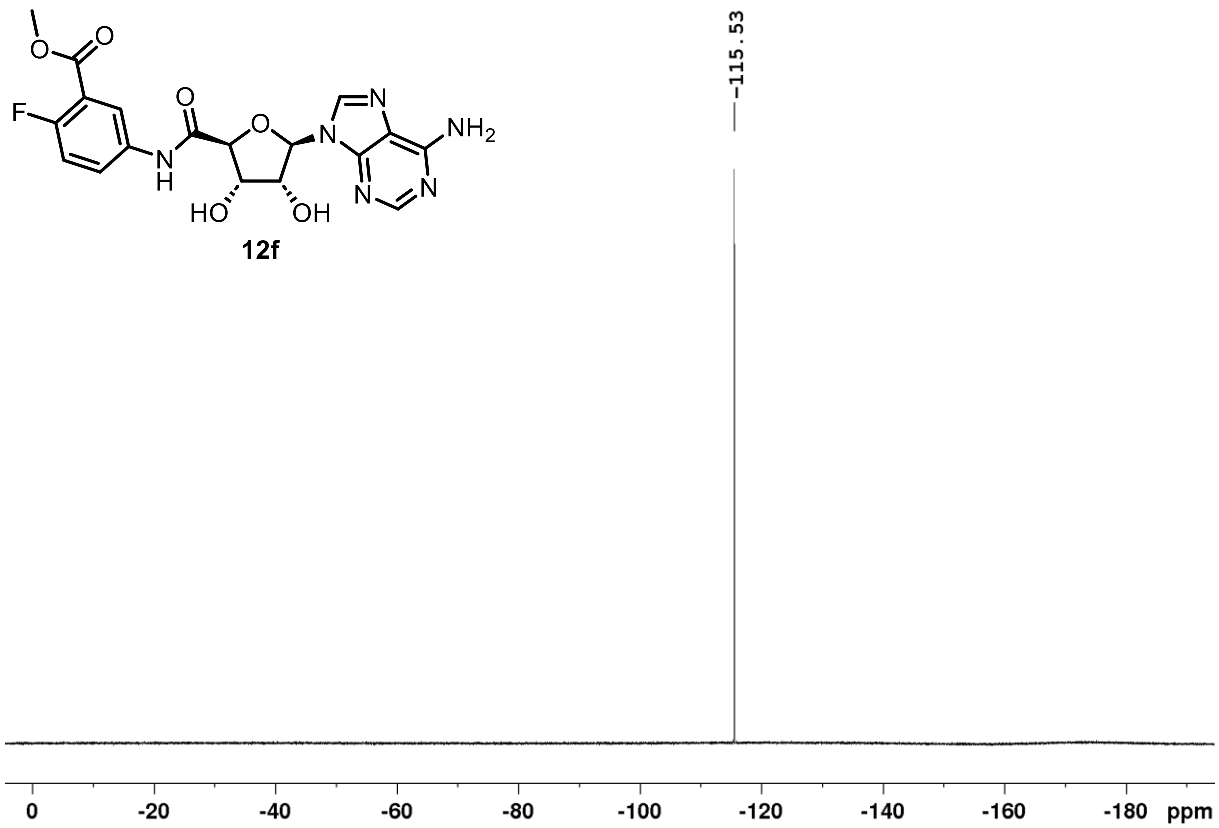

Figure S11. ^{19}F NMR spectra of **12f** measured in DMSO-d_6.

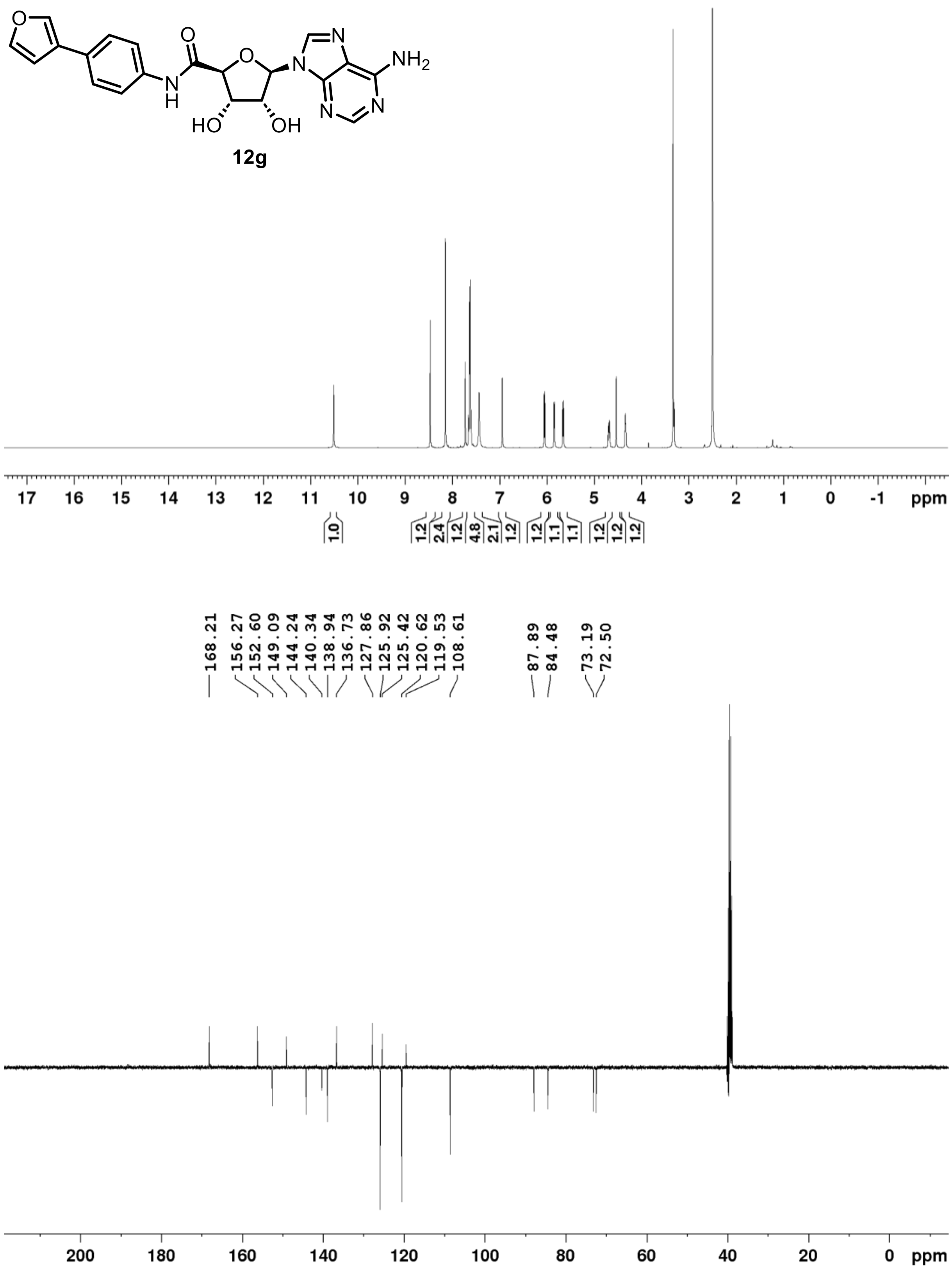

Figure S12. ^{1}H (top) and ^{13}C APT (bottom) NMR spectra of **12g** measured in DMSO-d_6.

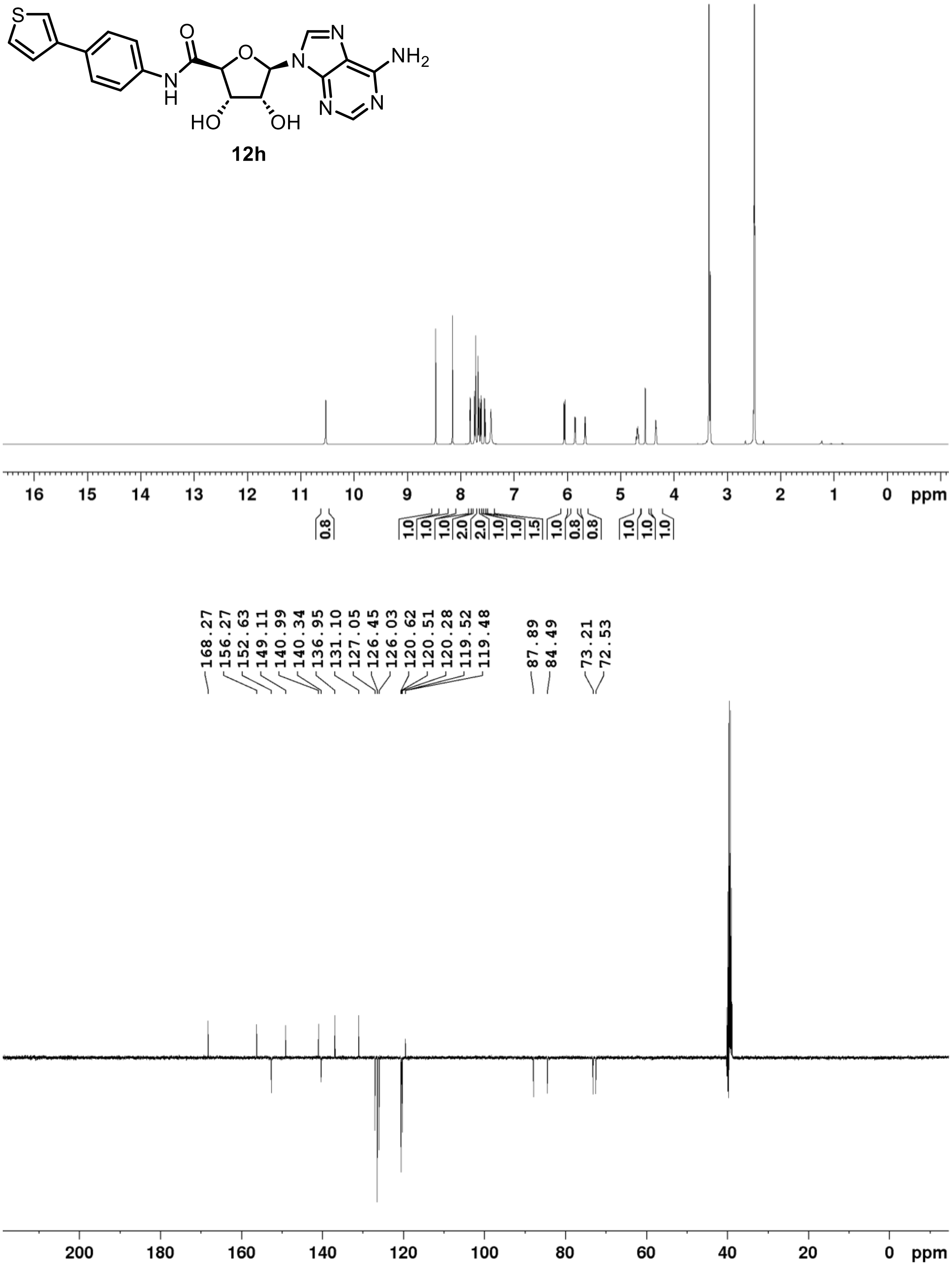

Figure S13. ^{1}H (top) and ^{13}C APT (bottom) NMR spectra of **12h** measured in DMSO-*d₆*.

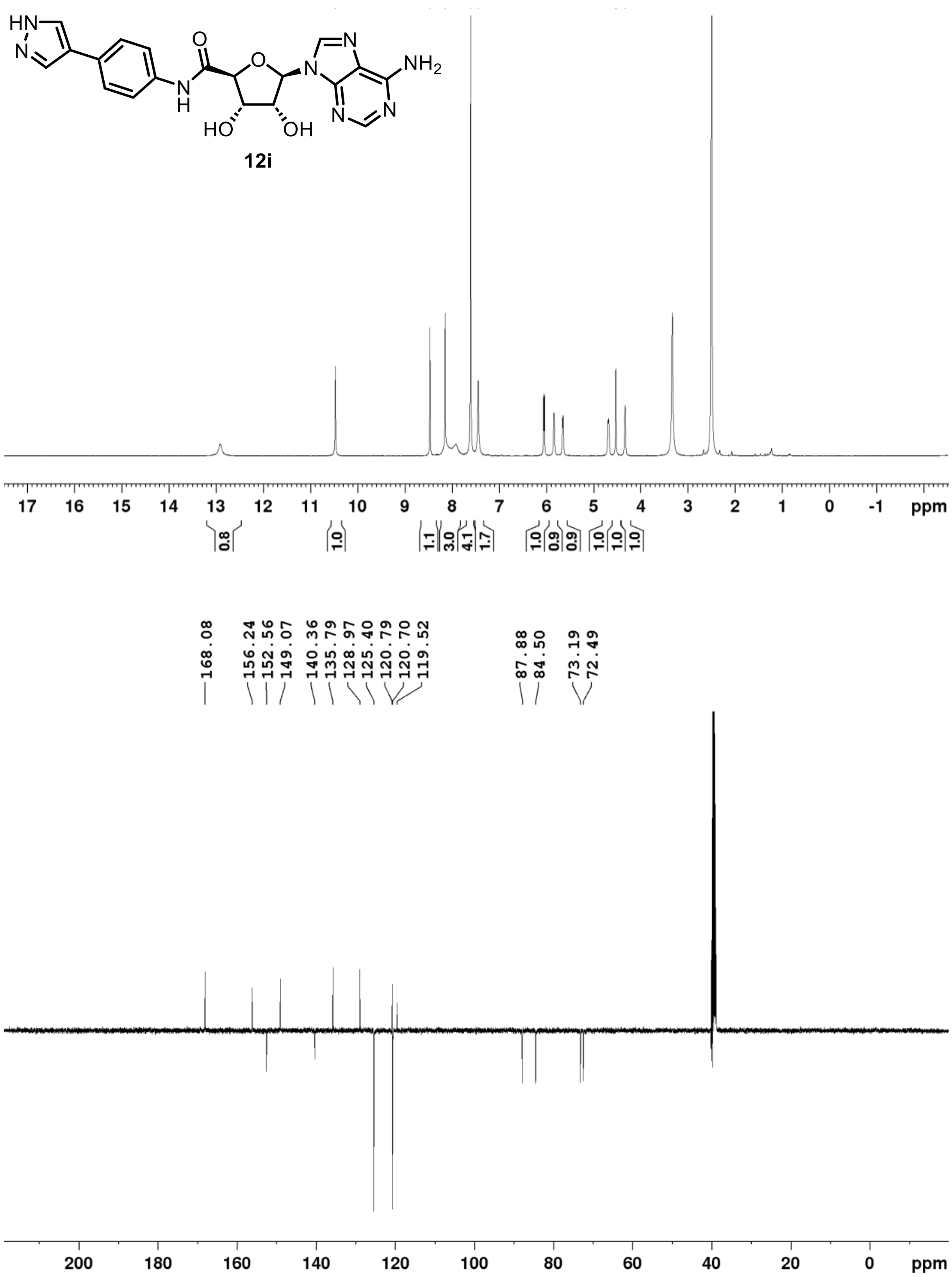

Figure S14. ^{1}H (top) and ^{13}C APT (bottom) NMR spectra of **12i** measured in DMSO-d_6.

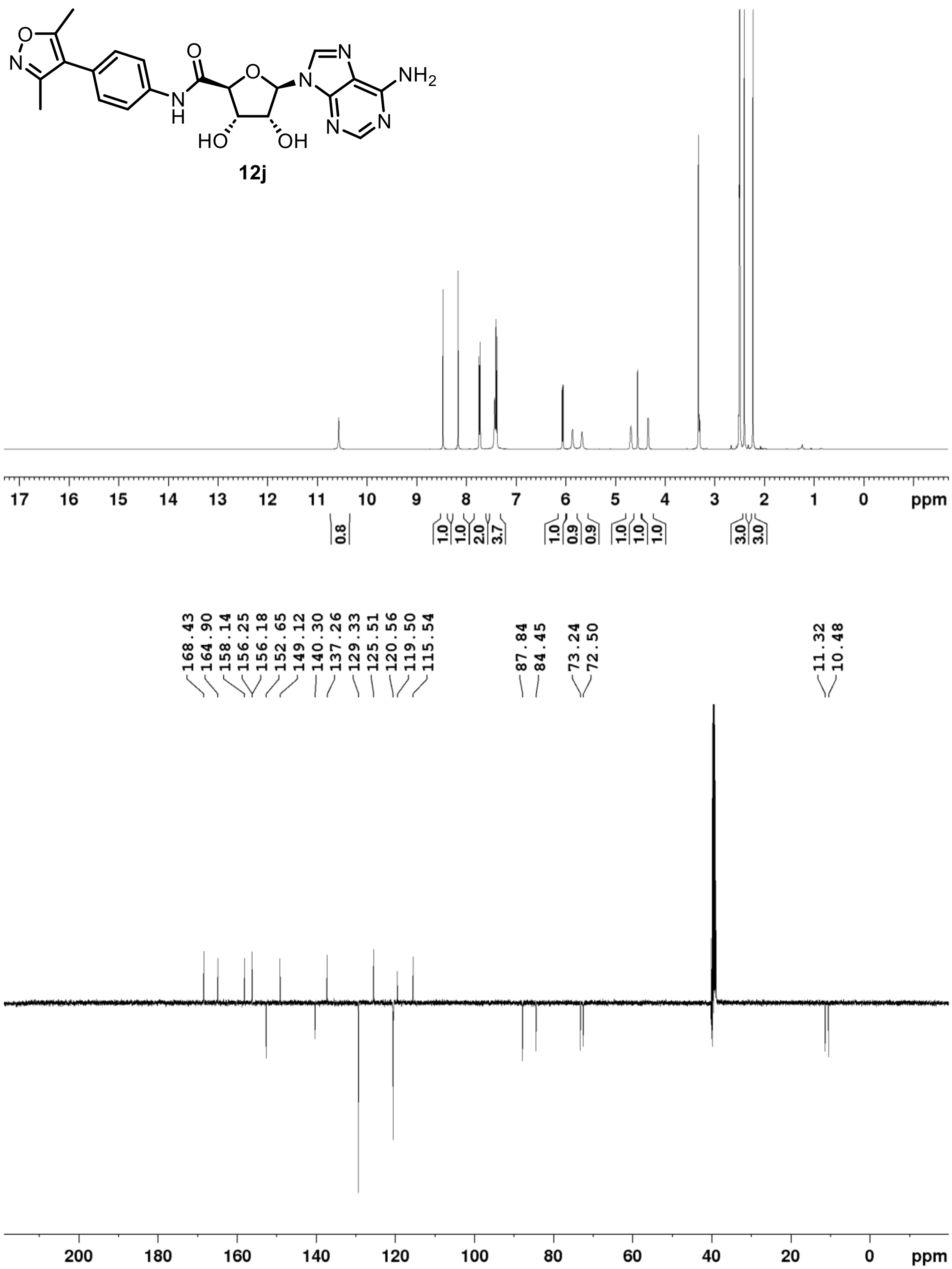

Figure S15. ^{1}H (top) and ^{13}C APT (bottom) NMR spectra of **12j** measured in DMSO-d_6.

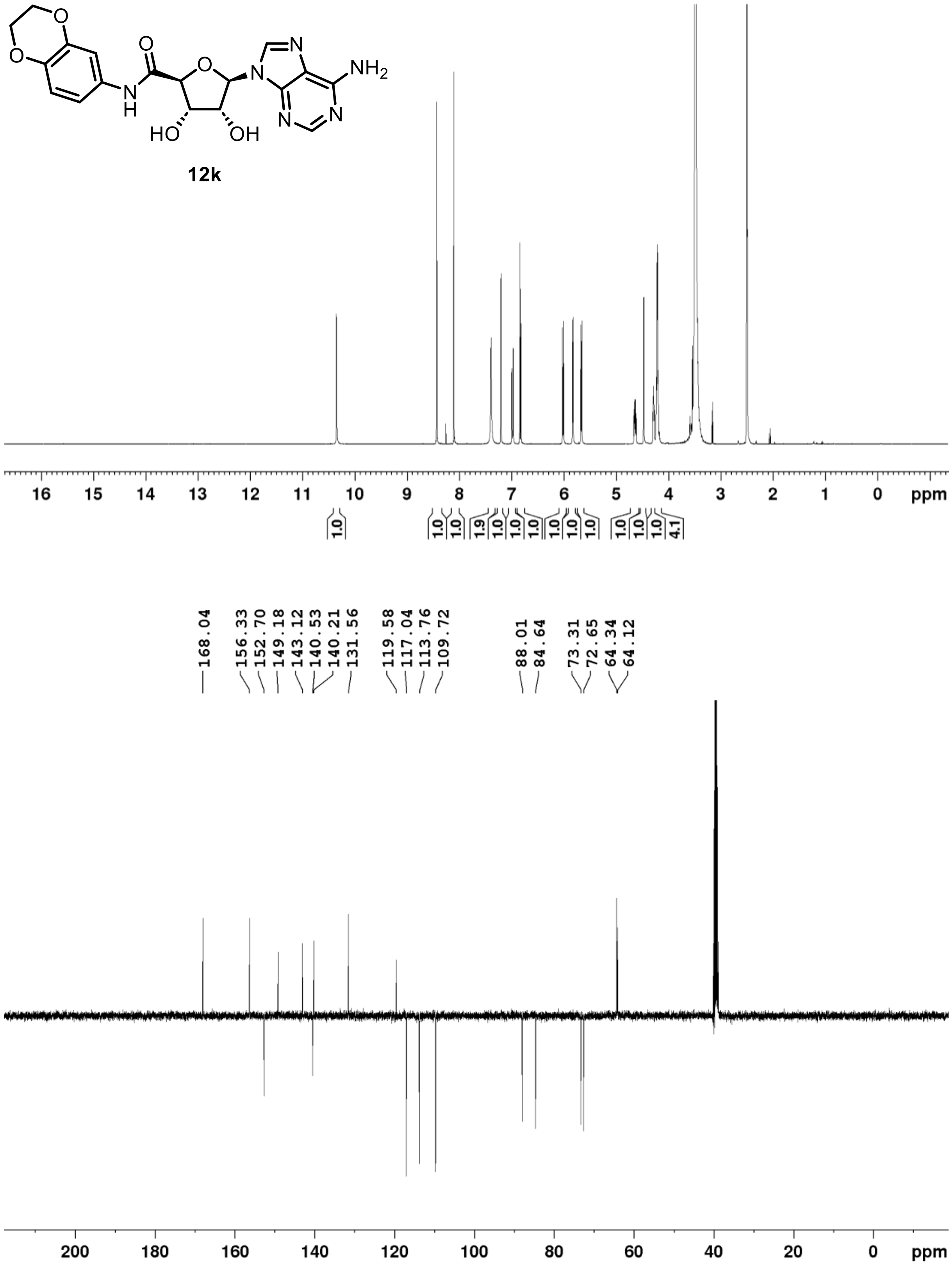

Figure S16. ^{1}H (top) and ^{13}C APT (bottom) NMR spectra of **12k** measured in DMSO-d_6.

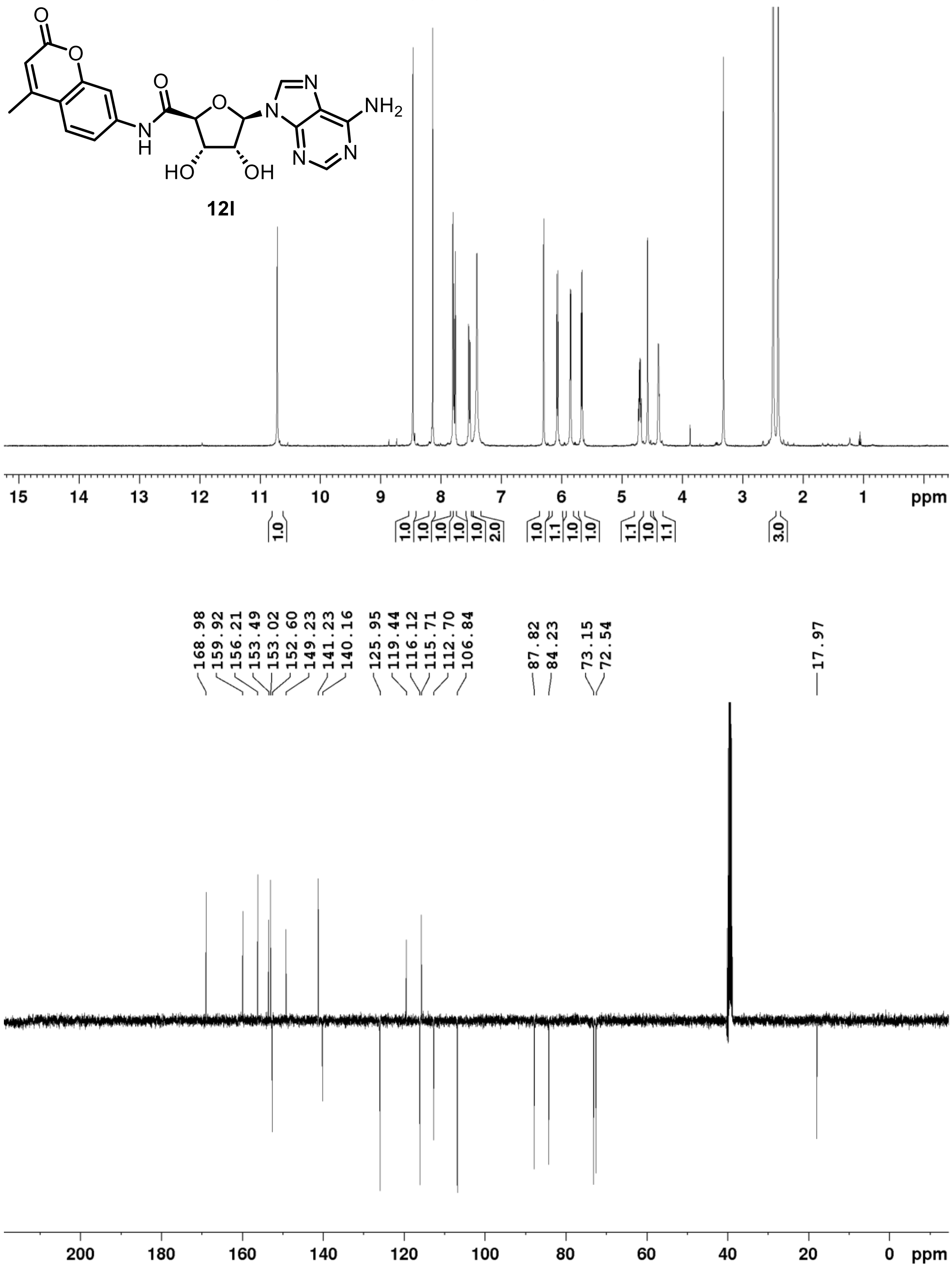

Figure S17. ^{1}H (top) and ^{13}C APT (bottom) NMR spectra of **12l** measured in DMSO-d_6.

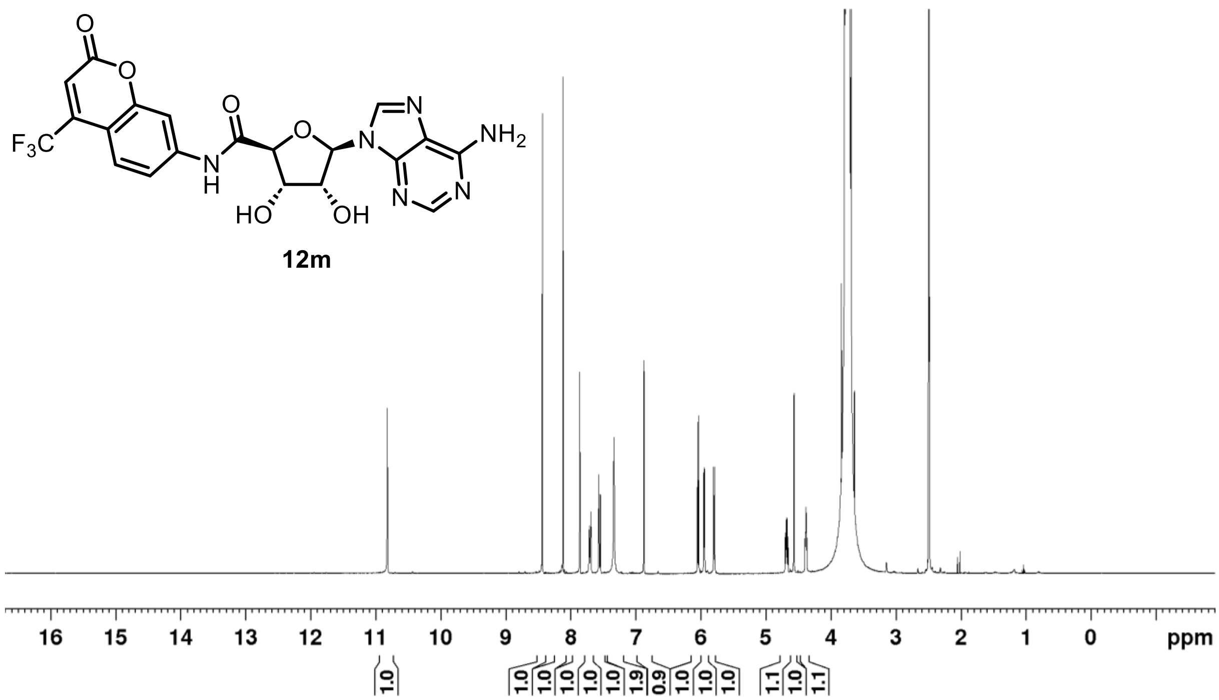

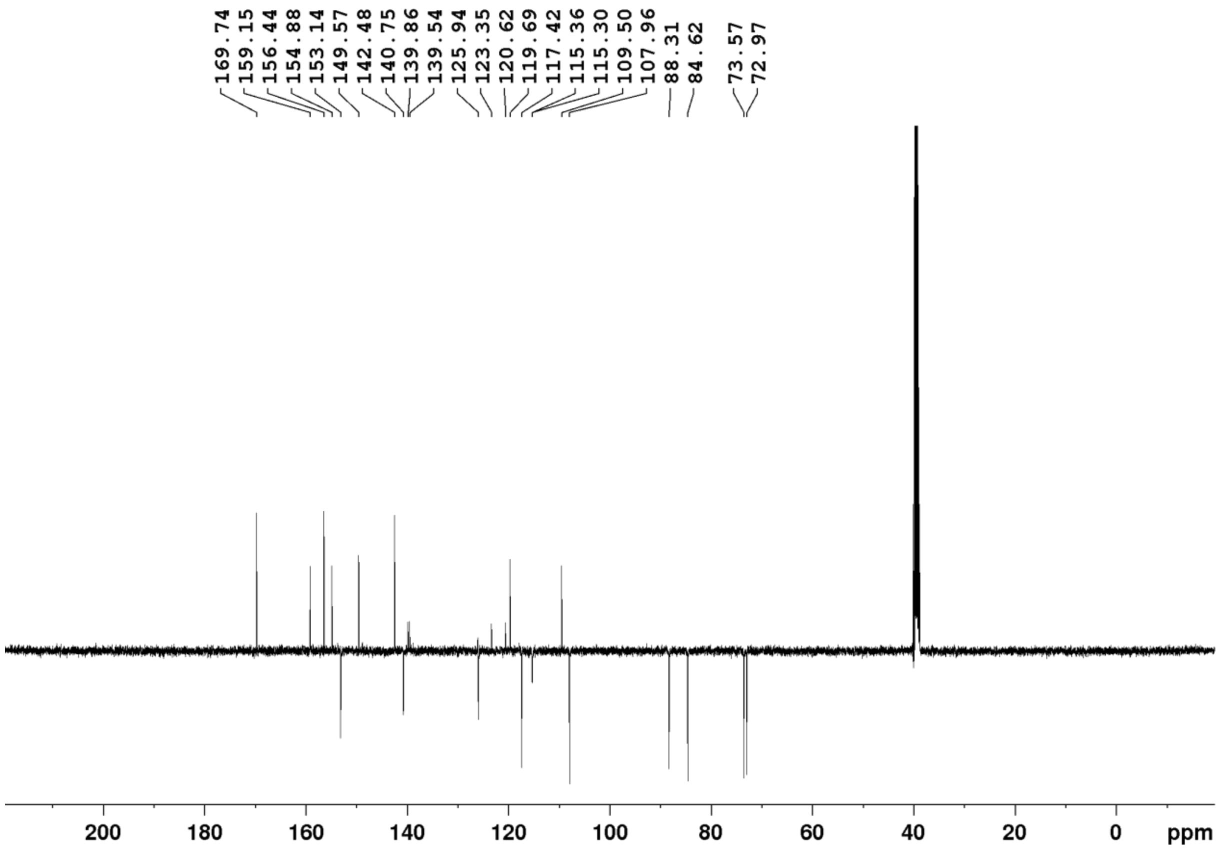

Figure S18. ^{1}H (top) and ^{13}C APT (bottom) NMR spectra of **12m** measured in DMSO-d_6.

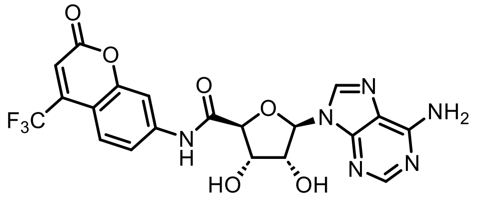

12m

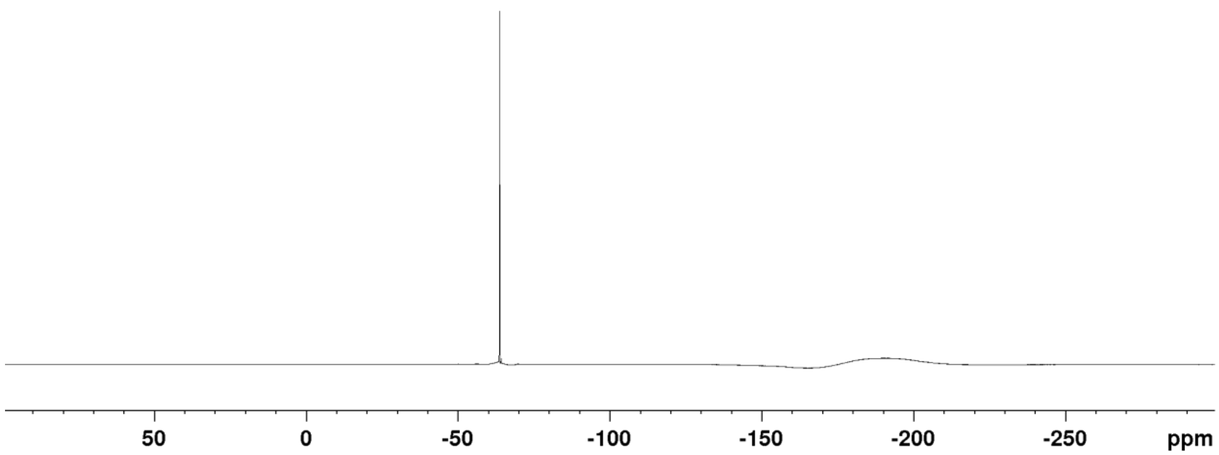

Figure S19. ^{19}F NMR spectra of **12m** measured in DMSO-d_6.

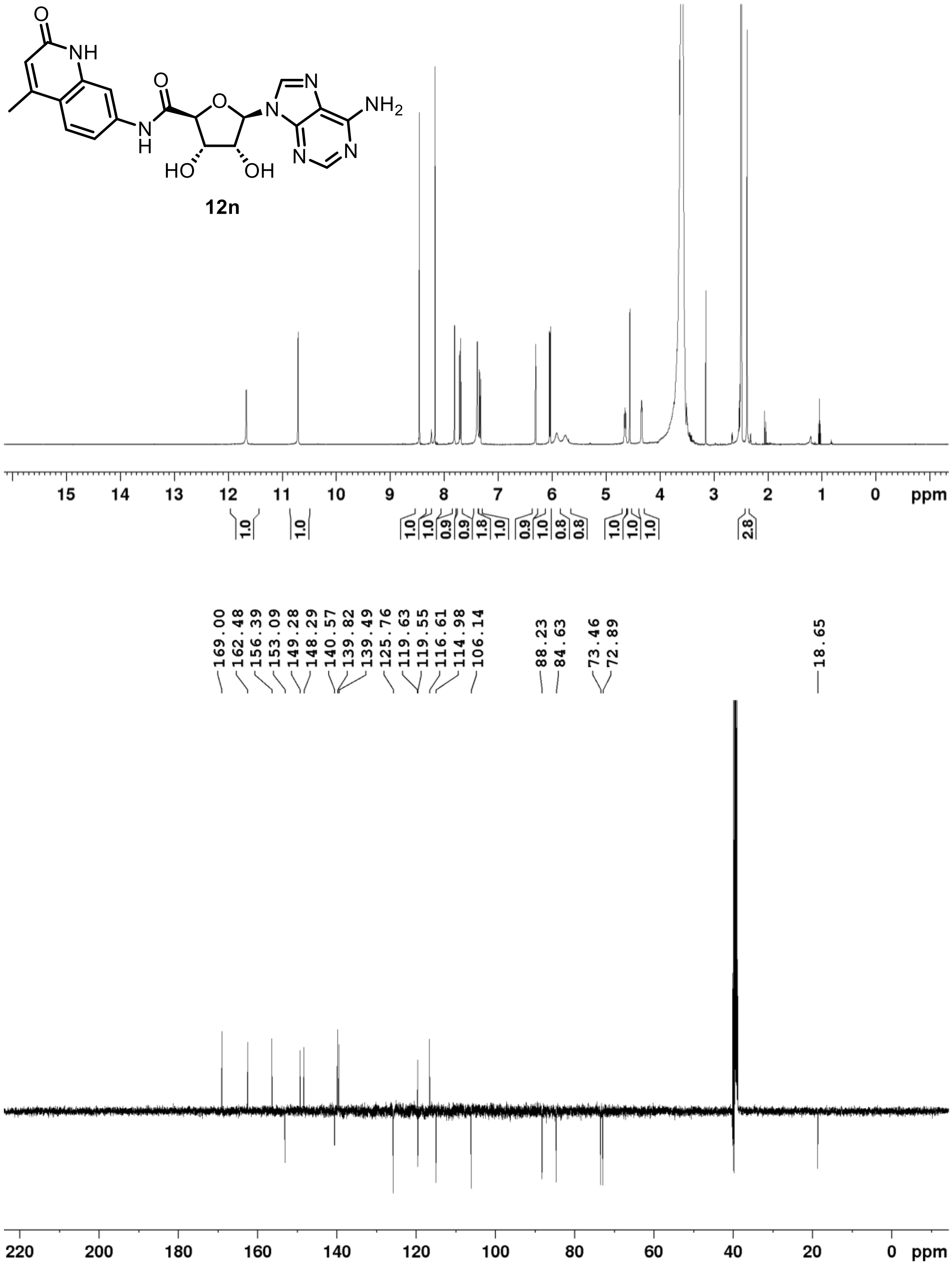

Figure S20. ^{1}H (top) and ^{13}C APT (bottom) NMR spectra of **12n** measured in DMSO-d_6.

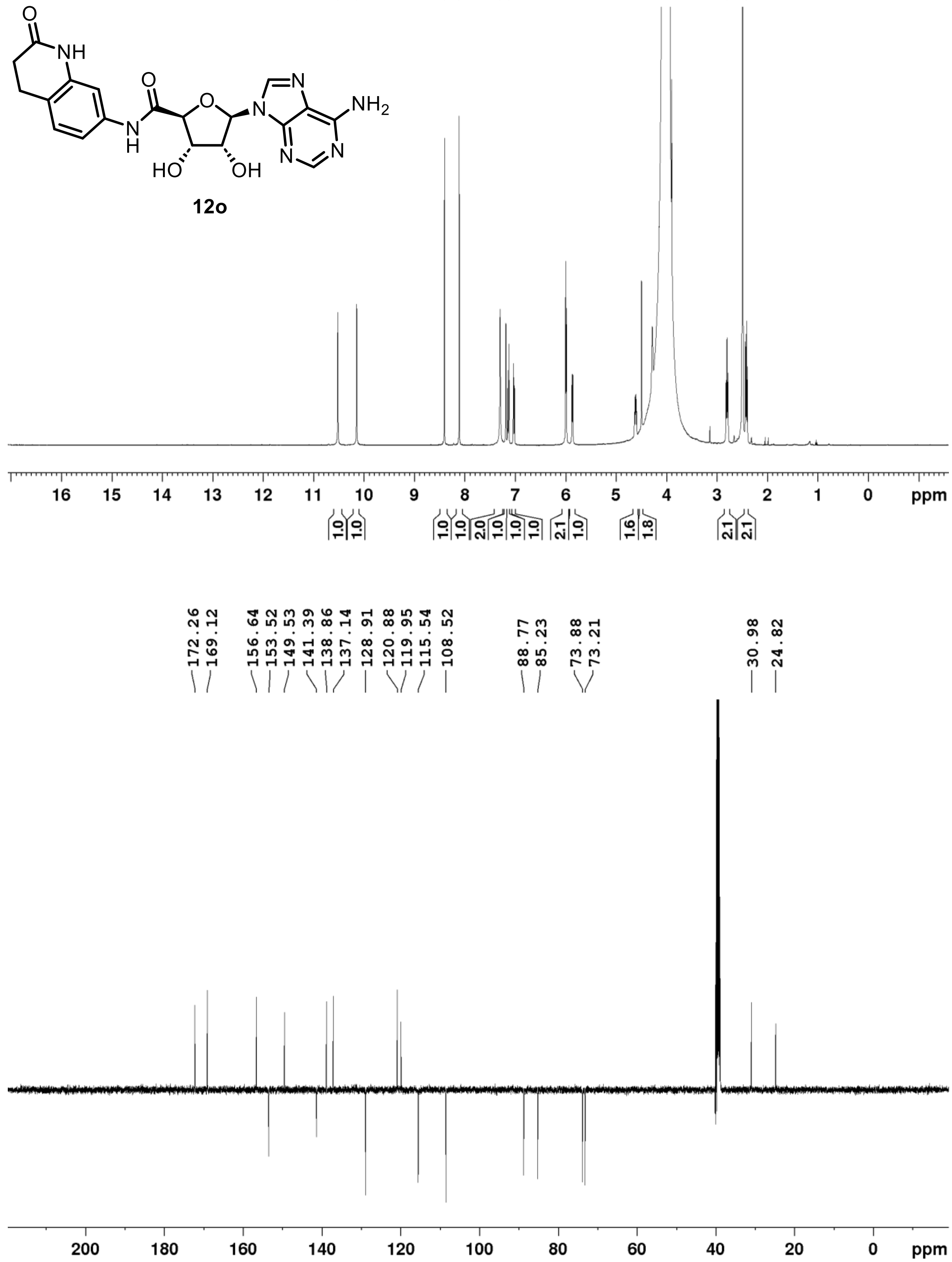

Figure S21. ^{1}H (top) and ^{13}C APT (bottom) NMR spectra of **12o** measured in DMSO-d_6.

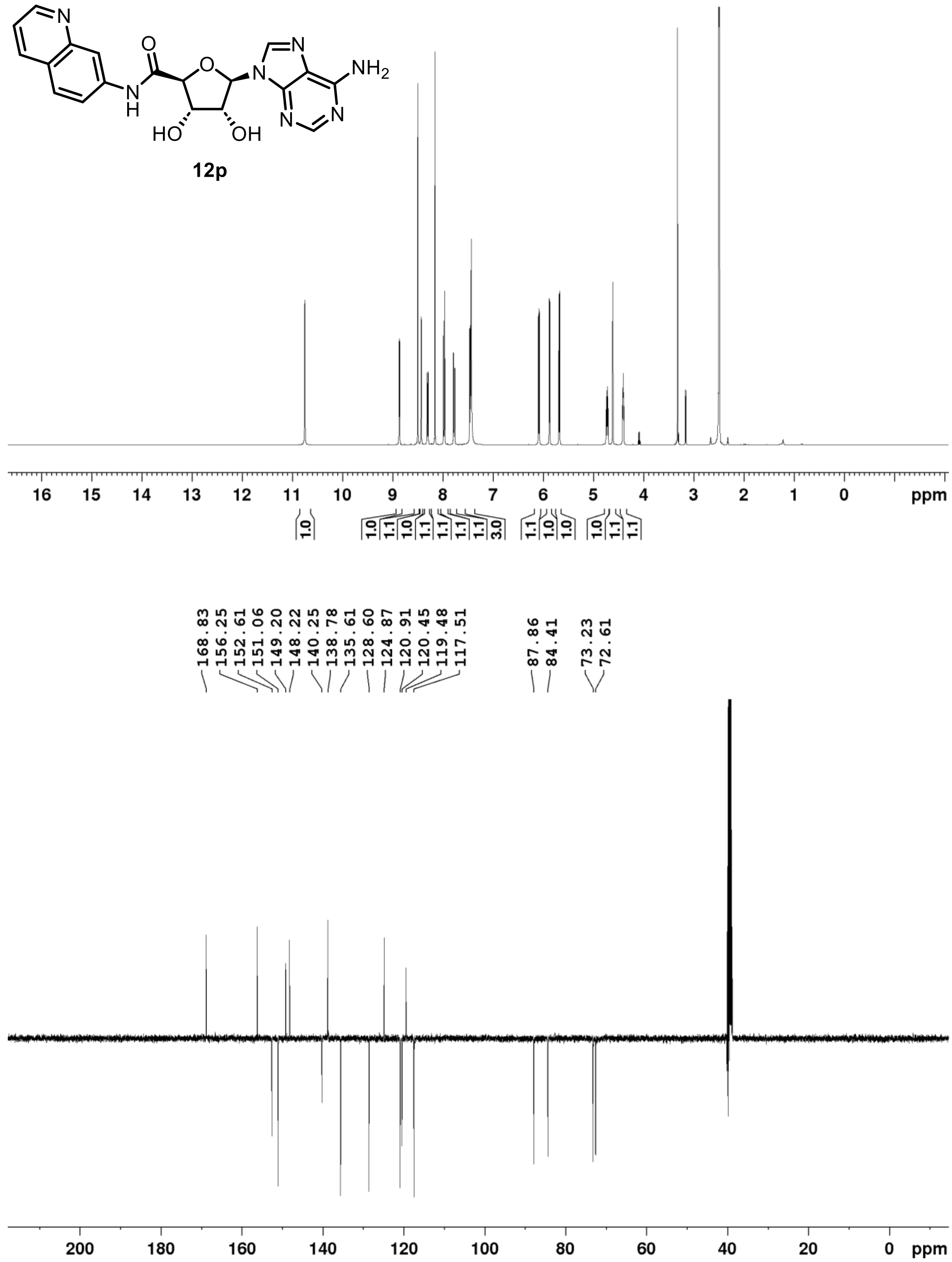

Figure S22. ^{1}H (top) and ^{13}C APT (bottom) NMR spectra of **12p** measured in DMSO-d_6.

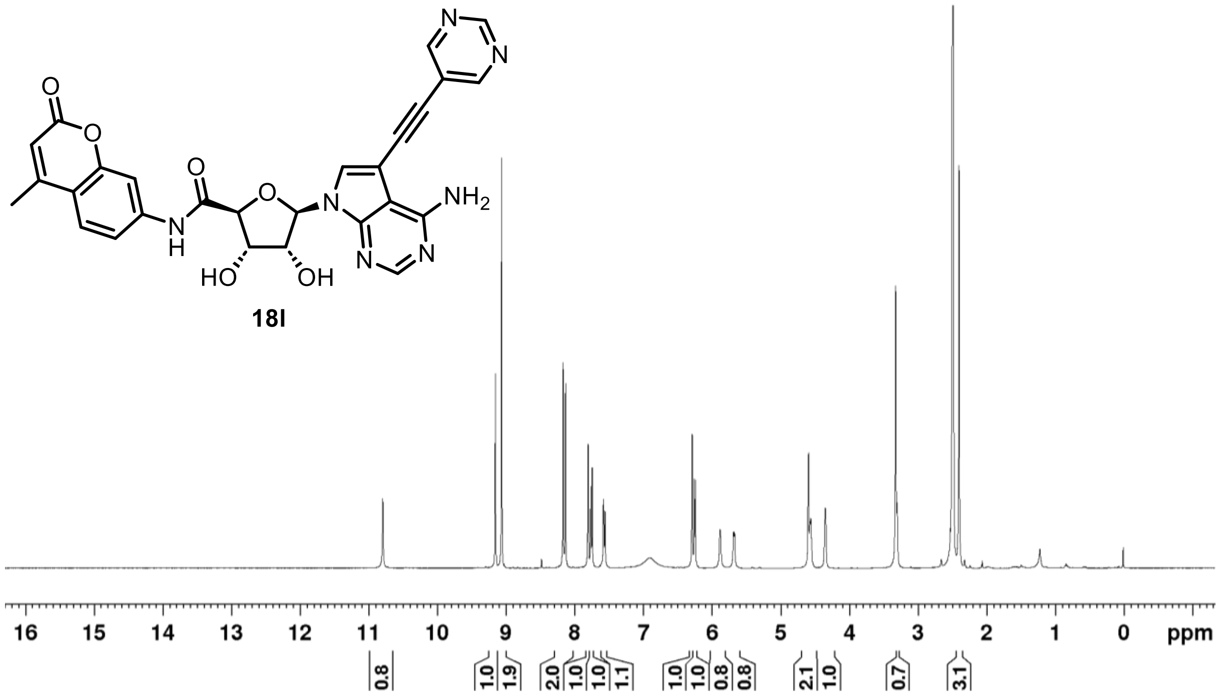

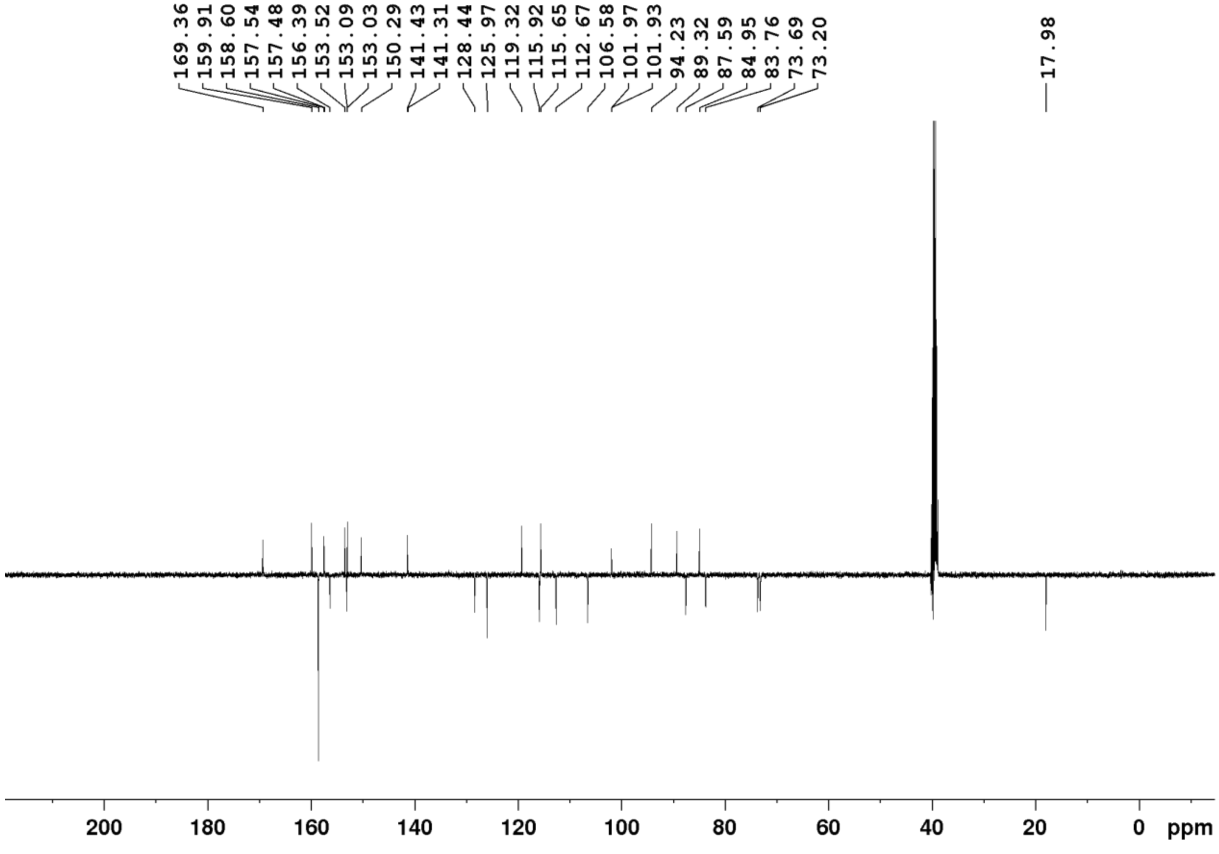

Figure S23. ^{1}H (top) and ^{13}C APT (bottom) NMR spectra of **18l** measured in DMSO-d_6.

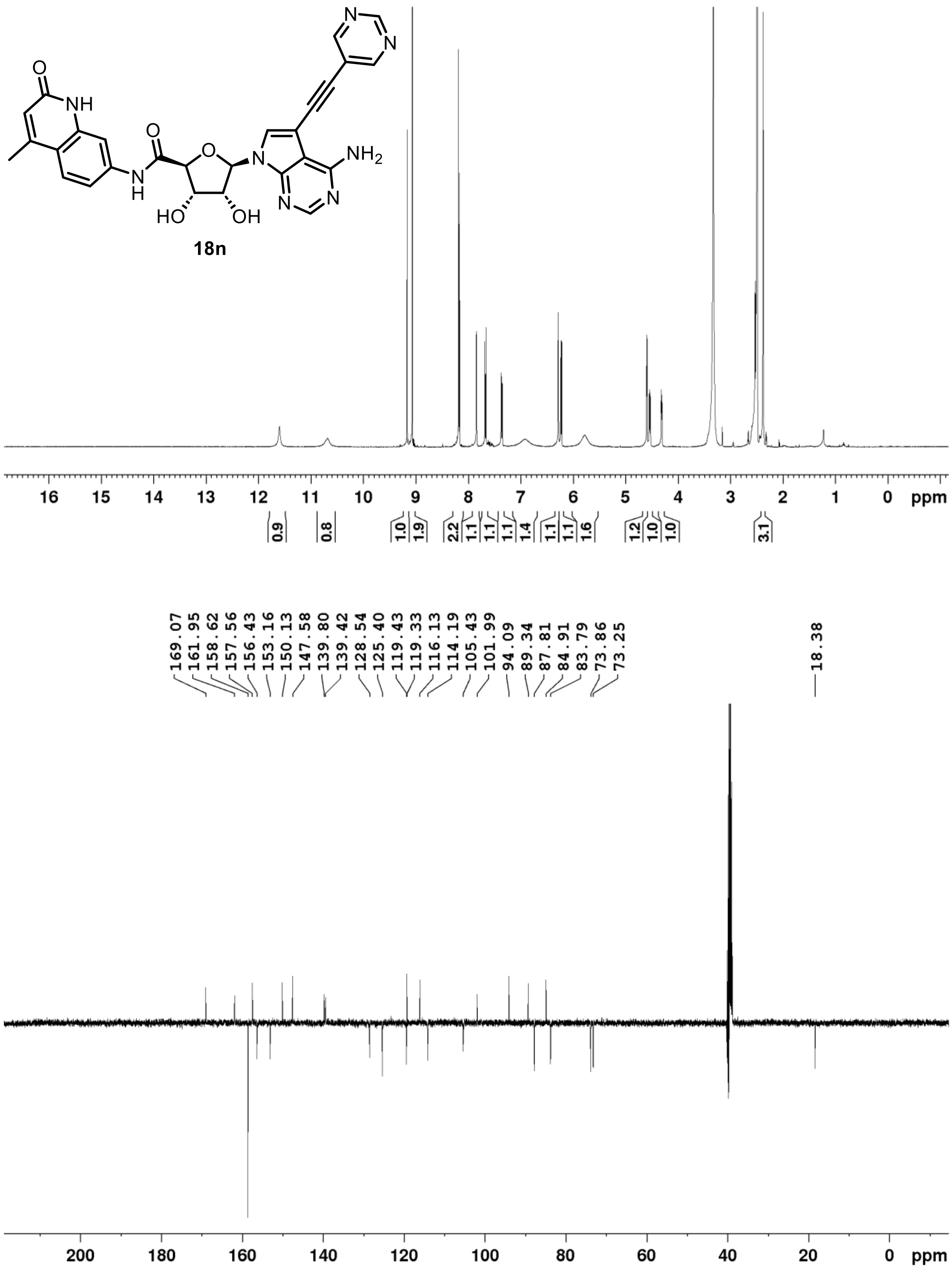

Figure S24. ^{1}H (top) and ^{13}C APT (bottom) NMR spectra of **18n** measured in DMSO-d_6.

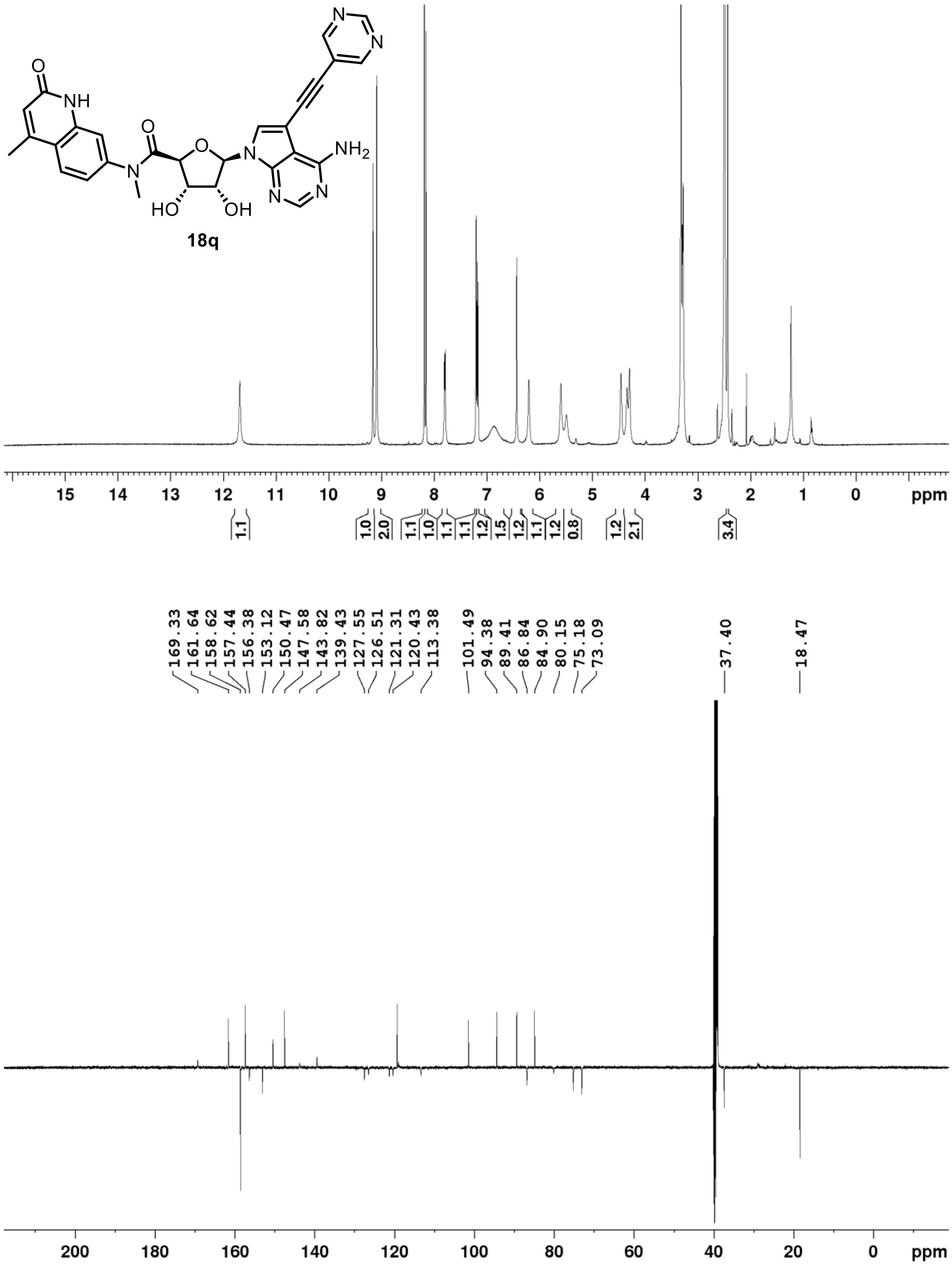

Figure S25. ^{1}H (top) and ^{13}C APT (bottom) NMR spectra of **18q** measured in DMSO-d_6.

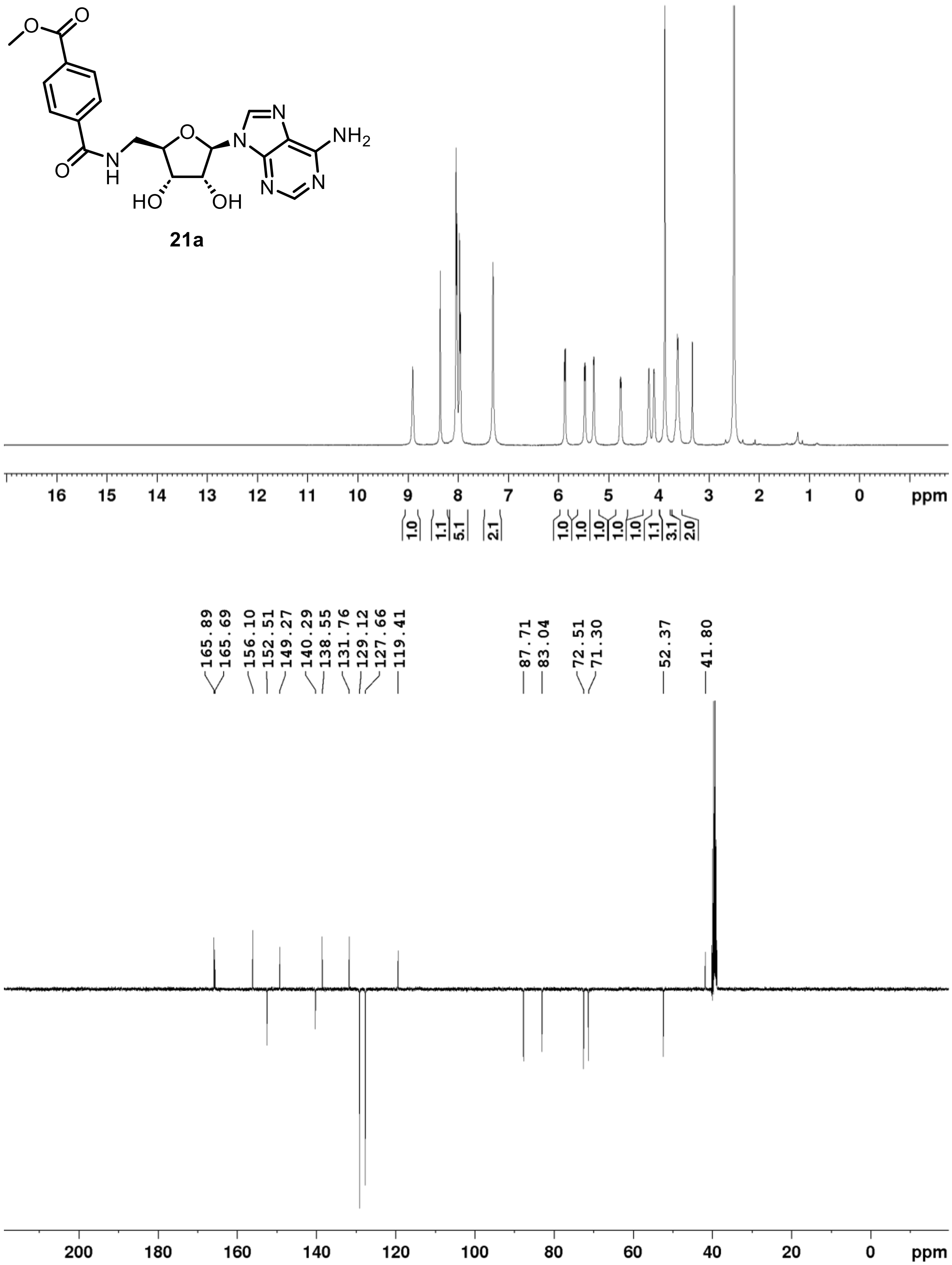

Figure S26. ^{1}H (top) and ^{13}C APT (bottom) NMR spectra of **21a** measured in DMSO-d_6.

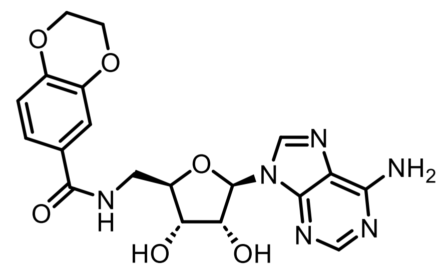

21k

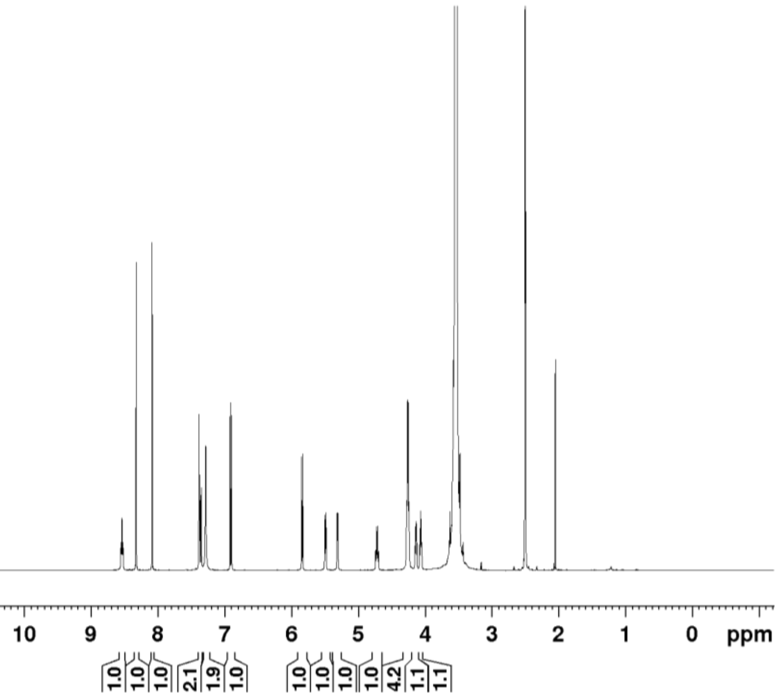

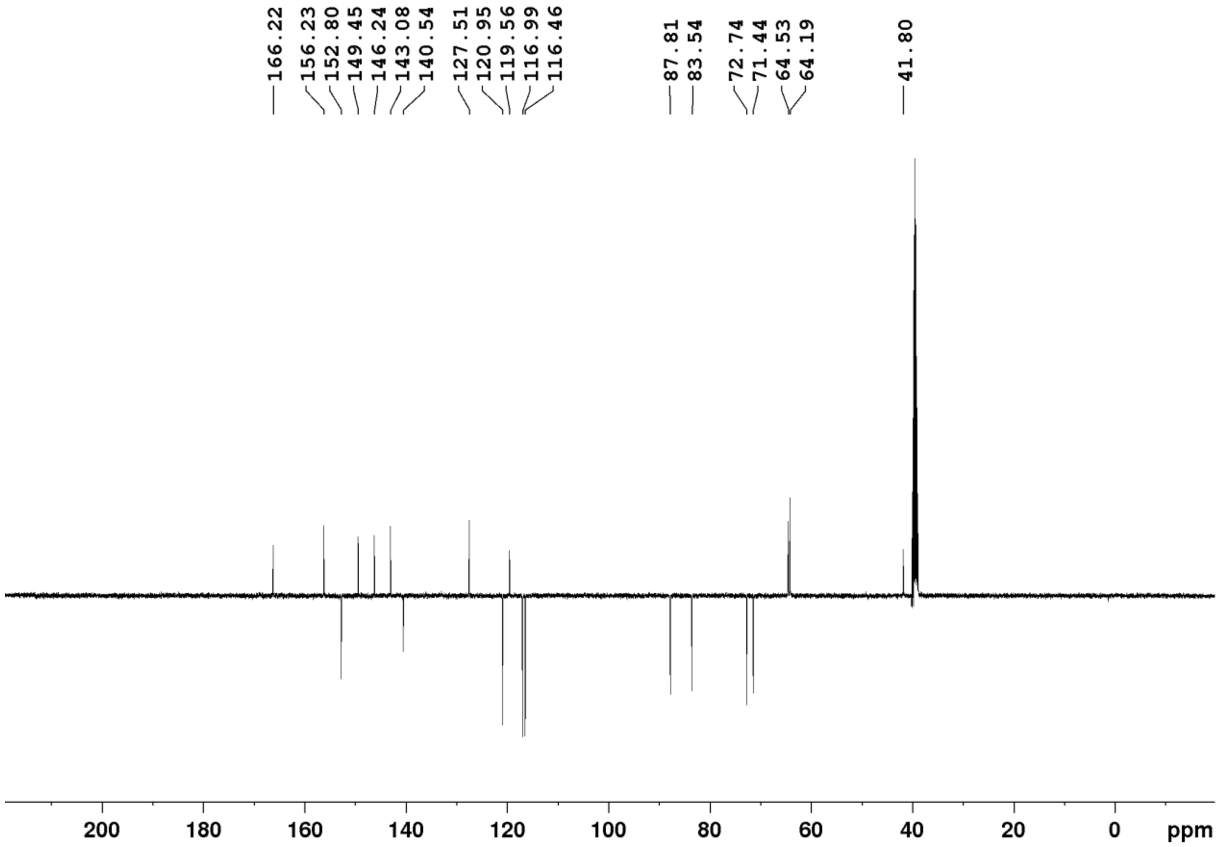

Figure S27. ^{1}H (top) and ^{13}C APT (bottom) NMR spectra of **21k** measured in DMSO-d_6.

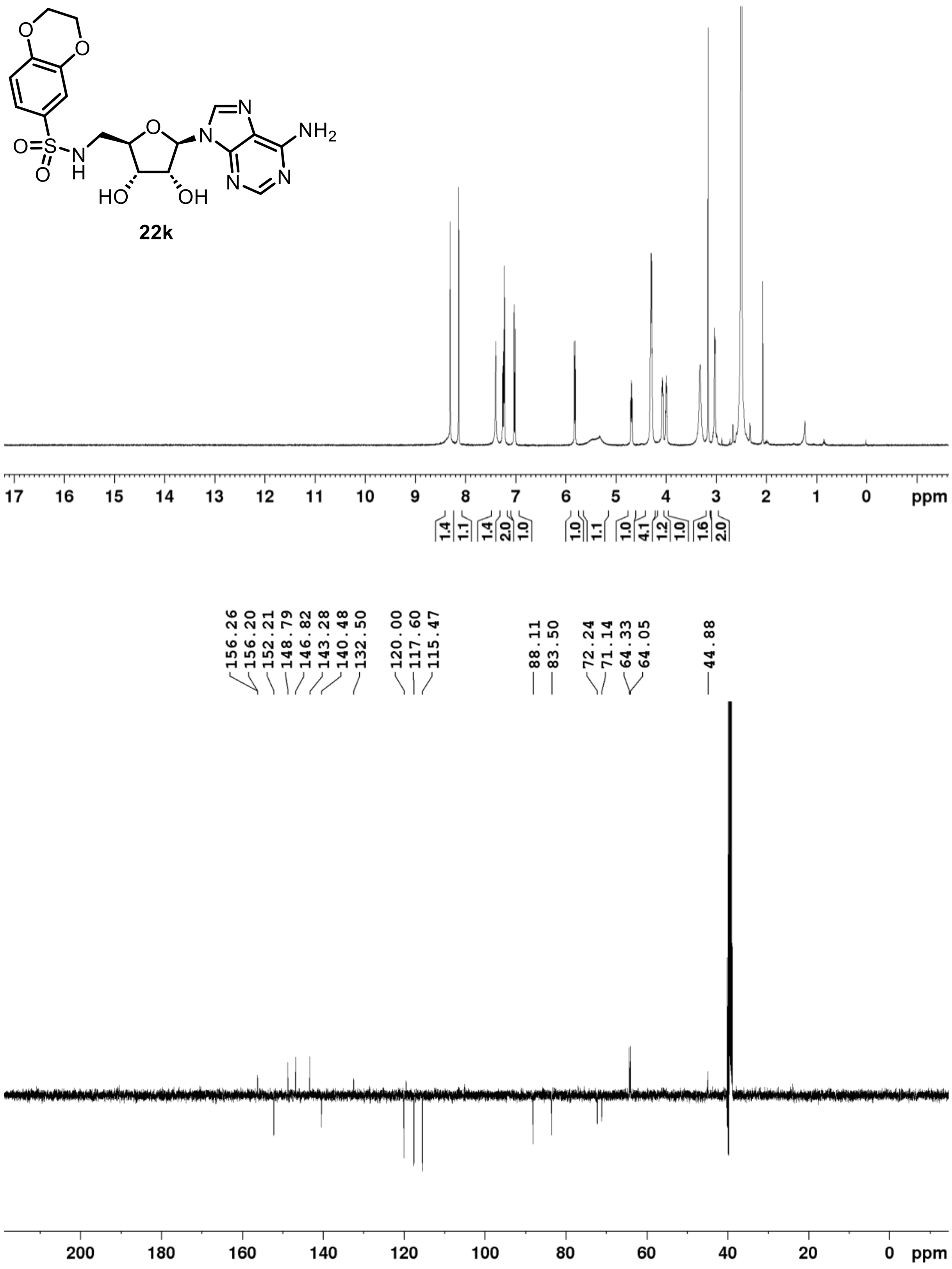

Figure S28. ^{1}H (top) and ^{13}C APT (bottom) NMR spectra of **22k** measured in DMSO-d_6.

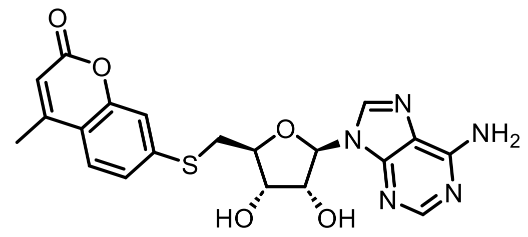

24

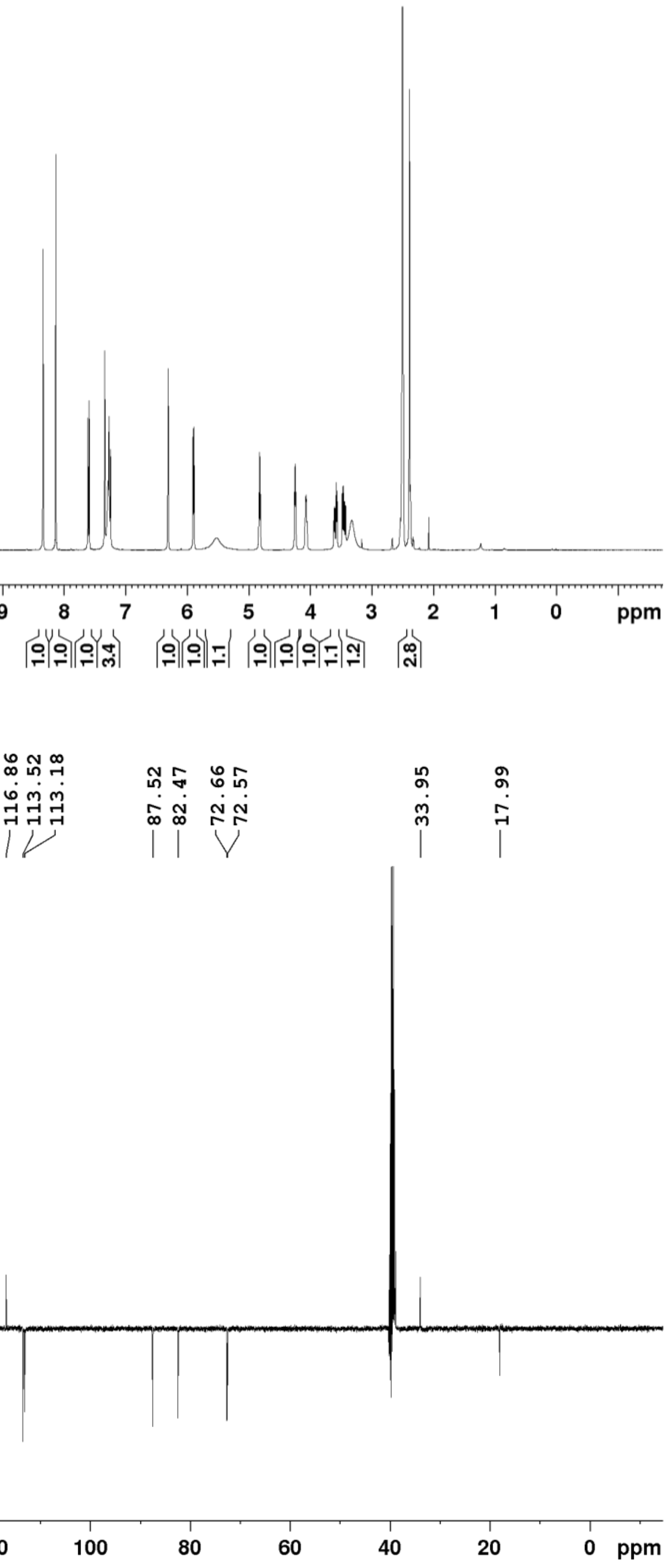

Figure S29. ^{1}H (top) and ^{13}C APT (bottom) NMR spectra of **24** measured in DMSO-d_6.

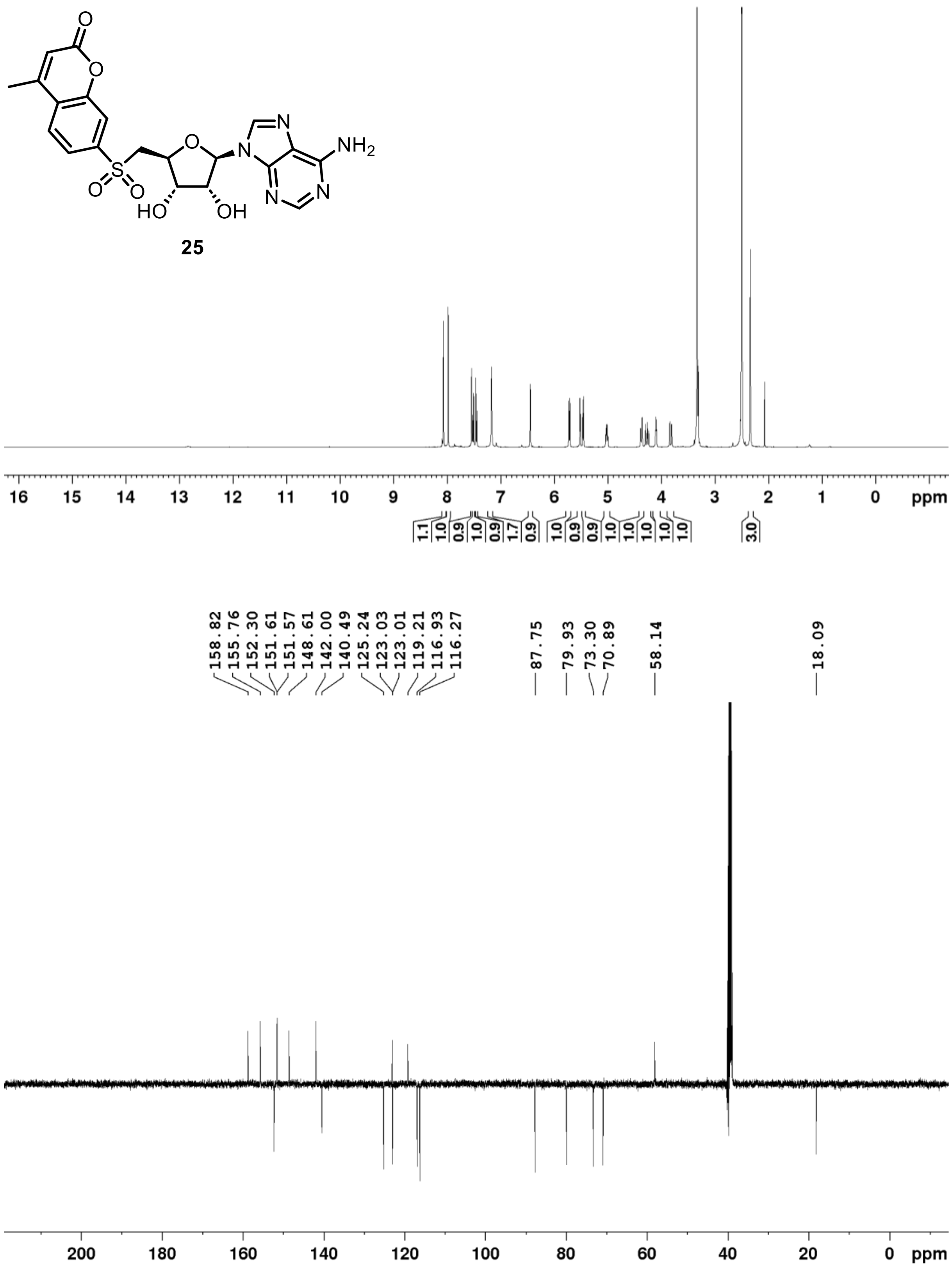

Figure S30. ^{1}H (top) and ^{13}C APT (bottom) NMR spectra of **25** measured in DMSO-d_6.

5. UPLC purity of final compounds

The UPLC purity of the final compounds **12a–p**, **18l**, **18n**, **18q**, **21a**, **21k**, **22k**, **24**, and **25** was measured as the percentage of area under the curve in chromatograms at 254 to 256 nm. The samples were prepared as solutions in methanol. Water served as mobile phase A, and acetonitrile as mobile phase B. A linear gradient from 0 to 100 % of mobile phase B in mobile phase A over 3.5 minutes was used for the analysis.

Compound	Purity (%)
**12a**	99.90
**12b**	99.80
**12c**	99.94
**12d**	99.93
**12e**	99.94
**12f**	99.94
**12g**	95.72
**12h**	99.72
**12i**	98.35
**12j**	99.81
**12k**	99.98
**12l**	99.64
**12m**	99.47
**12n**	99.02
**12o**	99.89
**12p**	100.0
**18l**	98.20
**18n**	95.19
**18q**	99.12
**21a**	99.69
**21k**	99.75
**22k**	99.98
**24**	98.74
**25**	99.89

Table S4. Purity of final compounds determined at 254 – 256 nm using UPLC-MS.

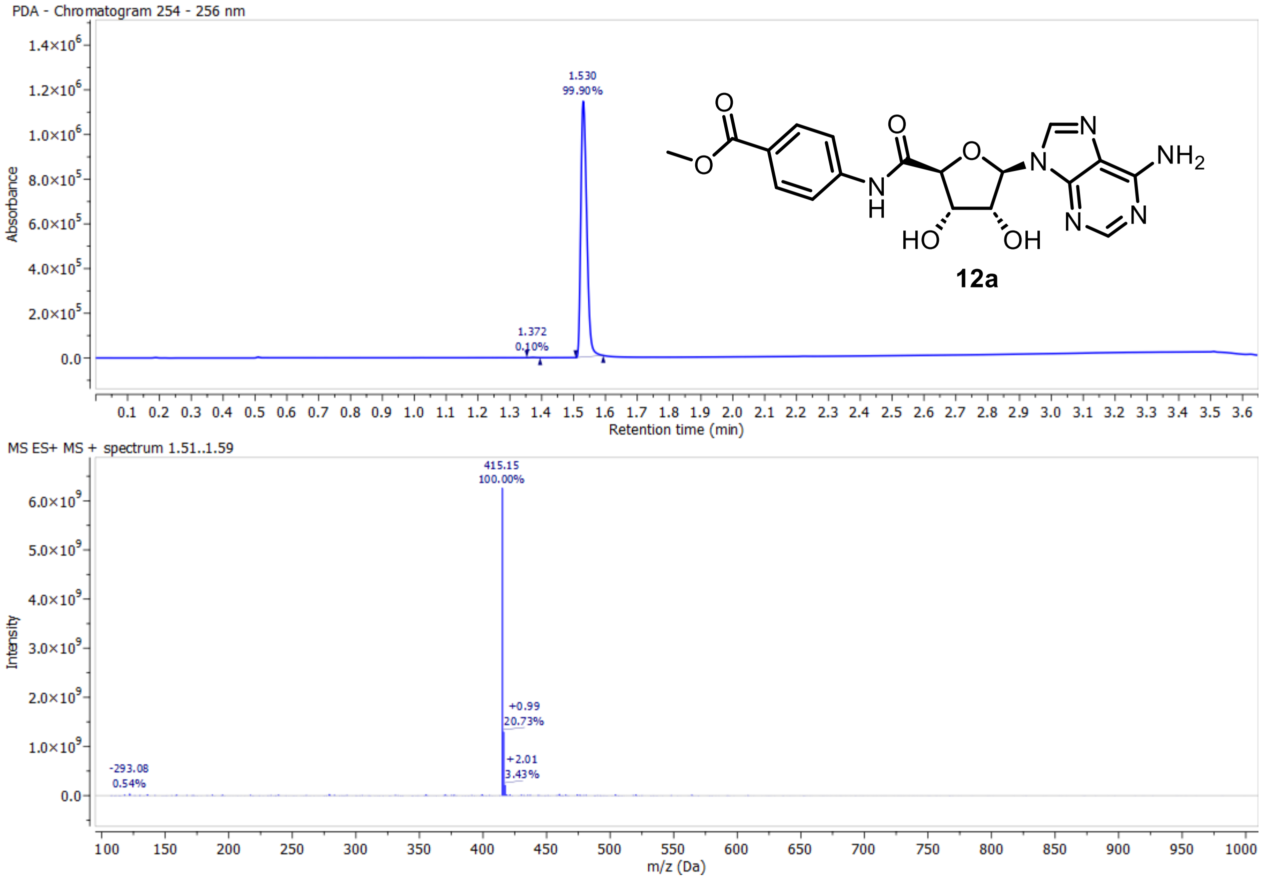

Figure S31. UPLC-MS spectra of **12a**.

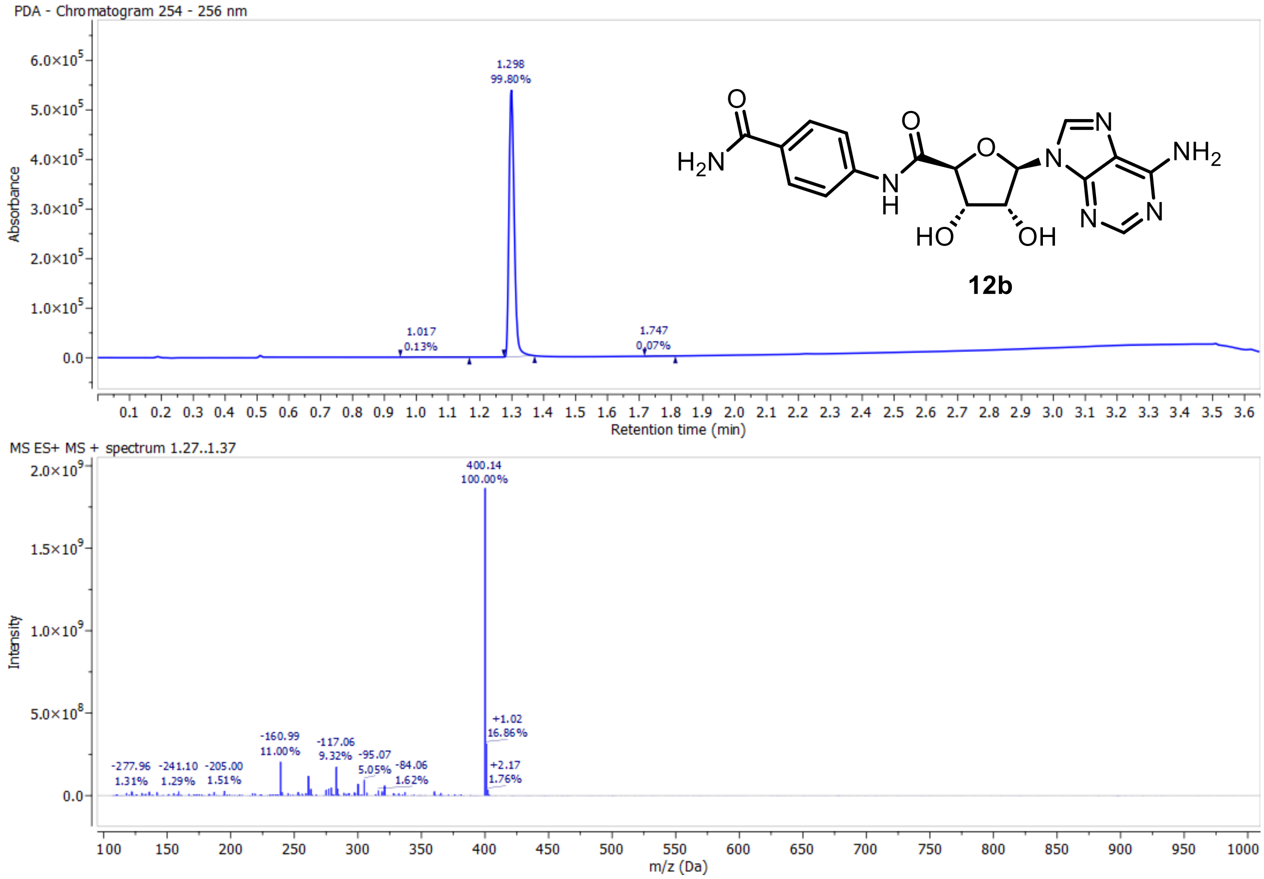

Figure S32. UPLC-MS spectra of **12b**.

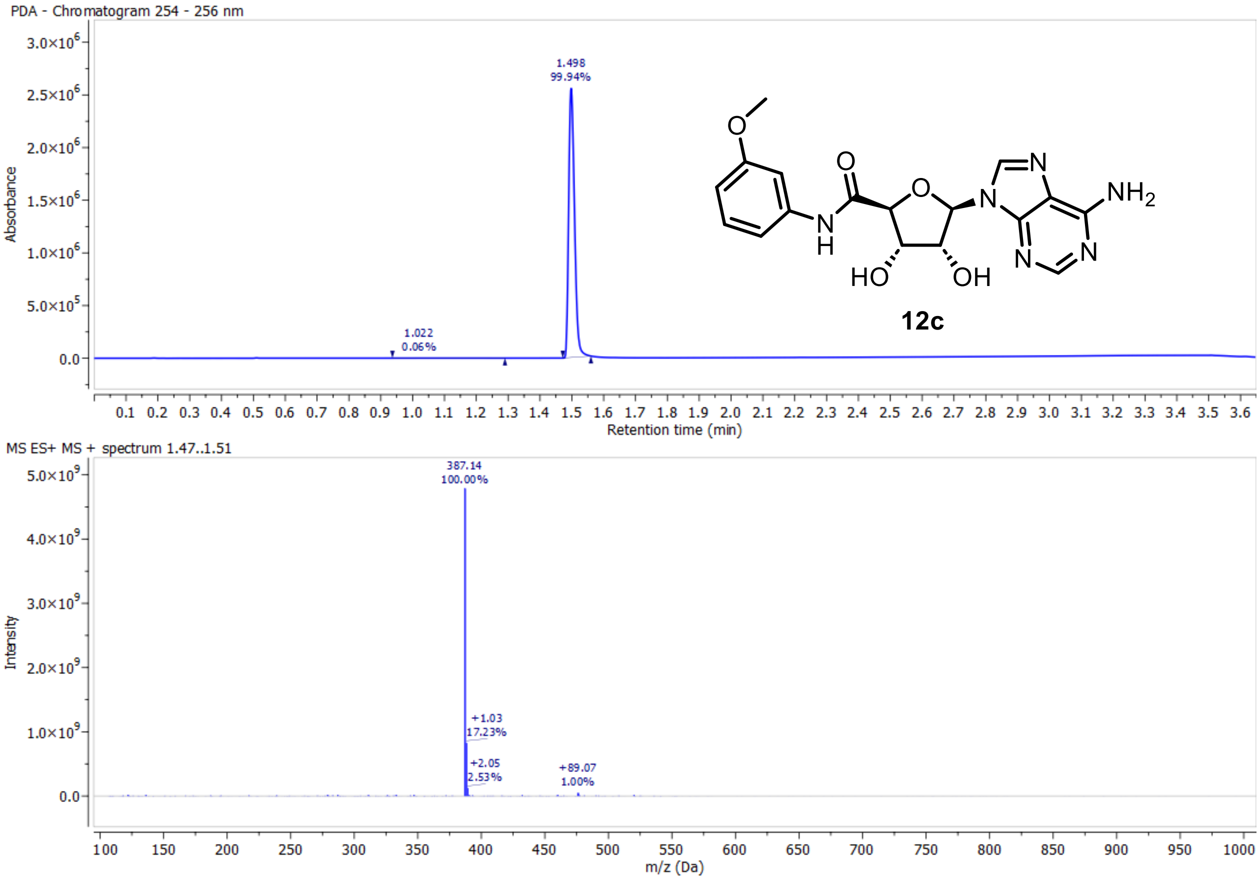

Figure S33. UPLC-MS spectra of **12c**.

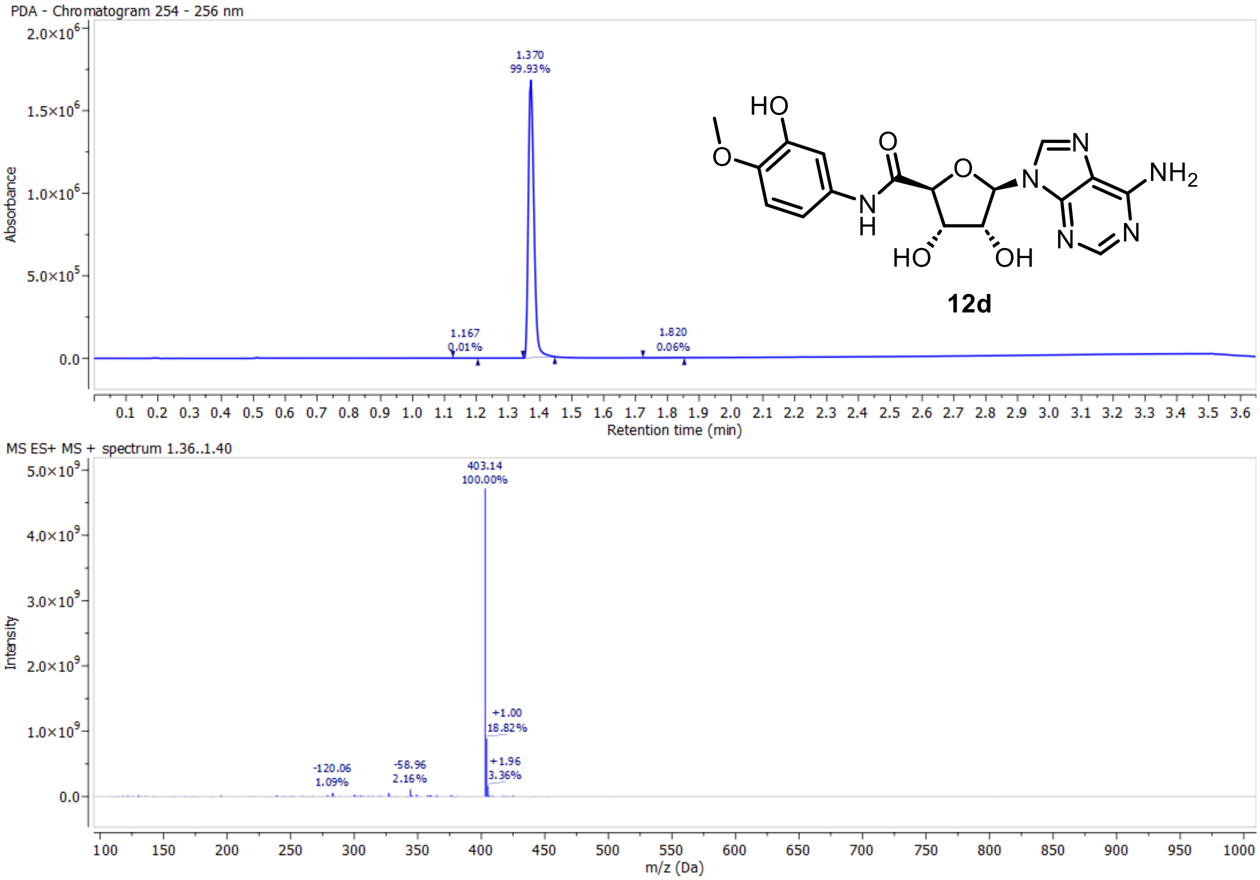

Figure S34. UPLC-MS spectra of **12d**.

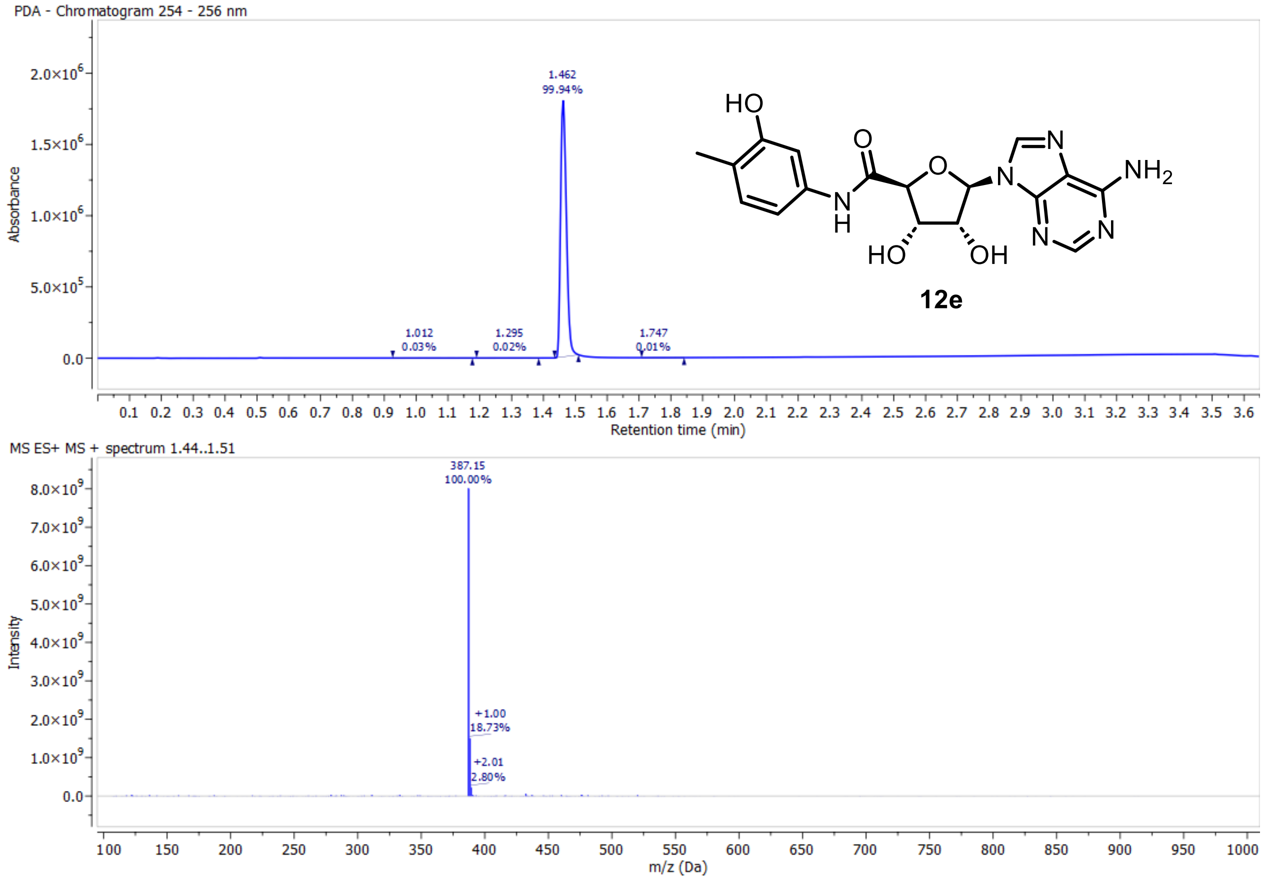

Figure S35. UPLC-MS spectra of **12e**.

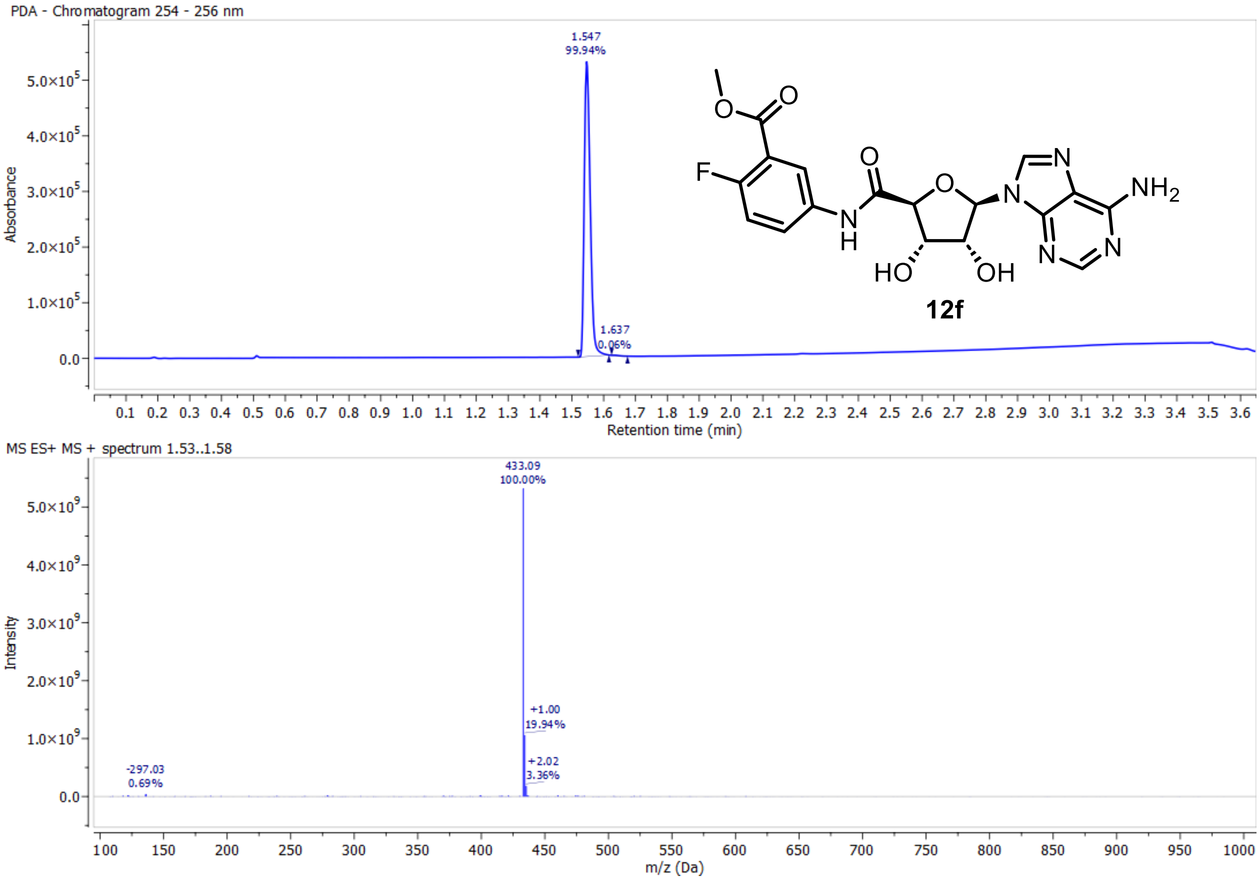

Figure S36. UPLC-MS spectra of **12f**.

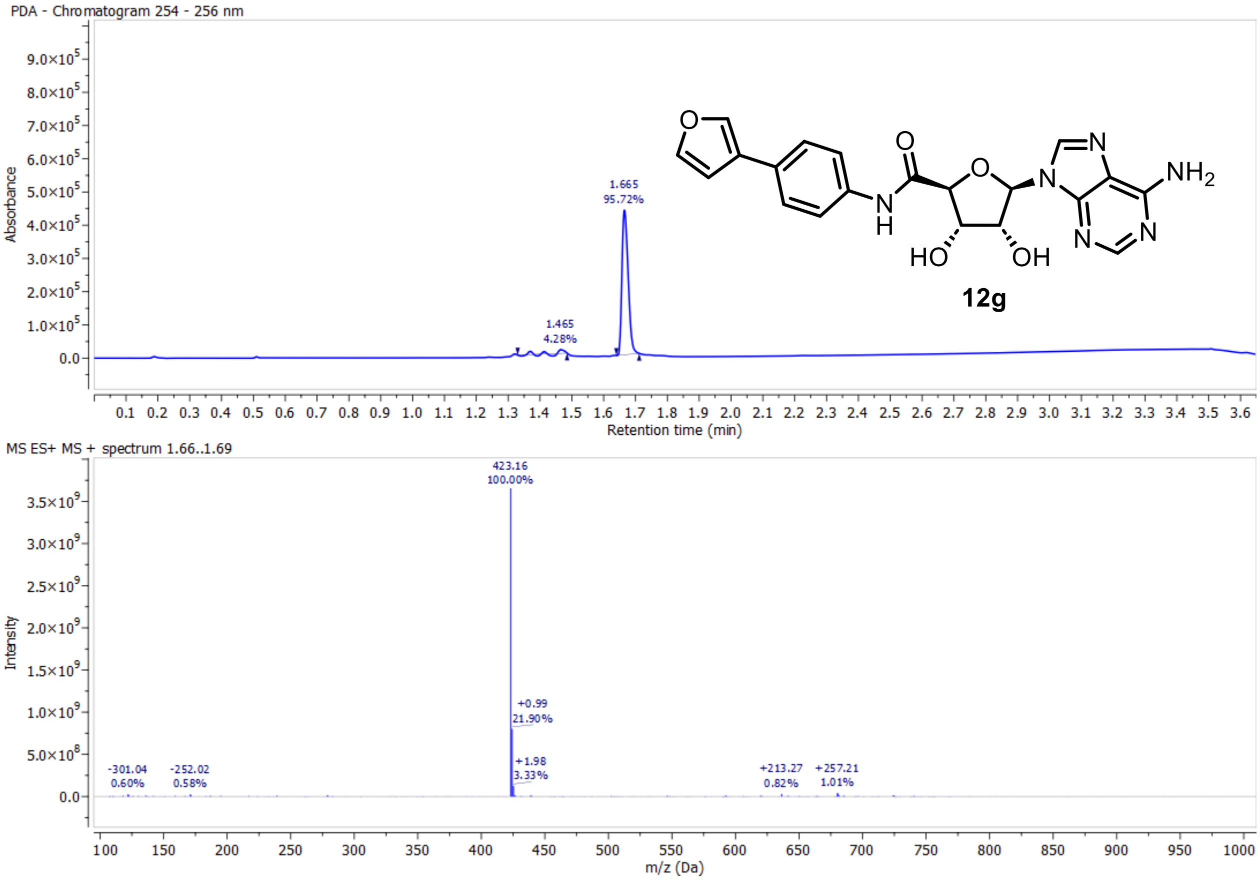

Figure S37. UPLC-MS spectra of **12g**.

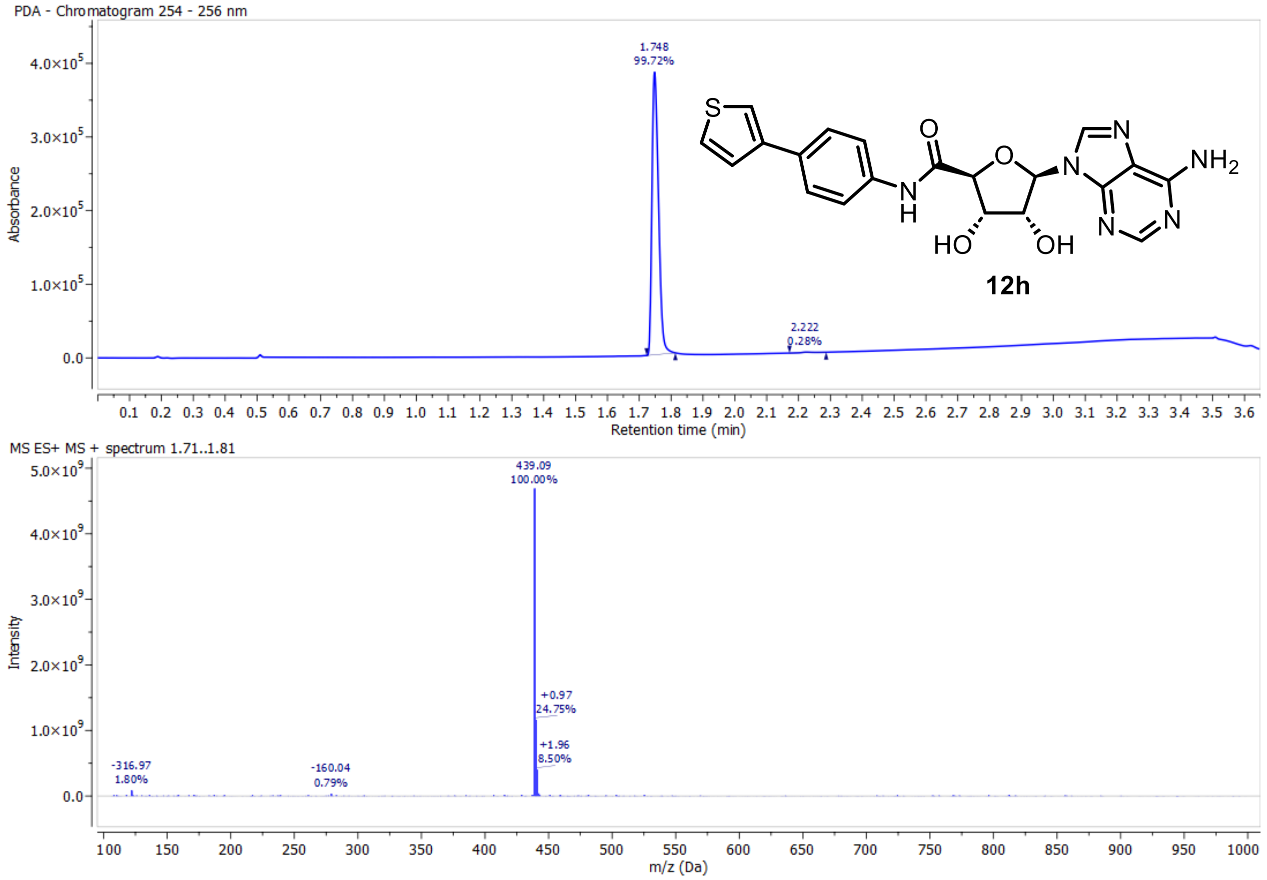

Figure S38. UPLC-MS spectra of **12h**.

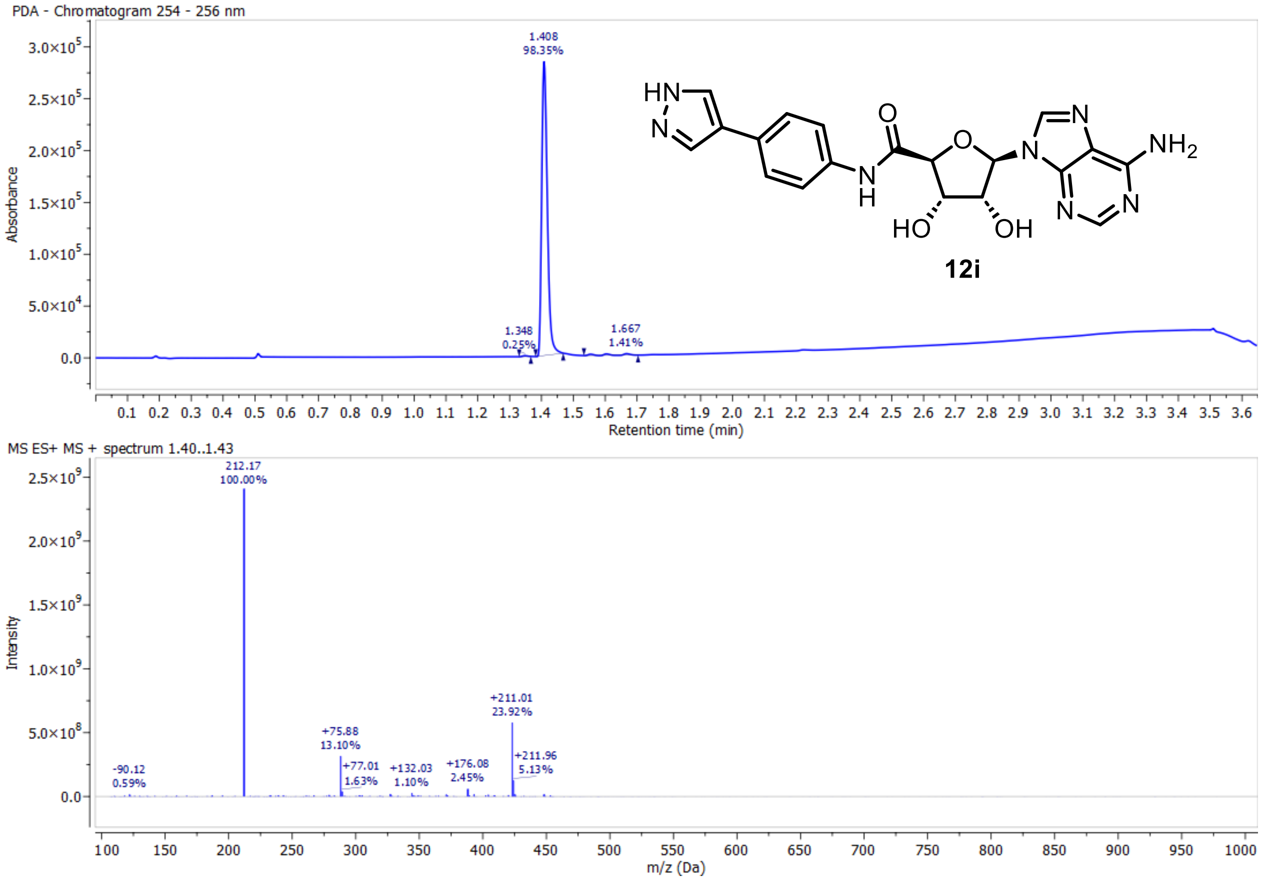

Figure S39. UPLC-MS spectra of **12i**.

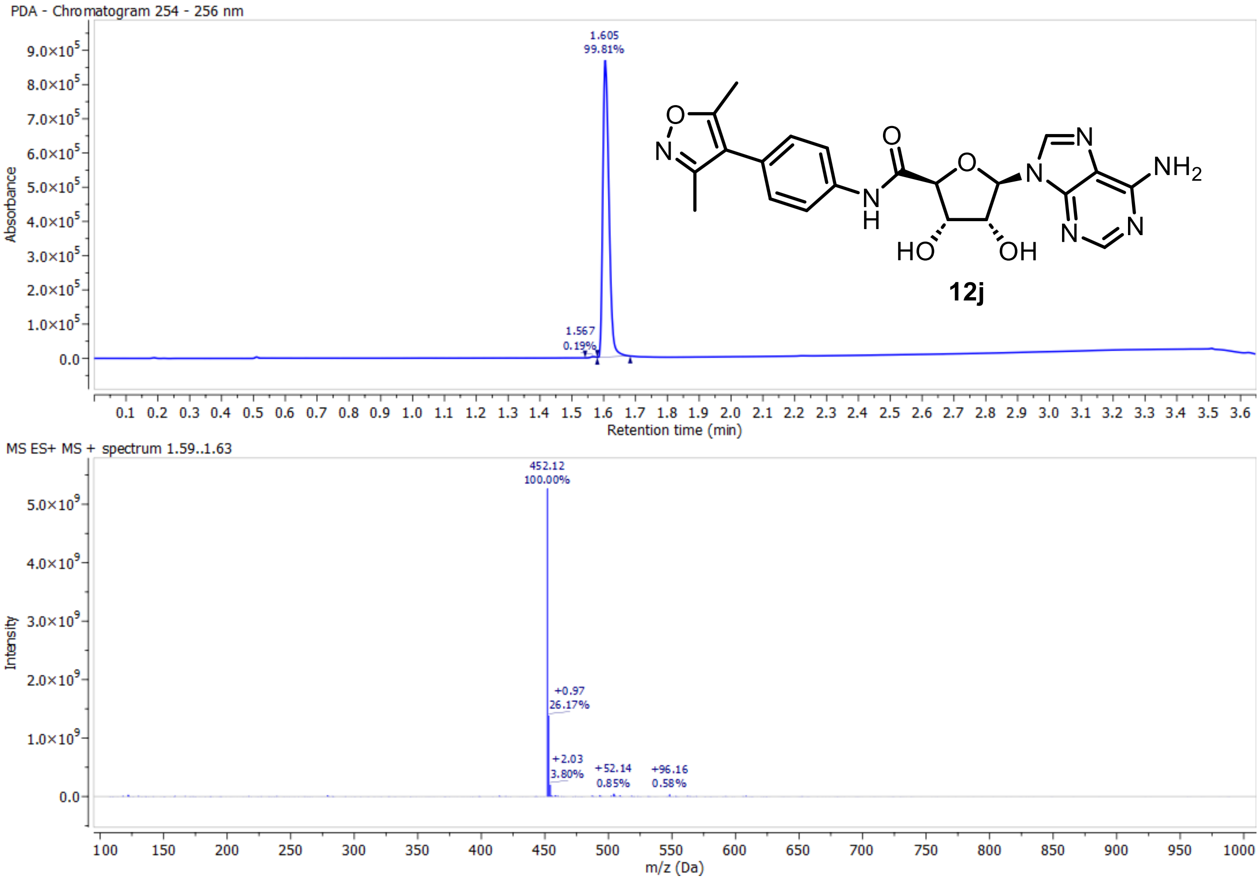

Figure S40. UPLC-MS spectra of **12j**.

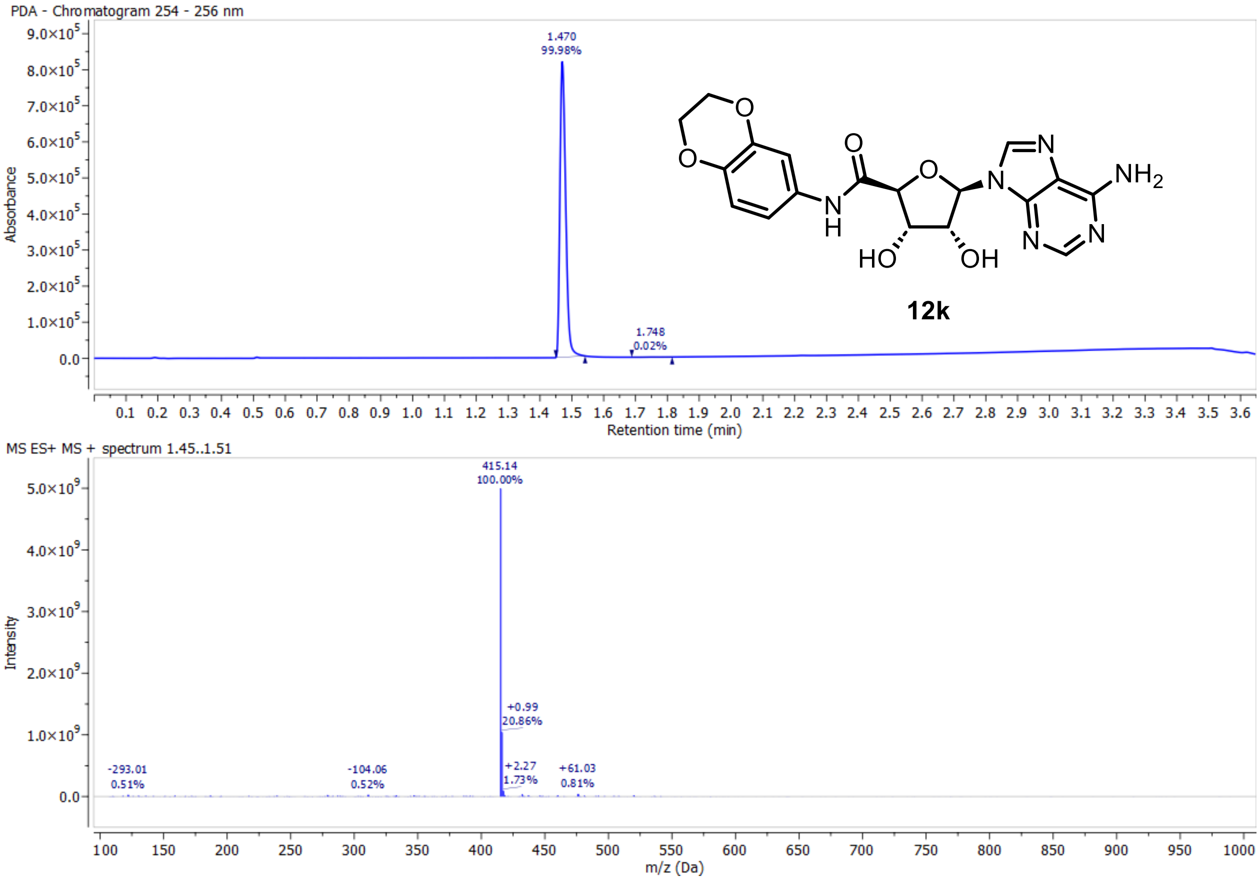

Figure S41. UPLC-MS spectra of **12k**.

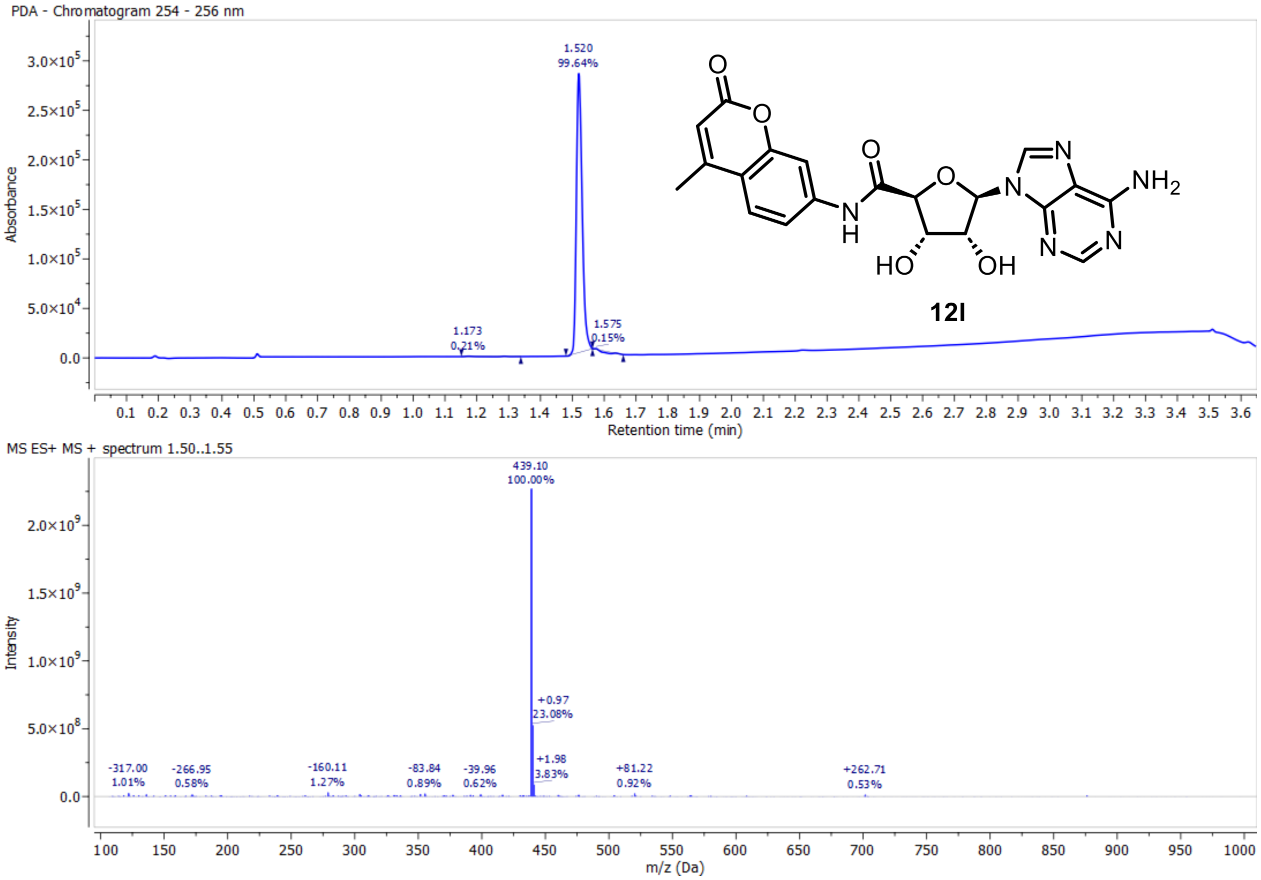

Figure S42. UPLC-MS spectra of **12l**.

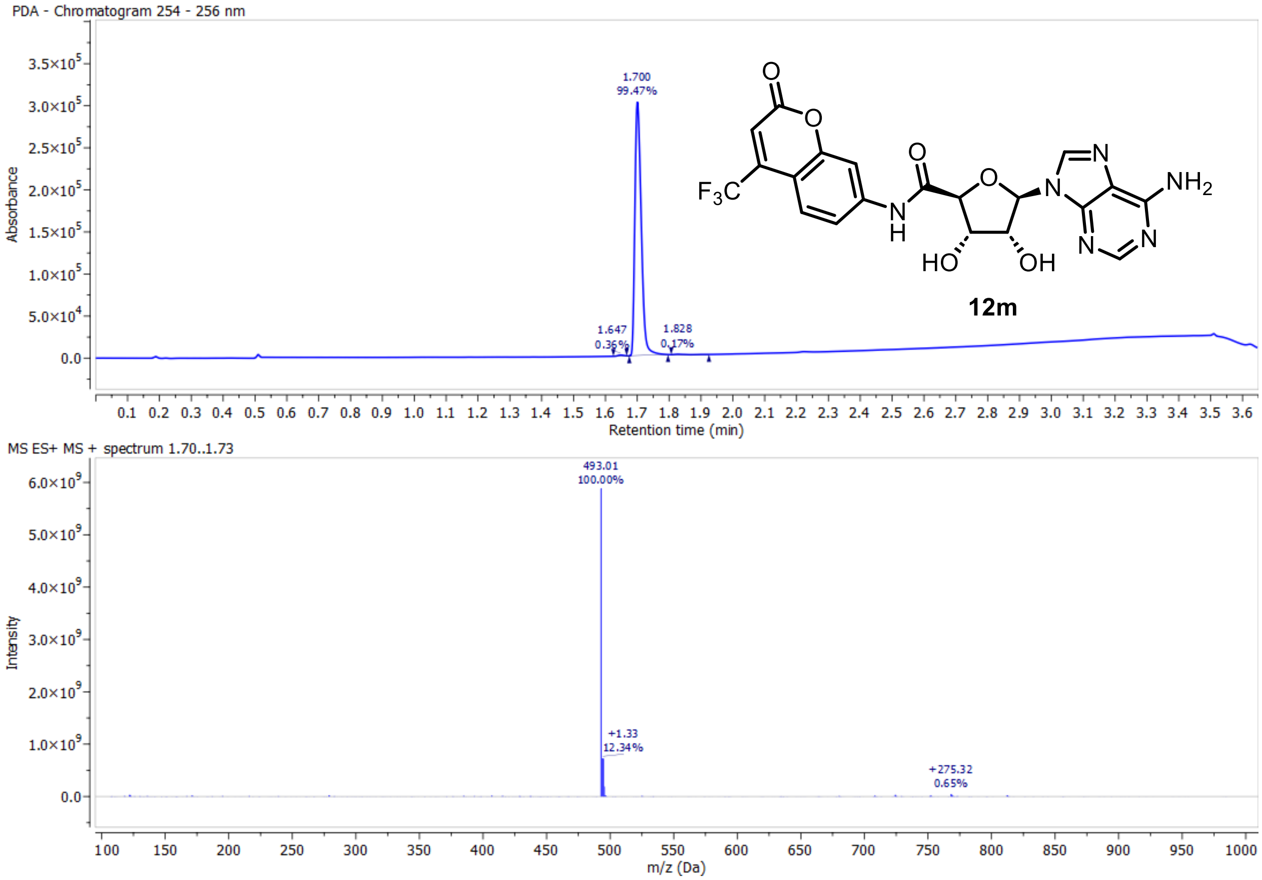

Figure S43. UPLC-MS spectra of **12m**.

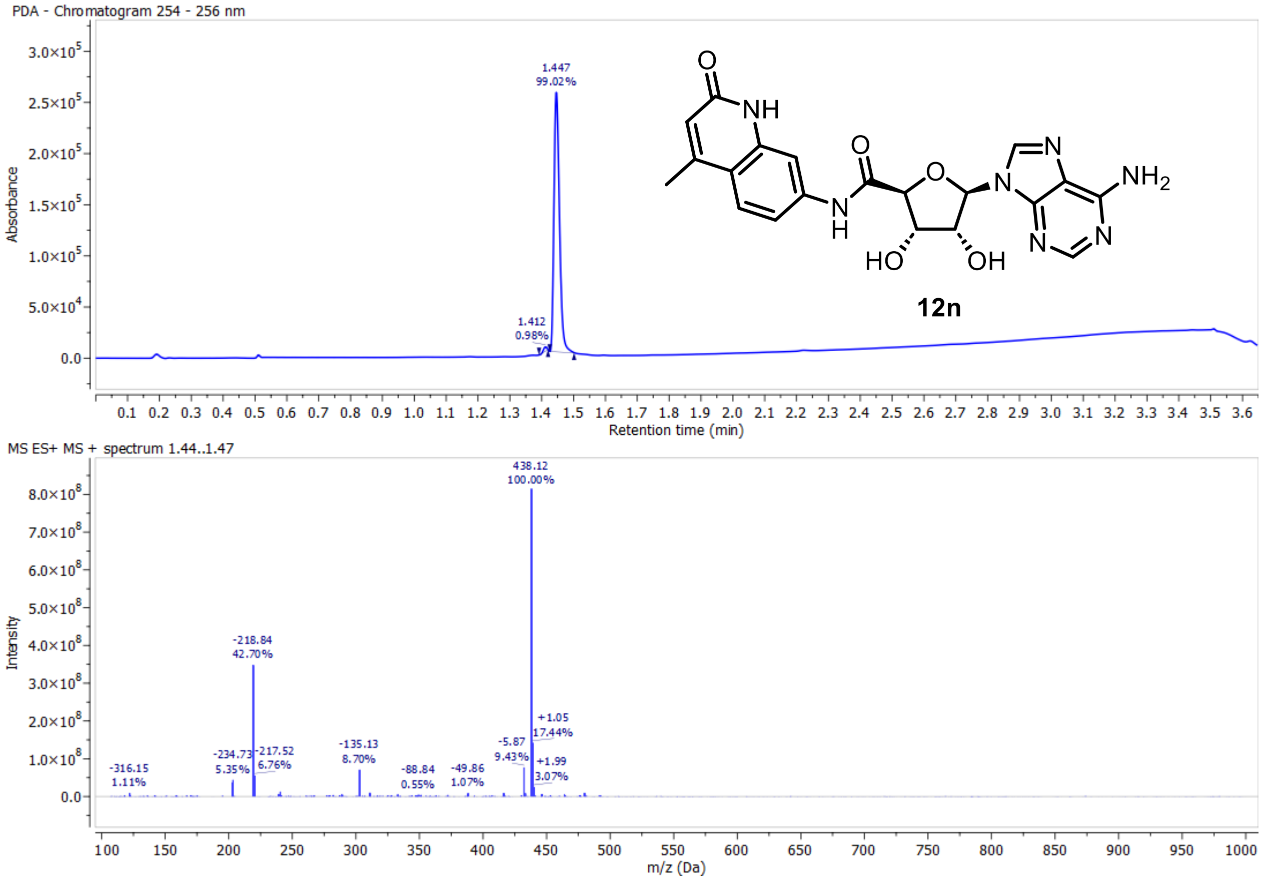

Figure S44. UPLC-MS spectra of **12n**.

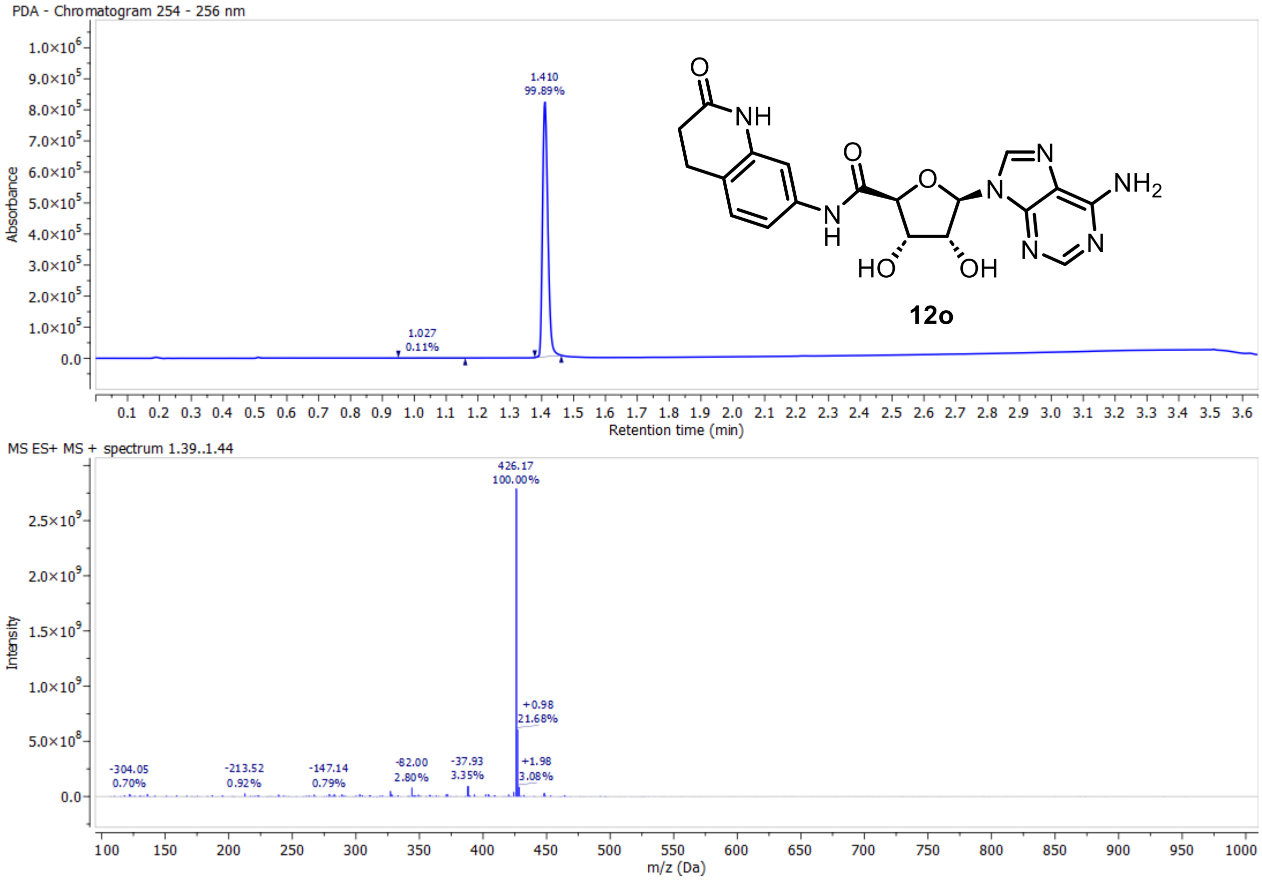

Figure S45. UPLC-MS spectra of **12o**.

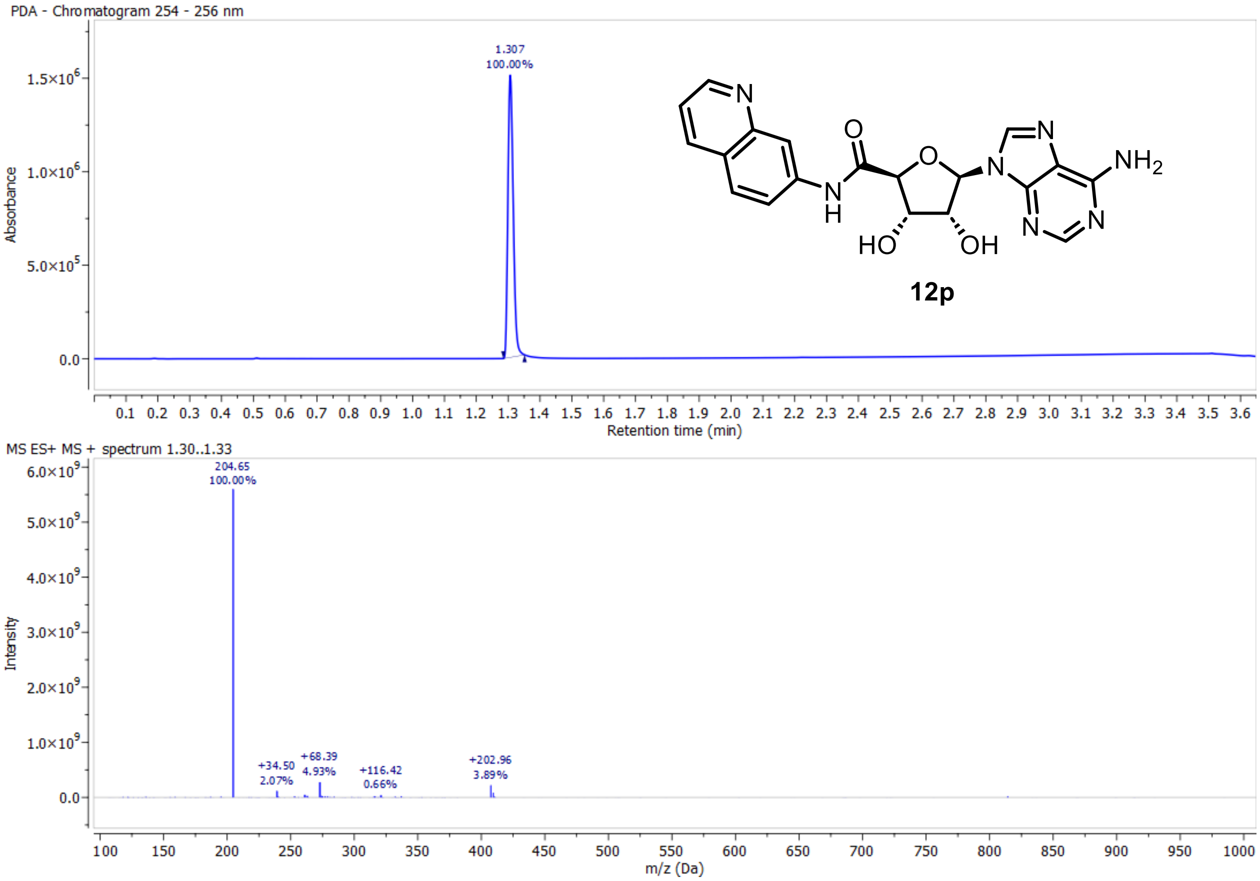

Figure S46. UPLC-MS spectra of **12p**.

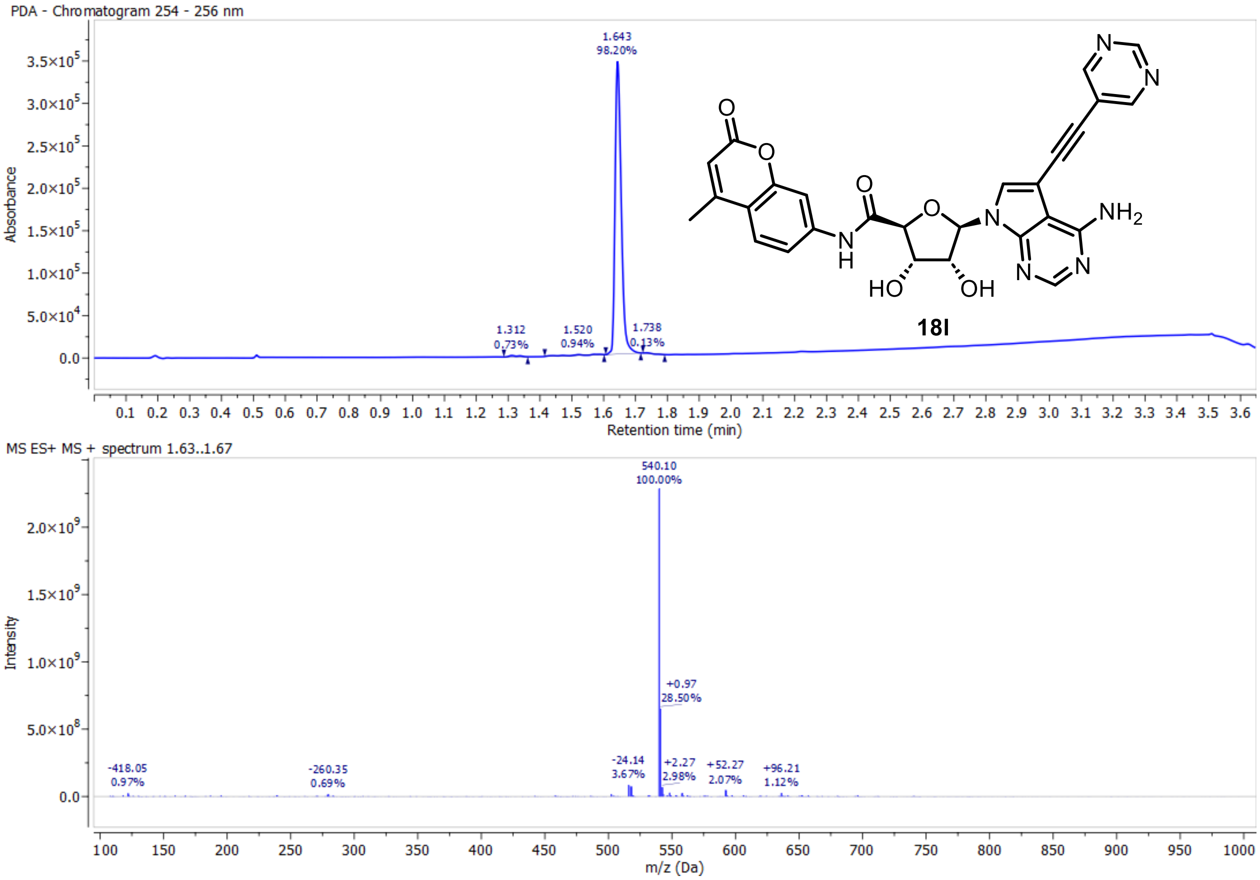

Figure S47. UPLC-MS spectra of **18l**.

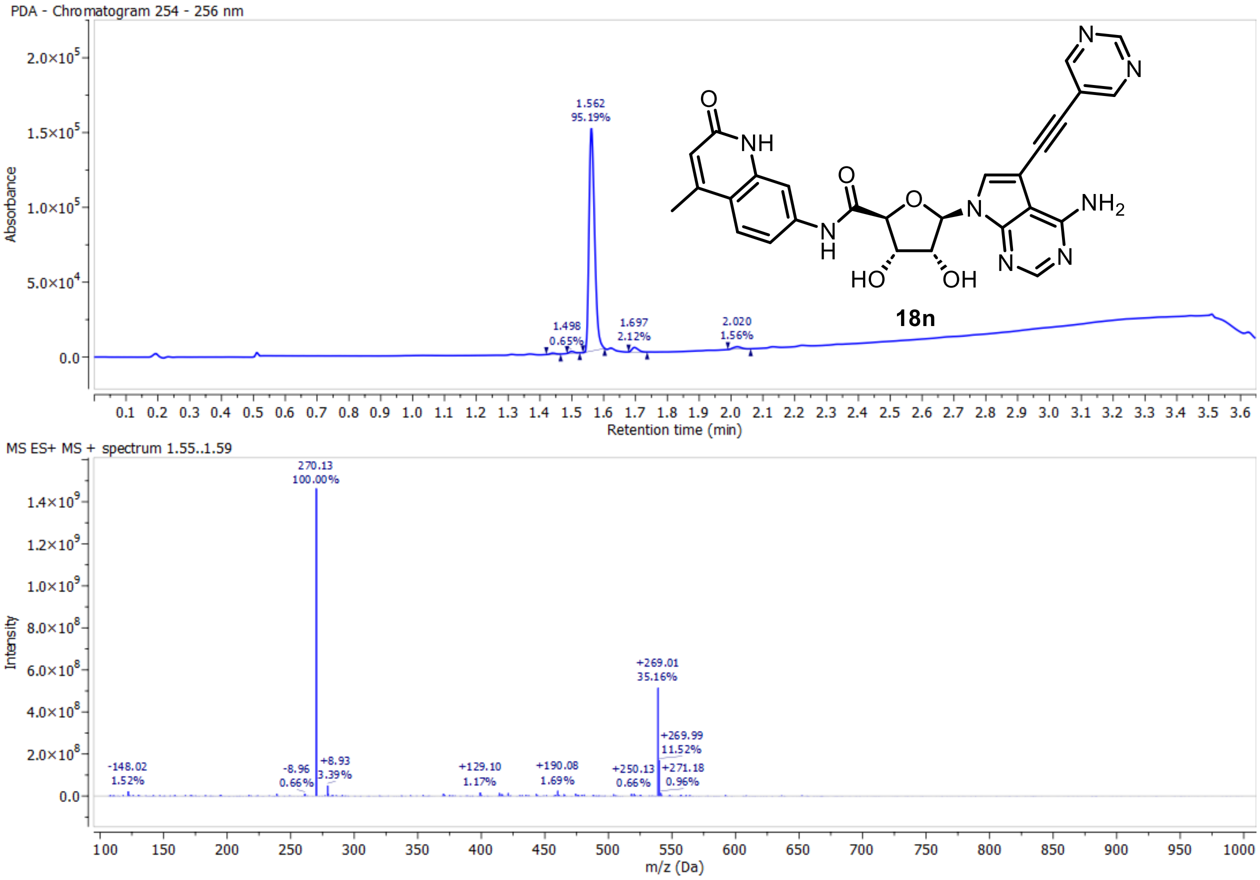

Figure S48. UPLC-MS spectra of **18n**.

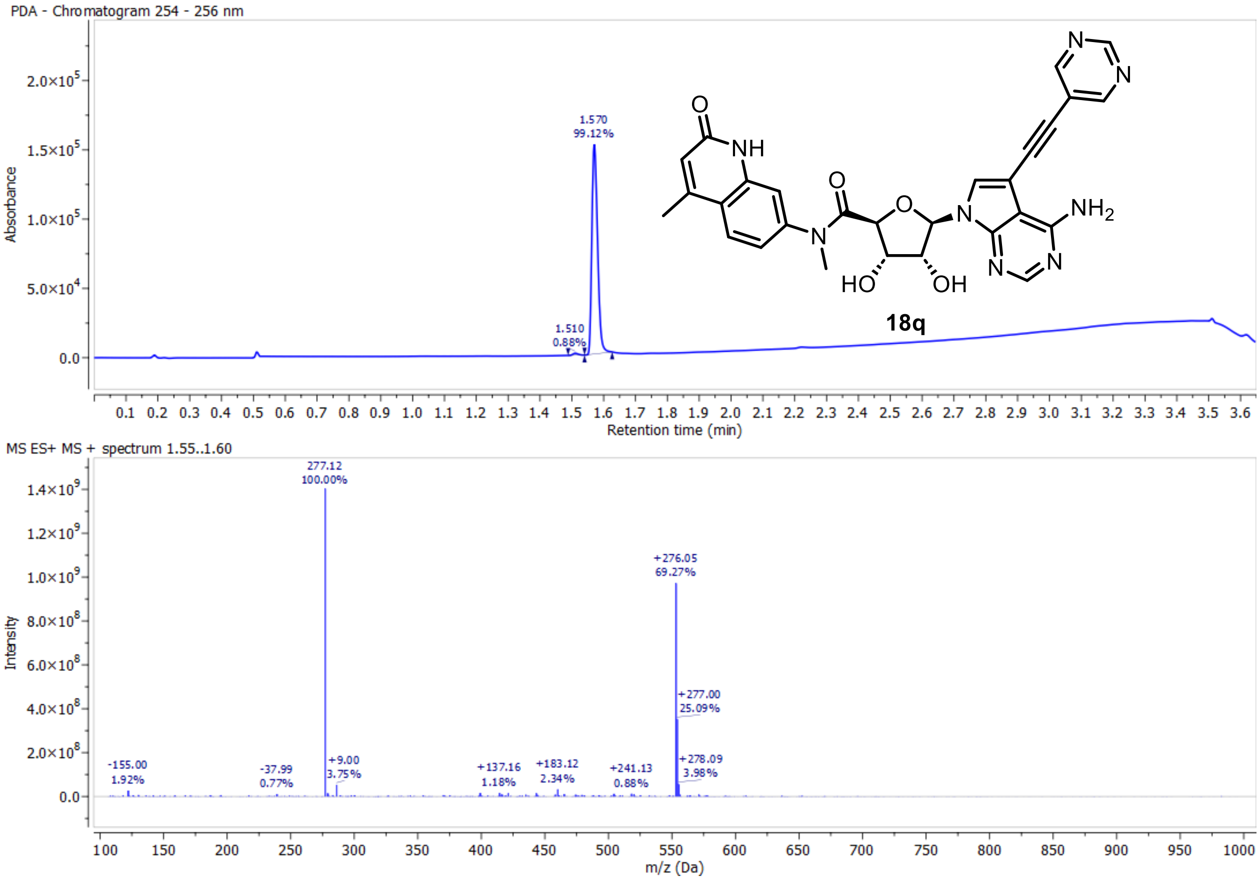

Figure S49. UPLC-MS spectra of **18q**.

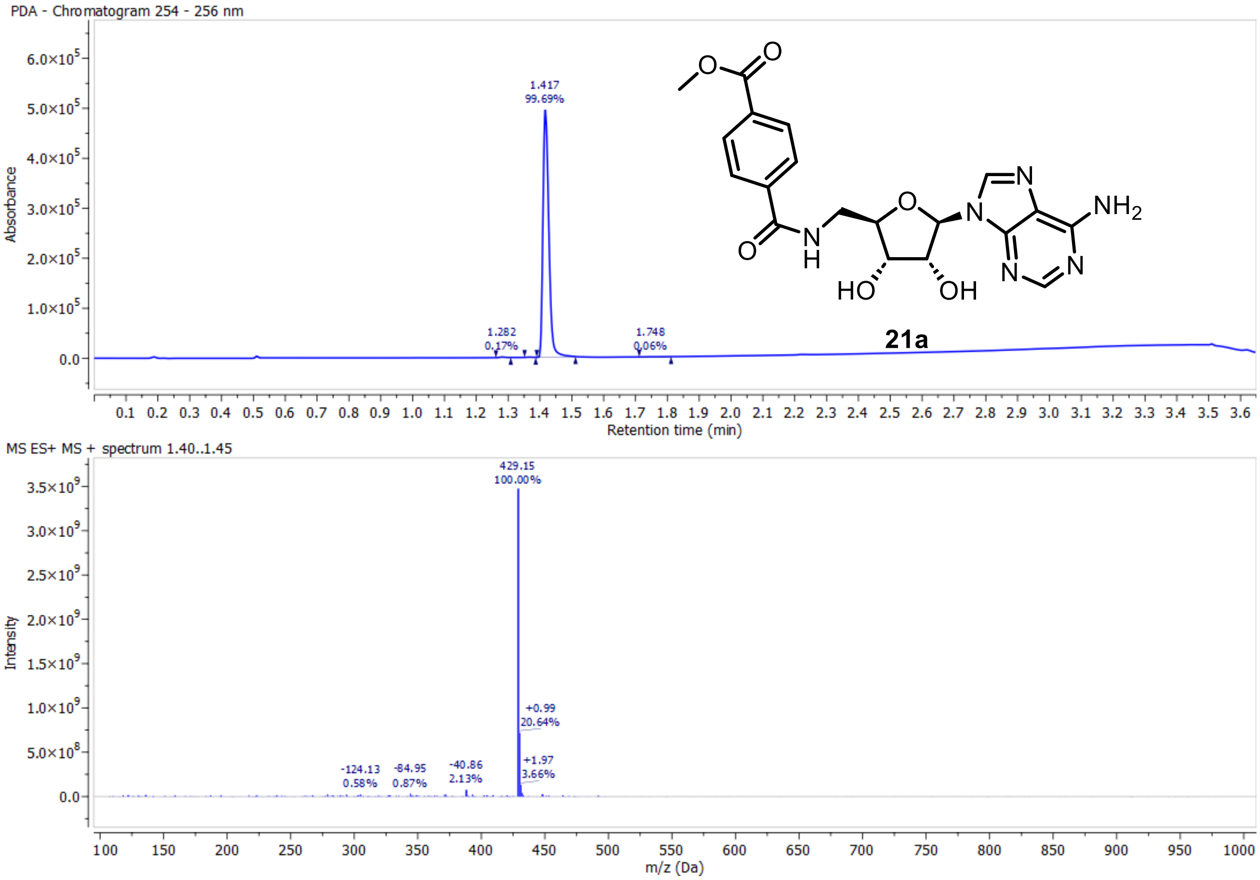

Figure S50. UPLC-MS spectra of **21a**.

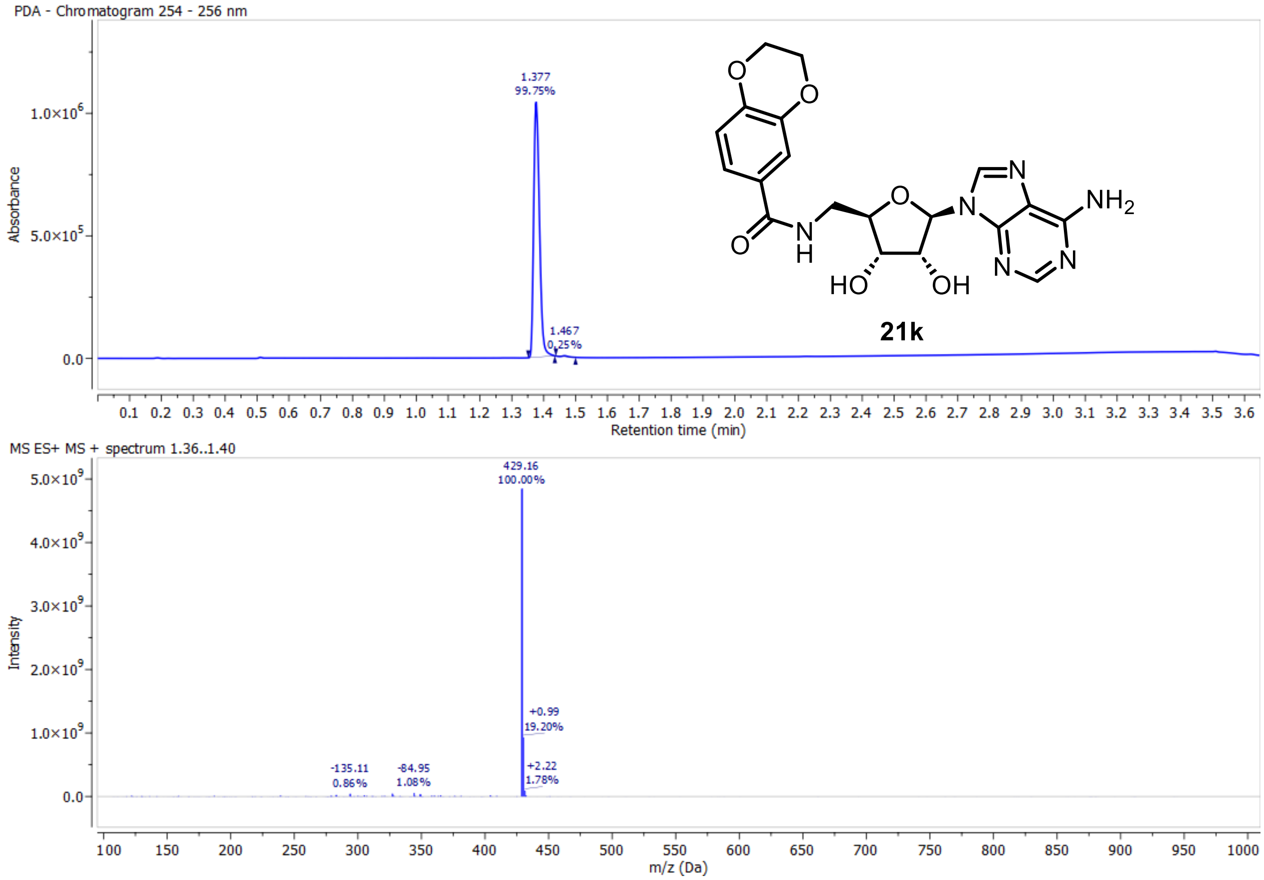

Figure S51. UPLC-MS spectra of **21k**.

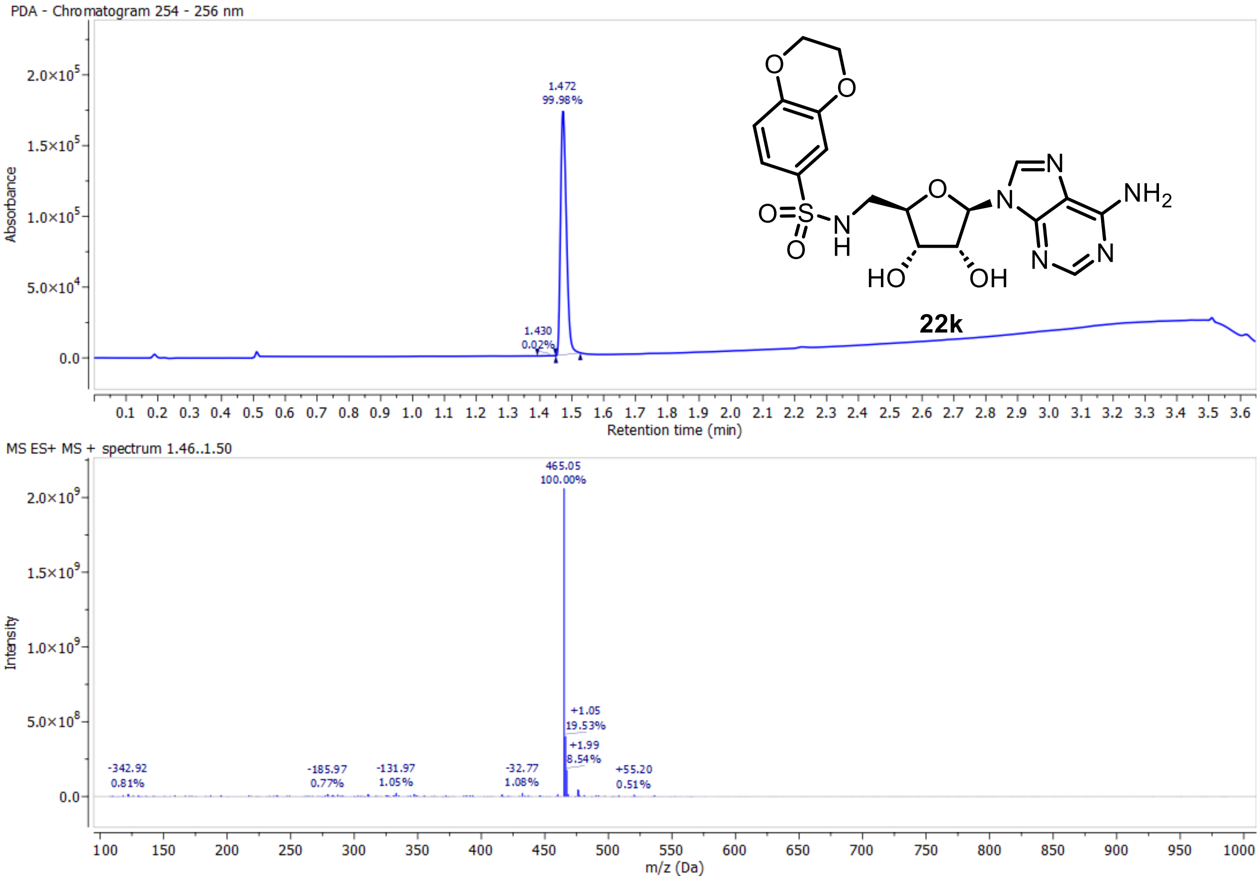

Figure S52. UPLC-MS spectra of **22k**.

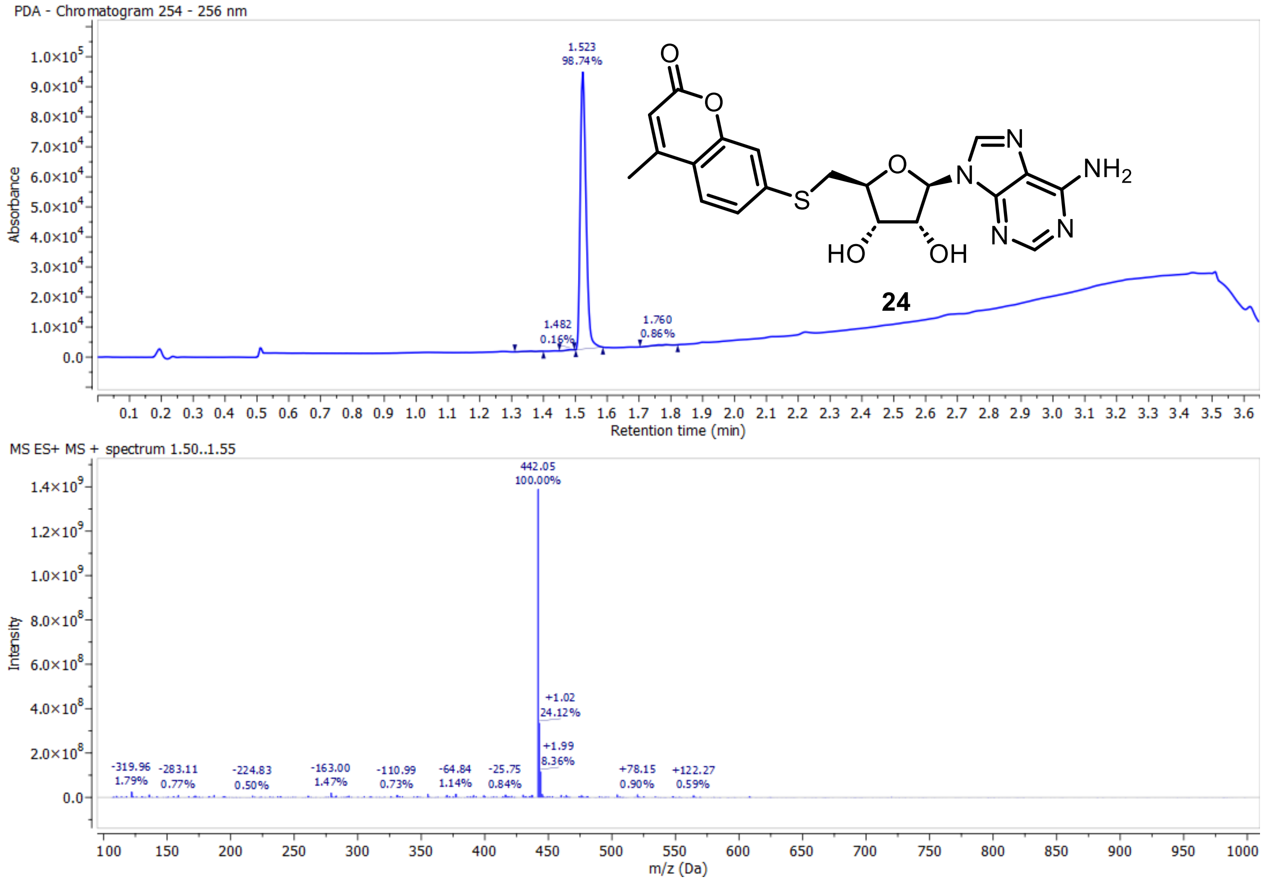

Figure S53. UPLC-MS spectra of **24**.

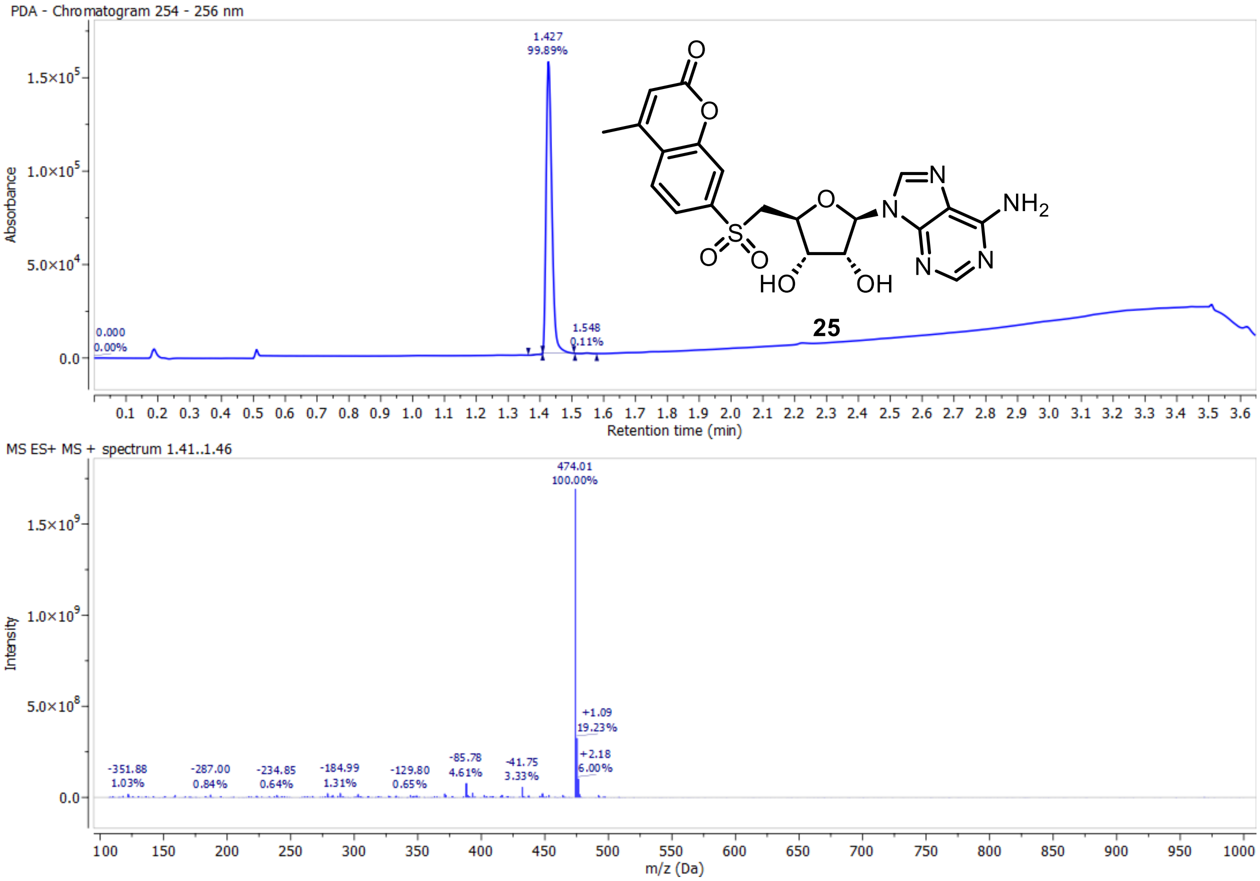

Figure S54. UPLC-MS spectra of **25**.

6. X-ray crystallography

Crystal data for 11f (colorless, 0.058 x 0.178 x 0.466 mm):

$C_{21}H_{21}FN_6O_6$, C_2H_3N, 0.1(H_2O), orthorhombic, space group $P2_12_12_1$, a = 7.9953(2) Å, b = 15.3161(4) Å, c = 19.6255(5) Å, V = 2403.28(11) Å^3, Z = 4, M = 515.29, 28864 reflections measured, 4409 independent reflections. Final R = 0.031, wR = 0.031, GoF = 0.943 for 4270 reflections with $I > 2\sigma(I)$ and 345 parameters. Flack parameter x = 0.08(3). The asymmetric unit contains one acetonitrile molecule and a partially occupied water molecule (the occupancy factor being 0,1). **CCDC 2360789**.

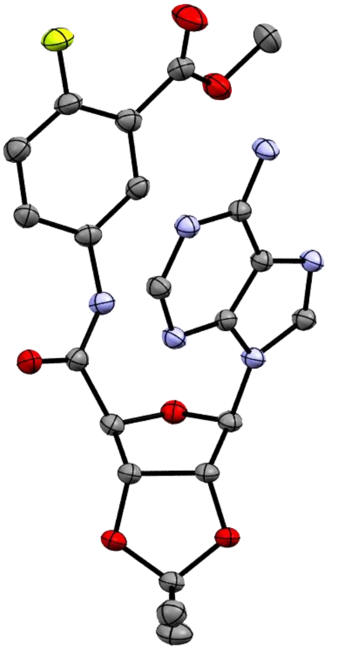

Figure S55. ORTEP diagram of **11f** drawn at 50 % probability level. Hydrogens and solvent molecules are omitted for clarity.

Crystal data for 12c (colorless, 0.100 x 0.200 x 0.710 mm):

$C_{17}H_{18}N_6O_5$, monoclinic, space group $P2_1$, a = 7.1764(2) Å, b = 13.7285(3) Å, c = 9.1307(2) Å, β = 106.2078(6)°, V = 863.81(4) Å^3, Z = 2, M = 386.37, 14894 reflections measured, 3140 independent reflections. Final R = 0.022, wR = 0.024, GoF = 1.017 for 3131 reflections with $I > 2\sigma(I)$ and 255 parameters. Flack parameter x = 0.180(18). **CCDC 2360790**.

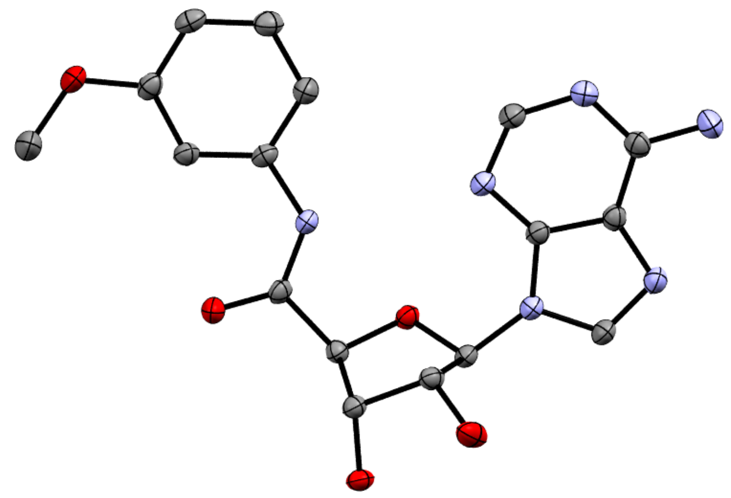

Figure S56. ORTEP diagram of **12c** drawn at 50 % probability level. Hydrogens and solvent molecules are omitted for clarity.

Crystal data for 17n (colorless, 0.062 x 0.116 x 0.294 mm):

$C_{39}H_{50}N_8O_5Si_2$, C_2H_3N, monoclinic, space group $P2_1$, a = 13.5790(4) Å, b = 11.3344(3) Å, c = 14.3892(4) Å, β = 99.3520(9) Å, V = 2185.20(11) Å^3, Z = 2, M = 808.10, 49727 reflections measured, 8550 independent reflections. Final R = 0.025, wR = 0.032, GoF = 0.996 for 8393 reflections with I > 2σ(I) and 516 parameters. Flack parameter *x* = 0.035(4). The asymmetric unit contains one acetonitrile molecule. **CCDC 2360791**.

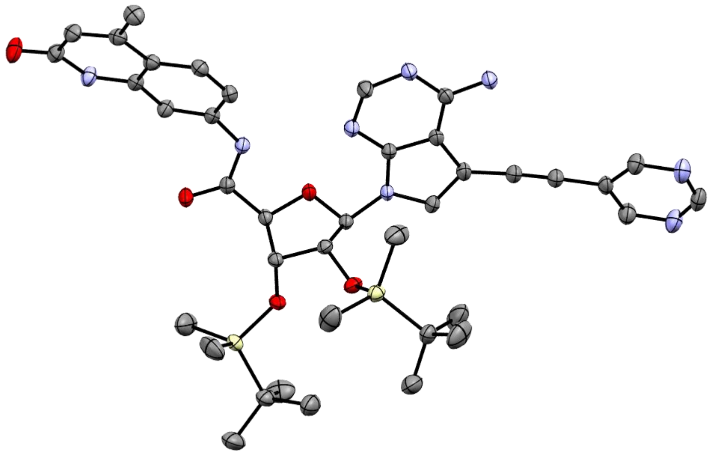

Figure S57. ORTEP diagram of **17n** drawn at 50 % probability level. Hydrogens and solvent molecules are omitted for clarity.

7. Docking study

Docking was performed using the Schrödinger Maestro software package[11] with the GlideScore scoring function. All experiments utilized the crystal structure of nsp14 (PDB ID: 7R2V, chain A) complexed with SAH. The search space was defined by the position of SAH, covering an area of approximately 15 Å^3. SAH was also used as a reference ligand.

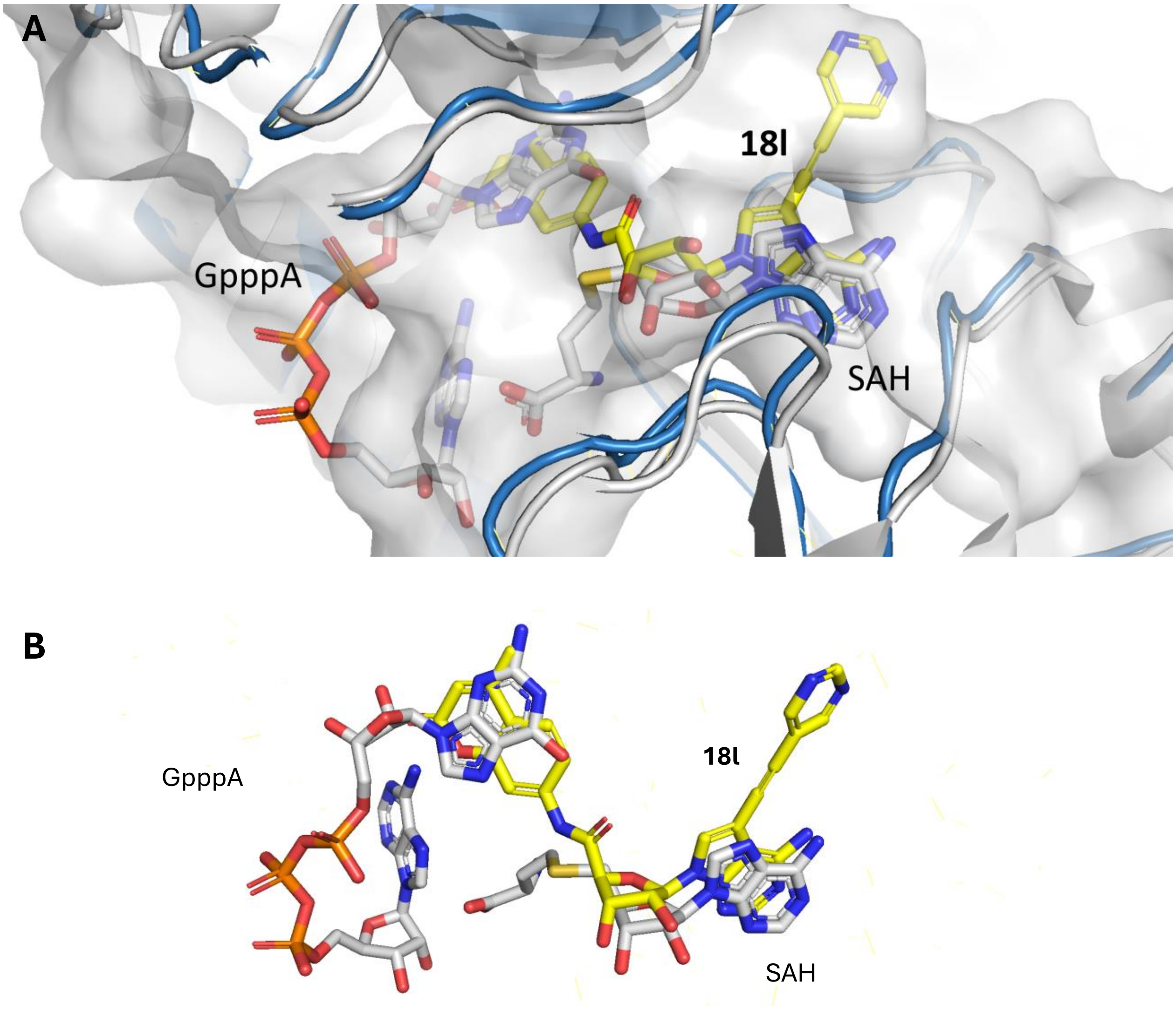

Figure S58. (**A**) Docking pose of **18l** in nsp14 (PDB: 7R2V[12]; protein in blue, inhibitor in yellow) overlayed with nsp14 with cap analog (GpppA, gray) and SAH (PDB: 5C8S[13]; gray). (**B**) Picture analogous to A but omitting the nsp14 protein for clarity.

8. References

1. SAINT, Bruker AXS Inc., Madison, Wisconsin, USA, 2015.
2. A. Altomare, G. Cascarano, C. Giacovazzo, A. Guagliardi, M. C. Burla, G. Polidori and M. Camalli, *Journal of Applied Crystallography*, 1994, **27**, 435.
3. P. W. Betteridge, J. R. Carruthers, R. I. Cooper, K. Prout and D. J. Watkin, *Journal of Applied Crystallography*, 2003, **36**, 1487.
4. R. Shah, A. Strom, A. Zhou, K. M. Maize, B. C. Finzel and C. R. Wagner, *ACS Medicinal Chemistry Letters*, 2016, **7**, 780-784.
5. J. B. Epp and T. S. Widlanski, *The Journal of Organic Chemistry*, 1999, **64**, 293-295.
6. S. De Ornellas, J. M. Slattery, R. M. Edkins, A. Beeby, C. G. Baumann and I. J. Fairlamb, *Organic & Biomolecular Chemistry*, 2015, **13**, 68-72.
7. Y. Luan, L. L. Blazer, H. Hu, T. Hajian, J. Zhang, H. Wu, S. Houliston, C. H. Arrowsmith, M. Vedadi and Y. G. Zheng, *Organic & Biomolecular Chemistry*, 2016, **14**, 631-638.
8. T. Otava, M. Sala, F. Li, J. Fanfrlik, K. Devkota, S. Perveen, I. Chau, P. Pakarian, P. Hobza and M. Vedadi, *ACS Infectious Diseases*, 2021, **7**, 2214-2220.
9. B. Yu, A.-H. Liu, L.-N. He, B. Li, Z.-F. Diao and Y.-N. Li, *Green Chemistry*, 2012, **14**, 957-962.
10. M. Štefek, D. Chalupská, K. Chalupský, M. Zgarbová, A. Dvořáková, P. Krafčíková, A. S. M. Li, M. Šála, M. Dejmek and T. Otava, *ACS Omega*, 2023, **8**, 27410-27418.
11. R. A. Friesner, J. L. Banks, R. B. Murphy, T. A. Halgren, J. J. Klicic, D. T. Mainz, M. P. Repasky, E. H. Knoll, M. Shelley and J. K. Perry, *Journal of Medicinal Chemistry*, 2004, **47**, 1739-1749.
12. A. Czarna, J. Plewka, L. Kresik, A. Matsuda, A. Karim, C. Robinson, S. O'Byrne, F. Cunningham, I. Georgiou and P. Wilk, *Structure*, 2022, **30**, 1050-1054. e1052.
13. Y. Ma, L. Wu, N. Shaw, Y. Gao, J. Wang, Y. Sun, Z. Lou, L. Yan, R. Zhang and Z. Rao, *Proceedings of the National Academy of Sciences*, 2015, **112**, 9436-9441.
